# Rational design and synthesis of new pyrrolone candidates as prospective insecticidal agents against *Culex pipiens* L. Larvae

**DOI:** 10.1038/s41598-024-74011-5

**Published:** 2024-10-18

**Authors:** Mohamed H. Hekal, Ahmed I. Hashem, Fatma S.M. Abu El-Azm, Doaa R. Abdel-Haleem, El-Hady Rafat, Yasmeen M. Ali

**Affiliations:** 1 Department of Chemistry, Faculty of Science, Ain Shams University, Abbassia, Cairo, 11566 Egypt; 2 Entomology Department, Faculty of Science, Ain Shams University, Abbassia, Cairo, 11566 Egypt

**Keywords:** Furanones, Pyrrolones, Pyridazinones, Naphthalene moiety, Insecticidal activity, *Culex pipiens*, Cytochrome P-450 monooxygenase., Enzymes, Chemical tools, NMR spectroscopy

## Abstract

As a result of its high reactivity, furan-2(3*H*)-one derivative **2** can be selected as a versatile and suitable candidate for building of novel nitrogen heterocyclic compounds. Consequently, furan-2(3*H*)-one derivative **2** and some nitrogen nucleophiles were utilized as starting materials for the formation of new pyridazinone and pyrrolone derivatives bearing naphthalene moiety. The continuous buildup of insecticide resistance is the main obstacle facing pest control measures. Pyrrole-based insecticides are a favourable choice due to their unique mode of action and no cross-resistance with traditional neurotoxic insecticides. The larvicidal activities of pyrrolone derivatives were assessed against field and laboratory strains of *Culex pipiens* larvae in comparison with chlorfenapyr (pyrrole insecticide). Compounds **17** (21.05 µg/mL) **> 9** (22.81 µg/mL) **> 15** (24.39 µg/mL) **> 10** (26.76 µg/mL) **> 16** (32.09 µg/mL) were most effective against lab strain of *C. pipiens* larvae relative to chlorfenapyr (25.43 µg/mL). While in field strain, **17** and **15** were the most toxic compounds followed by **9** > **10** > **16** > **2** with LC_50_ of 9.87, 10.76, 11.52, 12.68, 15.32 and 18.37 µg/mL, respectively, compared with chlorfenapyr with 14.03 µg/mL. The cytochrome P-450 monooxygenase activities were significantly increased in treated larvae of lab and field strains relative to untreated. The great variations in toxicity of the synthesized compounds were interpreted by structure-activity relationship study. The pyrrolone derivatives are effective against field and insecticide-resistant strains. Therefore, they are considered promising compounds to be integrated into pest management programs.

**Supplementary Information:**

The online version contains supplementary material available at 10.1038/s41598-024-74011-5.

## Introduction

**Fig. 1:**
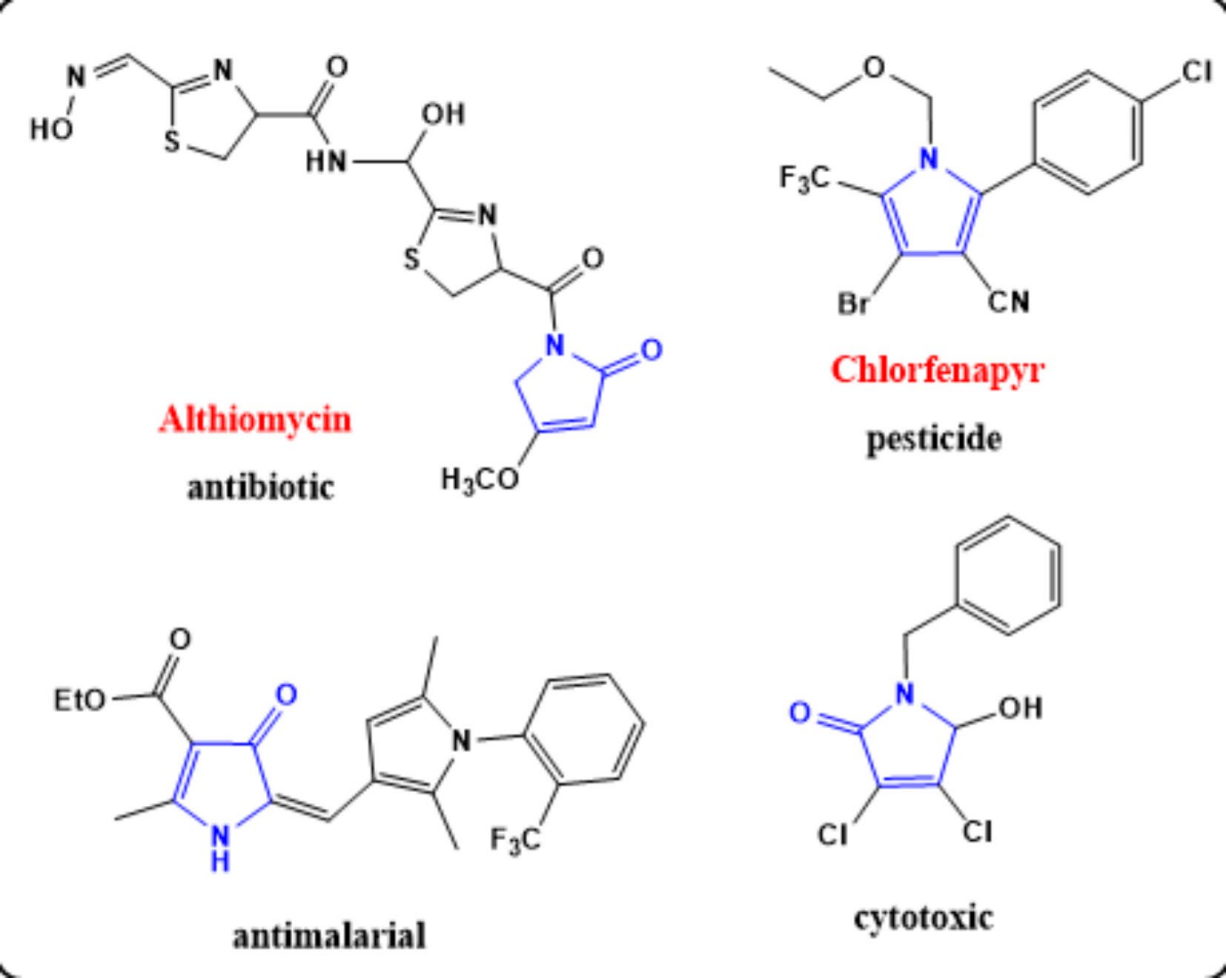
Pyrrole-based compounds with known biological activity.

Due to their facile ring opening by various nucleophiles, 2(3 H)furanones were readily transformed into a diversity of nitrogen heterocyclic systems of pharmacological significance particularly pyrrolones, oxadiazoles, pyridazinones and triazoles^1^. Several sorts of synthetic pyrrolones have a broad range of significant biological activities including antifungal^2^, antibacterial^3^, anti-inflammatory, anticonvulsant^4^, anticancer^5^, antimalarial^6^ and vasodilation^7^. In the last decades, there had been reports on many biological applications of various pyrrole derivatives either from natural resources or chemically synthesized. It was reported that some pyrrole derivatives can act as enzyme inhibitors, acaricidal and insecticidal agents^8^. Furthermore, pyrrolones and pyridazinones have wide applications in medicinal uses. Althiomycin is one of the most important naturally occurring pyrrolone alkaloids. It is isolated from *Streptomyces althioticus* and used as an antibiotic *via* inhibition of protein synthesis^9^**(**Fig. 1**).**

Mosquito species are vectors of severe infectious diseases to humans and animals such as parasites (lymphatic filariasis and malaria) and viruses (Rift Valley fever virus, Zika, chikungunya and avian arbovirus)^10^. Additionally, recent studies detected the transmission of *Rickettsia* (*R*.) *felis* bacteria by mosquitoes^11^. These dreadful diseases have a significant adverse impact on public health. For malaria only, more than 229 million cases and about 409,000 deaths were detected worldwide in 2019^12^. The African regional disease represents approximately 90% of these cases and deaths detected yearly^13^. Also, global warming could increase the transmission risks and related threats of mosquito-borne diseases posed by mosquitoes in temperate regions^14^. Several mosquito species and sub-species have been recorded and identified worldwide including *Culex pipiens* L. (Diptera: Culicidae). *Culex pipiens* is a crucial nuisance and vector of severe human and animal diseases such as lymphatic filariasis and Japanese encephalitis^15^. Testing the susceptibility of mosquito larvae could provide crucial information for the potency assessment of new insecticides and the probability of resistance development in adults^16^.

Chemical application is the fastest, most reliable and effective pest control method. The intensive usage of chemical neurotoxic insecticides threatened their efficacies with the gradual rise of resistance mosquitoes^17^. Chlorfenapyr is a novel pyrrole insecticide class with a completely different mode of action from traditional neurotoxic insecticides used to combat mosquitoes. Pyrroles belong to class 13 of the IRAC classification, uncouplers of oxidative phosphorylation^18^ that exhibited a unique mode of action since it disrupts the energy production of insects. Therefore, this class doesn’t show cross-resistance with conventional insecticides used in vector control programs^19^.

The foregoing reports, coupled with the interest of our research group in the synthesis of biologically active heterocycles, encouraged us to report herein on the synthesis of some novel pyrrolone derivatives with anticipated insecticidal activities. The role of cytochrome P450s in the activation of tested compounds was also studied. Furthermore, the effect of different moieties inserted will be detected via the structure activity relationship study.

### Rational design of the work

**Fig. 2:**
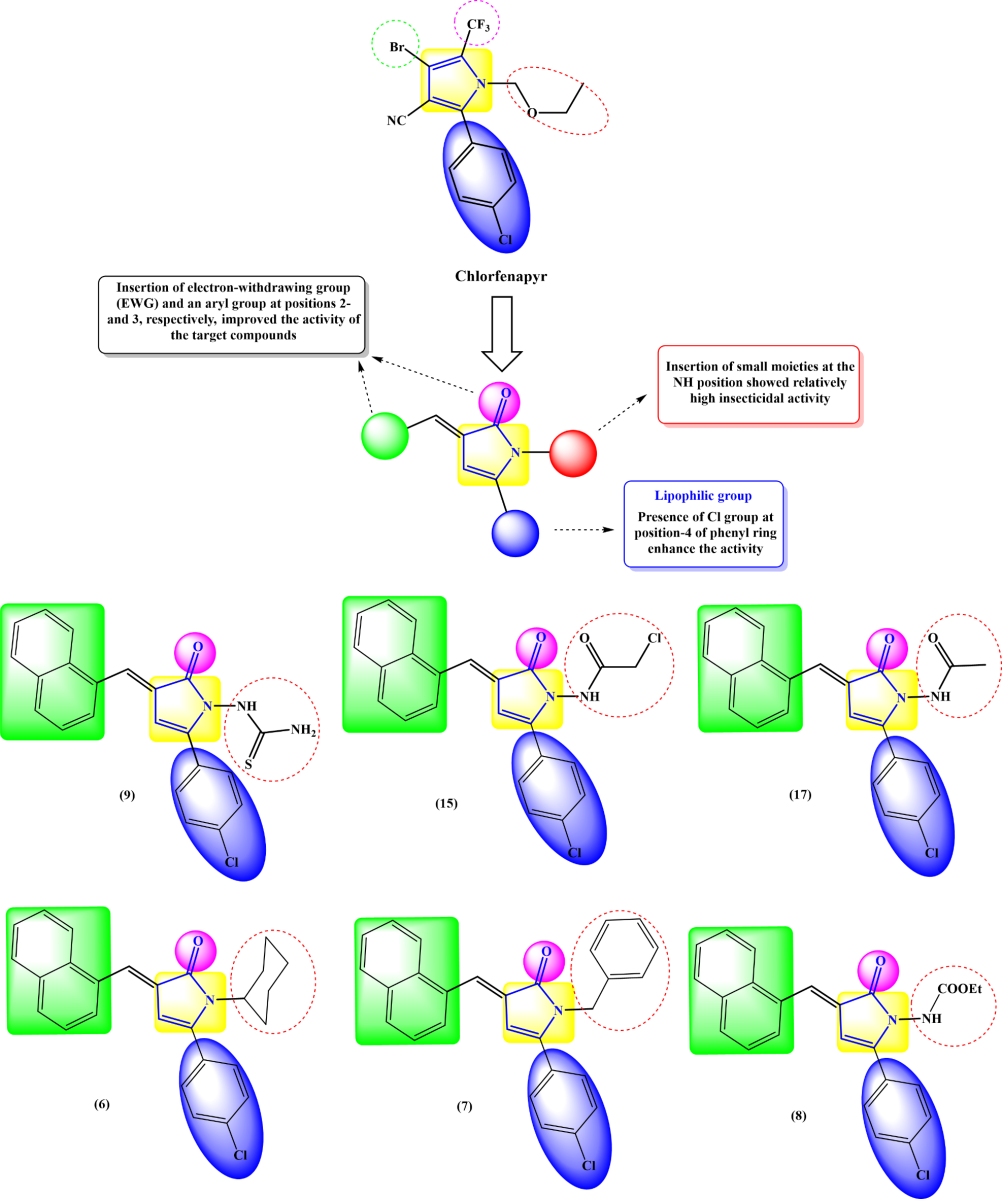
Rational design for the pyrrolone candidates as insecticidal agents based on the previously reported chlorfenapyr (pyrrole insecticide).

Oriented by the insecticidal activity of chlorfenapyr; several chlorfenapyr backbone-based substances have been designed and synthesized, and numerous promising compounds have been obtained. Investigation of the primary structural features of chlorfenapyr **(**Fig. 2**)** disclosed the prominence of the following parts: (i) insertion of electron-withdrawing group (EWG) and an aryl group at positions 2- and 3 of the pyrrole ring, respectively, improved the activity of the compounds; (ii) substitution at position 5- of the pyrrole ring with aryl group substituted at para position with EWG or lipophilic group, such as Cl enhance the insecticidal activity; (iii) introducing short linear alkyl or alkoxy substitutions (small moieties) at the NH position showed substantially high contact or systemic insecticidal effectiveness, while those with longer or branched alkyl insertions (large bulking moieties) tended to decline the efficacy. From these structural features, this study planned to obtain some novel pyrrolone derivatives with anticipated insecticidal activities.

## Results and discussion

### Chemistry

**Fig. 3:**
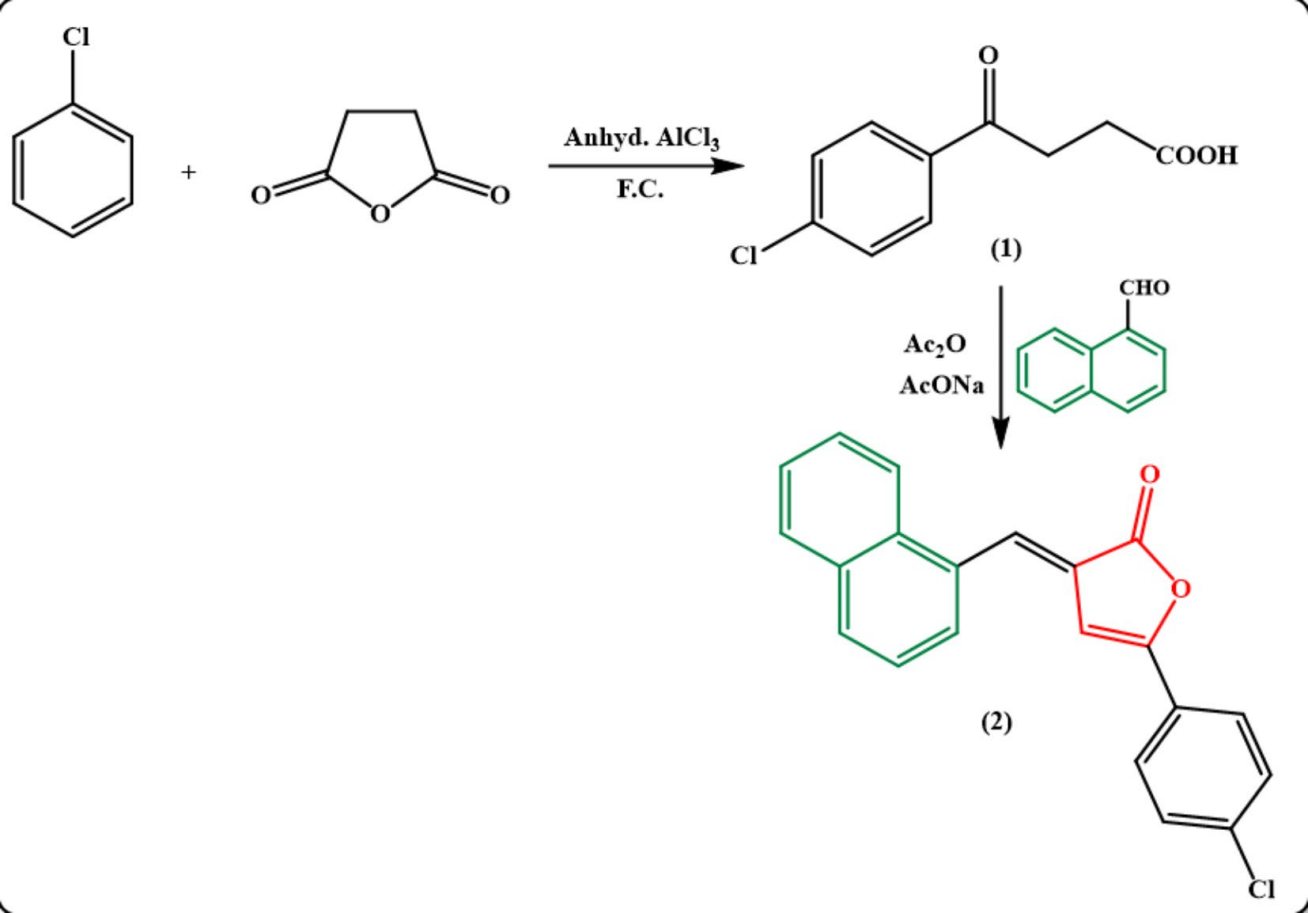
Synthesis of furanone derivative 2 under conventional conditions.

As a continuation of our preceding work on the synthesis of various heterocyclic compounds with evaluation of their applications in biological and medicinal fields^20–39^, the present investigation focuses on the synthesis of new furanone, pyridazinone and pyrrolone derivatives bearing naphthalene moiety with estimation of their insecticidal activities. The required 5-(4-chlorophenyl)-3-(naphthalen-1-ylmethylene)furan-2(3 H)-one (**2**) was prepared by a single step reaction under Perkin reaction conditions. Thus, condensation of 1-naphthaldehyde with 3-aroyl propionic acid **1** in the presence of freshly distilled acetic anhydride and fused sodium acetate resulted in formation of furan-2(3* H*)-one derivative **2** in a good yield *via* anhydrous conditions **(**Fig. 3**)**. The IR spectrum of furanone derivative **2** showed the absorption band for carbonyl group of γ-lactone at υ 1771 cm^− 1^.

**Fig. 4:**
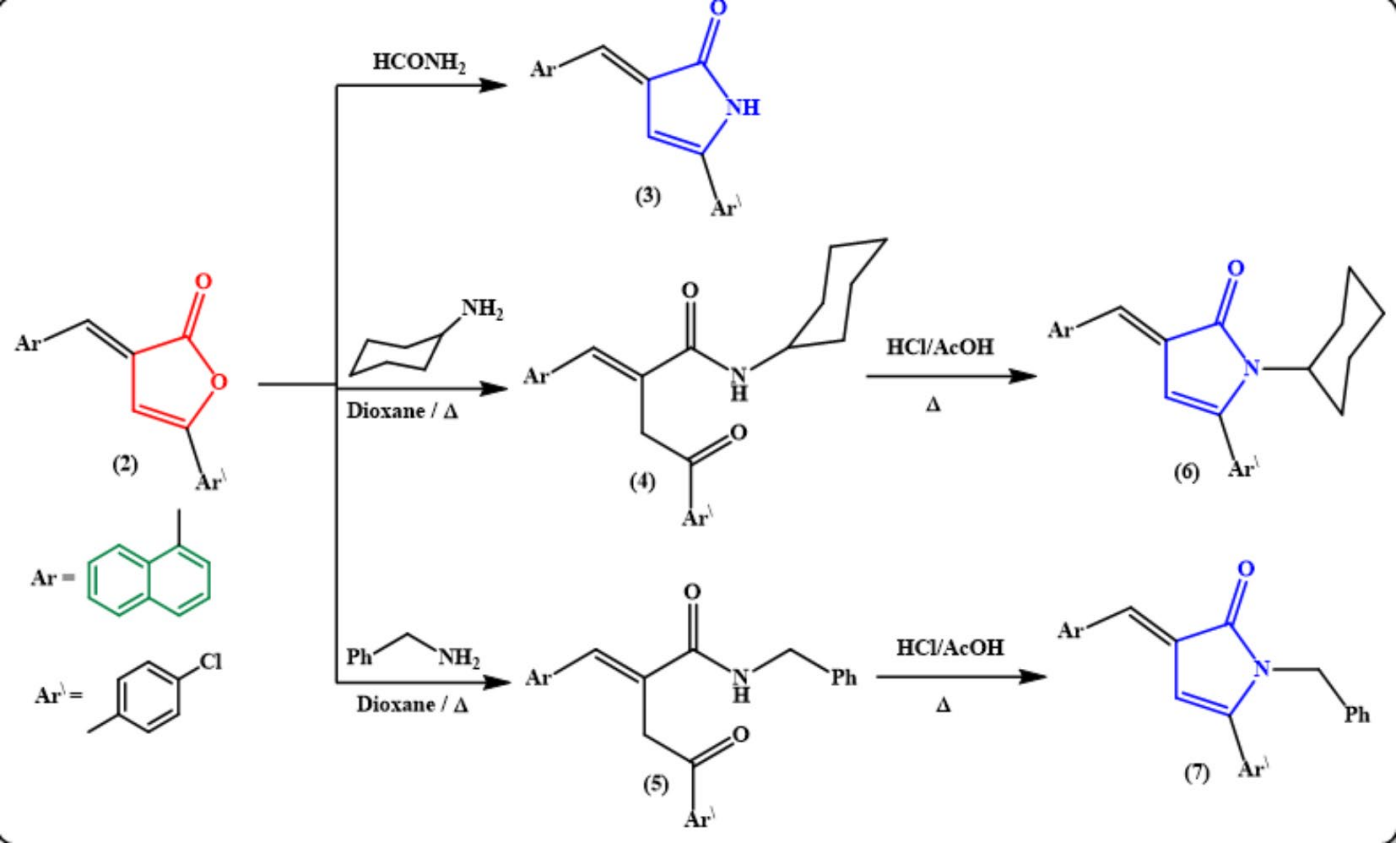
Reaction of furanone derivative 2 with some *N*-nucleophiles.

The functionality in furan-2(3 H)-one derivative **2** renders it valuable candidate for building of novel nitrogen heterocyclic compounds bearing naphthalene moiety as the carbonyl function of γ-lactone is properly situated to allow reactions with common reagents. Thereby, the reaction of furan-2(3* H*)-one derivative **2** with formamide under reflux gave the corresponding 3-arylidene-5-(4-chlorophenyl)-2*H*-pyrrol-2-one derivative **3 (**Fig. 4**)**. The IR spectrum of compound **3** showed the disappearance of the carbonyl group of γ-lactone absorption band and the presence of cyclic imide carbonyl absorption at υ 1695 cm^− 1^, alongside the NH absorption at υ 3154 cm^− 1^. In addition, its ^1^H NMR spectrum displayed an exchangeable singlet signal for NH proton at *δ* 10.71 ppm. Moreover, the interaction between the furanone derivative **2** and cyclohexylamine or benzylamine in boiling dioxane led to ring opening with the formation of the open-chain amide analogs **4** and **5**, respectively **(**Fig. 4**)**. The latter products **4** and **5** were subjected to ring closure to their pyrrolone derivatives **6** and **7** by refluxing in HCl/AcOH mixture **(**Fig. 4**)**. The chemical structures of the amide analogs **4** and **5** and the pyrrolones **6** and **7** were proved from analytical, together with spectral data (cf. Experimental).

**Fig. 5:**
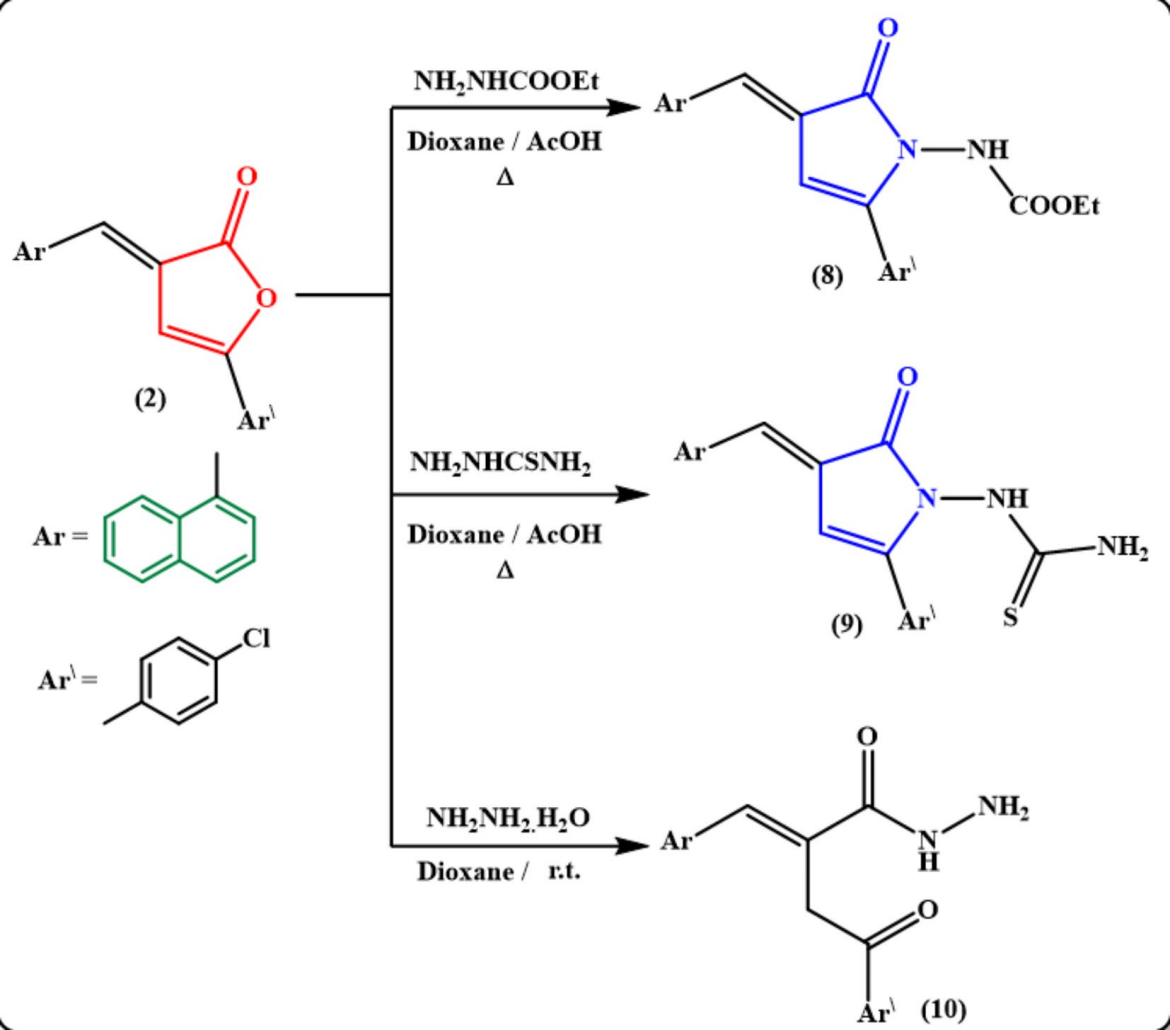
Treatment of furanone derivative 2 with some acid hydrazides and hydrazine hydrate.

Meanwhile, reacting the furanone derivative **2** with some acid hydrazides such as ethyl carbazate and/or thiosemicarbazide under reflux in dioxane/AcOH mixture gave the desired pyrrolone derivatives **8** and **9**, respectively as presented in Fig. 5. The structures of pyrrolone derivatives **8** and **9** were illustrated by IR, ^1^H NMR and ^13^C NMR spectroscopy, as well as elemental analysis. On the other hand, the acid hydrazide **10** was generated by stirring the furan-2(3 H)-one derivative **2** with hydrazine hydrate in dioxane at room temperature **(**Fig. 5**)**. IR spectrum of the hydrazide **10** revealed the presence of absorption bands for NH_2_, NH groups at υ 3312, 3210, 3195 cm^− 1^, and C = O group of amide at υ 1691 cm^− 1^ whereas the absorption band of carbonyl group of γ-lactone was disappeared. Also, ^1^H NMR spectrum of **10** showed two exchangeable signals for NH and NH_2_ protons in addition to the methylene protons. The ^13^C NMR spectrum provided sufficient evidence for the structure of hydrazide **10** (cf. Experimental).

**Fig. 6:**
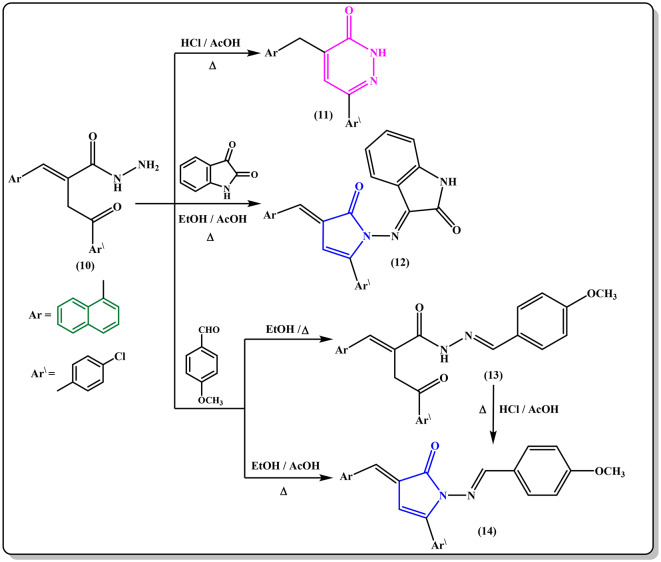
Cyclization of hydrazide derivative 10 to generate pyridazinone and pyrrolone derivatives.

The acid hydrazide **10** was utilized as a principal starting material for the synthesis of some pyridazinone and pyrrolone derivatives bearing a naphthalene moiety. Therefore, the pyridazinone derivative **11** was obtained *via* refluxing the hydrazide derivative **10** in a mixture of glacial acetic acid and hydrochloric acid solution **(**Fig. 6**)**. Condensation of compound **10** with isatin in refluxing EtOH/AcOH mixture yielded the desired pyrrolone derivative **12 (**Fig. 6**)**. Contrarily, condensation of hydrazide **10** with *p*-methoxybenzaldehyde under various conditions was also examined. Thus, conducting the reaction in boiling ethanol yielded the hydrazone derivative **13**. Whereas carrying out the reaction in ethanol/glacial acetic acid mixture under reflux motivated the cyclization step to generate the pyrrolone derivative **14**. The same compound **14** was also achieved by ring closure of the hydrazone **13** in refluxing HCl/AcOH mixture **(**Fig. 6**)**. The chemical structures of obtained compounds **13** and **14** were verified from their spectral and analytical data. Their IR spectra lacked the carbonyl group of γ-lactone absorption band and displayed the amide carbonyl absorption band for the hydrazone derivative **13** at υ 1677 cm^− 1^ and the pyrrolone derivative **14** at υ 1703 cm^− 1^ in addition to the NH absorption at υ 3167 cm^− 1^ for the hydrazone derivative **13**.

**Fig. 7:**
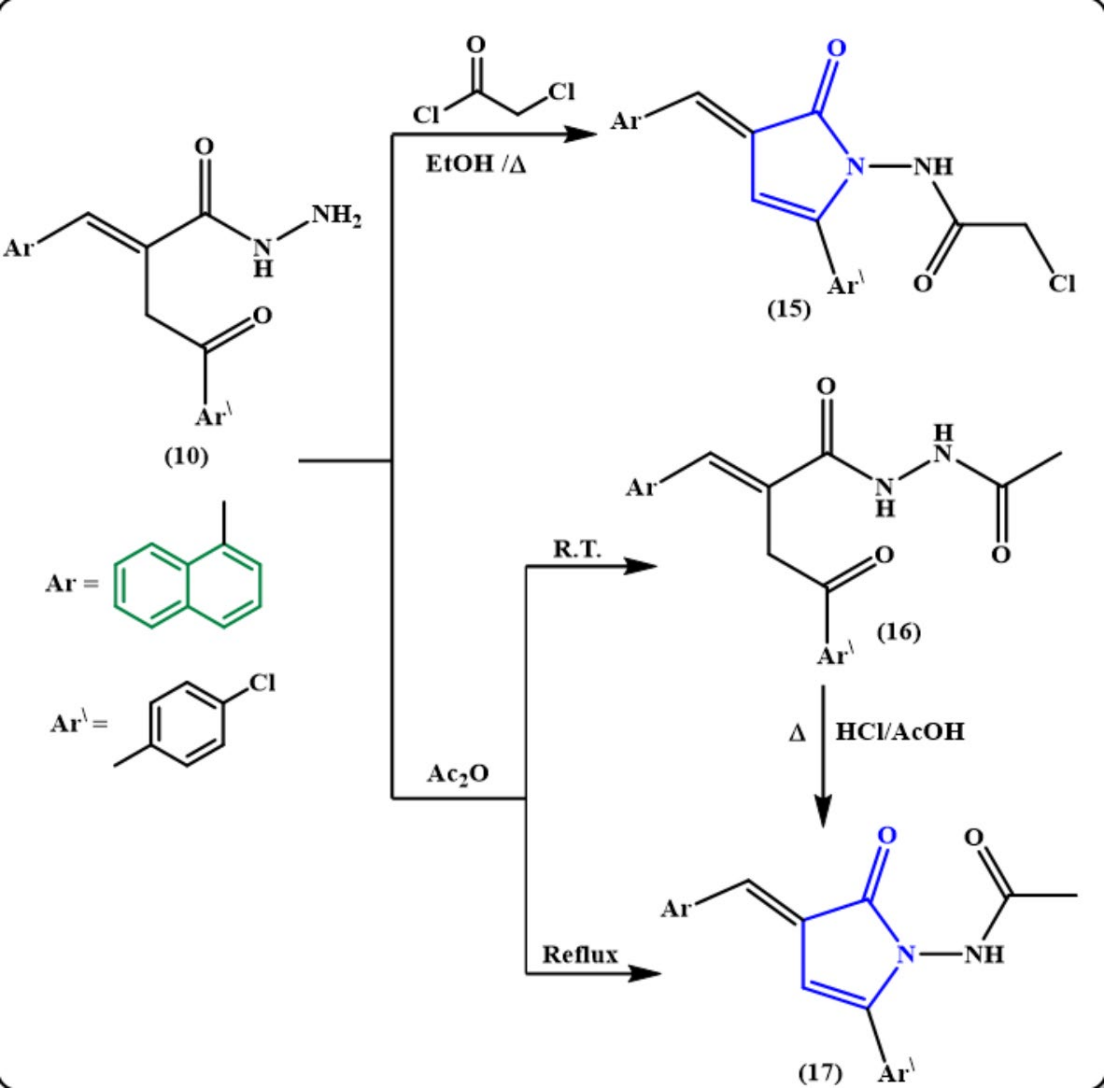
Reaction of hydrazide 10 with one *C*-donors.

Additionally, refluxing a solution of the hydrazide **10** in ethanol with chloroacetyl chloride on a water-bath furnished the pyrrolone derivative **15 (**Fig. 7**)**. The IR spectrum of pyrrolone derivative **15** displayed υNH (br.) at 3242 cm^− 1^ along with υCO at 1736 and 1676 cm^− 1^. Moreover, the existence of one singlet signal for CH_2_ protons of CH_2_Cl at δ 4.25 ppm together with one singlet signal for one NH proton at δ 11.12 ppm beside the aromatic protons in the ^1^H NMR spectrum asserts the structure 15.

Meanwhile, acylation of the hydrazide **10** with acetic anhydride at room temperature provided the *N*-acetyl hydrazide derivative **16** as yellow crystals, while carrying out the reaction under reflux conditions, was simply cyclized into the pyrrolone derivative **17**. Compound **17** was also obtained via refluxing compound **16** in a mixture of CH_3_COOH/HCl (1:1) **(**Fig. 7**).** The structures of the products **16** and **17** were verified by spectroscopic data (cf. Experimental).

### Biological activity

#### Insecticidal activity

The potency of pyrrolone derivatives and chlorfenapyr was assessed at different concentrations against the field and lab strains of the third larval instar of *C. pipiens* and the mortalities were recorded 72 h post-treatment. The LC values and other criteria were estimated and summarized in Tables 1 and 2. In the lab strain, the pyrrolone derivatives showed various activities. Most of the compounds demonstrated significant activities, which exhibited 6.77, 6.24, 5.84, 5.60, 5.32, 4.44 and 3.74 folds than **11**, respectively, against *C. pipiens* larvae, the activity order is as follows: **17** > **9** > **15** > **chlorfenapyr** > **10** > **16** > **5**. Hence **17** and **9** were the most effective compounds with LC_50_ of 21.05 and 22.81 µg/mL, respectively, while **11** was the least active with LC_50_ of 142.56 µg/mL. Also, the larvae were highly susceptible to **15**,** chlorfenapyr**,** 10**,** 16** and **5** with LC_50_ values of 24.39, 25.43, 26.76, 32.09 and 38.07 µg/mL, respectively, against *C. pipiens* larvae. It was noted that compounds **17**,** 9** and **15** were more toxic than chlorfenapyr. On the other hand, some compounds showed low potency, and their order as follows: **13 < 12 < 7 < 14** with LC_50_ values of 122.49, 107.53, 97.74 and 88.65 µg/mL, respectively. In comparison, other compounds showed moderate activities as follows: **2 > 4 > 8 > 6** and **3** with LC_50_ of 44.96, 51.94, 59.87, 67.70, and 79.30 µg/mL, respectively. Moreover, the larval sensitivity to **2**,** 4**,** 8**, **6**, and **3** were 3.17, 2.74, 2.38, 2.10 and 1.79 times than **11**, Table 1. The chi-square values of all tested compounds were significant at *P* < 0.05. The slope values of tested compounds were low, revealing the homogeneity of the tested strain in its response to the pyrrolone derivatives and chlorfenapyr.

**Table 1:** Toxicity of the pyrrolone derivatives against lab strain of third larval instar of *Culex pipiens* after 72 h of exposure.

Compd. (µg/mL)	LC_25_ (^a^F.l. at 95%)	LC_50_ (^a^F.l. at 95%)	LC_90_ (^a^F.l. at 95%)	^b^Slope ± SE	^c^X^2^	*P*	Relative potency
**2**	9.91 (5.71–14.57)	44.96 (34.41–57.02)	203.78 (150.85–306.72)	1.02 ± 0.10	3.46	0.48	3.17
**3**	18.25 (11.87–24.93)	79.30 (62.94–101.09)	1292.79 (752.50–2874.53)	1.05 ± 0.11	1.98	0.73	1.79
**4**	11.65 (6.96–16.74)	51.94 (40.35–65.64)	888.90 (539.58–1852.14)	1.03 ± 0.10	3.80	0.43	2.74
**5**	8.33 (4.61–12.52)	38.07 (28.60–48.56)	682.70 (424.58–1373.62)	1.02 ± 0.10	5.92	0.20	3.74
**6**	15.42 (9.73–21.45)	67.70 (53.46–85.79)	1125.42 (665.67–2440.25)	1.04 ± 0.11	3.74	0.44	2.10
**7**	24.85 (17.39–32.54)	97.74 (78.63–124.32)	1318.79 (788.02–2785.00)	1.13 ± 0.10	3.53	0.47	1.45
**8**	13.34 (8.15–18.91)	59.87 (46.87–75.84)	1037.40 (616.24–2239.60)	1.03 **±** 0.10	3.79	0.43	2.38
**9**	6.31 (1.35–8.91)	22.81 (9.35–35.15)	262.16 (182.99–951.82)	1.20 **±** 0.12	5.22	0.21	6.24
**10**	6.63 (3.70–9.98)	26.76 (19.74–34.19)	378.53 (258.26–650.68)	1.11 ± 0.11	6.04	0.16	5.32
**11**	40.79 (30.90–51.04)	142.56 (115.13–183.99)	1536.23 (926.65–3180.37)	1.24 ± 0.12	3.99	0.40	1
**12**	27.82 (19.83–36.05)	107.53 (86.56–137.46)	1403.08 (835.11–2979.32)	1.14 ± 0.10	3.24	0.51	1.32
**13**	32.15 (23.36–41.20)	122.49 (98.37–158.23)	1555.80 (915.18–3367.55)	1.16 ± 0.12	4.11	0.39	1.16
**14**	21.84 (14.89–29.03)	88.65 (71.07–112.55)	1269.85 (756.01–2703.87)	1.10 ± 0.11	3.22	0.52	1.60
**15**	6.24 (1.38–9.25)	24.39 (10.52–37.72)	324.83 (220.01–1222.15)	1.13 ± 0.11	6.56	0.13	5.84
**16**	7.66 (4.33–11.41)	32.09 (24.04–40.76)	487.90 (322.01–885.74)	1.08 ± 0.10	7.43	0.10	4.44
**17**	6.08 (1.59–8.98)	21.05 (9.52–31.67)	222.86 (152.01–649.20)	1.25 ± 0.12	5.52	0.23	6.77
**Chlorfenapyr**	7.01 (1.55–9.84)	25.43 (10.69–39.69)	293.88 (207.14-1126.99)	1.20 ± 0.12	6.58	0.11	5.60

^a^(F.l.) Fiducial limits.

^b^Slope of the concentration-mortality regression line ± standard error.

^c^*X*^*2*^ chi-square significant at *P* < 0.05.

After 72 h of exposure, the activity of the tested compounds increased against the field strain of *C. pipiens* larvae than the lab strain. **17** and **15** were the most toxic compounds followed by **9** > **10** > **chlorfenapyr** > **16** > **2** with LC_50_ of 9.87, 10.76, 11.52, 12.68, 14.03, 15.32 and 18.37 µg/mL, respectively. It was noticed that **17**, **15**, **9** and **10** were more potent than chlorfenapyr against the field strain of *C. pipiens* larvae. On the other hand, **13** was the least potent compound against the field strain with LC_50_ of 76.61 µg/mL. So, the most efficient compounds **17**, **15**, **9**, **10**, **16** and **2** showed high relative toxicities of 7.76, 7.11, 6.65, 6.04, 5.00 and 4.17 folds than **13**, respectively. On the other hand, **13**, **11**, **12** and **14** were the least efficient compounds with LC_50_ of 76.61, 64.83, 56.28 and 50.65 µg/mL, respectively Table 2. While **5**, **4**, **6**, **8**,** 3** and **7** exhibited moderate activity with LC_50_ of 21.94, 25.65, 29.84, 34.10, 40.46 and 45.61 µg/mL, respectively.

**Table 2:** Toxicity of the pyrrolone derivatives against field strain of third larval instar of *Culex pipiens* after 72 h of exposure.

Compd. (µg/mL)	LC_25_ (^a^F.l. at 95%)	LC_50_ (^a^F.l. at 95%)	LC_90_ (^a^F.l. at 95%)	^b^Slope ± SE	^c^X^2^	*P*	Relative potency
**2**	3.63 (1.94–5.58)	18.37 (13.57–23.83)	400.54 (241.39–841.78)	0.95 ± 0.11	4.38	0.35	4.17
**3**	8.29 (5.25–11.57)	40.46 (31.53–52.62)	822.36 (459.46–1935.58)	0.97 ± 0.10	0.77	0.94	1.89
**4**	5.15 (2.98–7.58)	25.65 (19.58–32.99)	541.13 (316.77–1186.20)	0.96 ± 0.11	2.49	0.64	2.99
**5**	4.33 (2.41–6.53)	21.94 (16.50–28.32)	478.20 (282.56–1035.81)	0.95 ± 0.11	2.36	0.66	3.49
**6**	5.96 (3.54–8.64)	29.84 (22.96–38.48)	636.19 (364.61–442.60)	0.96 ± 0.10	2.08	0.72	2.56
**7**	10.07 (6.71–13.65)	45.61 (35.93–59.05)	804.04 (460.93–1801.31)	1.02 ± 0.13	1.39	0.84	1.67
**8**	6.97 (4.29–9.90)	34.10 (26.48–44.01)	695.70 (397.02–1583.31)	0.97 ± 0.11	1.75	0.78	2.24
**9**	2.74 (1.50–4.19)	11.52 (8.35–14.91)	175.71 (119.97–299.55)	1.08 **±** 0.10	8.13	0.08	6.65
**10**	2.90 (1.58–4.45)	12.68 (9.23–16.41)	208.34 (139.04–368.70)	1.05 ± 0.16	6.74	0.15	6.04
**11**	15.18 (10.79–19.84)	64.83 (51.07–85.63)	1022.01 (576.63–2339.51)	1.07 ± 0.16	2.65	0.61	1.18
**12**	13.01 (9.06–17.18)	56.28 (44.46–73.51)	910.24 (520.08–2042.58)	1.06 ± 0.14	1.84	0.76	1.36
**13**	19.60 (14.55–24.98)	76.61 (60.65–101.22)	1021.48 (590.93–2235.17)	1.13 ± 0.19	2.54	0.63	1
**14**	11.48 (7.84–15.35)	50.65 (40.01–65.79)	849.25 (487.15–1896.27)	1.04 ± 0.14	2.04	0.72	1.51
**15**	2.80 (1.59–4.18)	10.76 (7.89–13.80)	138.76 (98.29 -222.48)	1.15 ± 0.11	9.40	0.05	7.11
**16**	3.33 (1.83–5.07)	15.32 (11.30 -19.76)	278.27 (178.64–524.78)	1.01 ± 0.16	5.99	0.19	5.00
**17**	2.65 (1.53–3.95)	9.87 (7.23–12.67)	119.68 (87.43–182.03)	1.18 ± 0.10	6.20	0.18	7.76
**Chlorfenapyr**	3.11 (1.69–4.75)	14.03 (10.27–18.12)	245.43 (160.17–450.60)	1.03 ± 0.14	5.69	0.22	5.46

^a^(F.l.) Fiducial limits.

^b^Slope of the concentration-mortality regression line ± standard error.

^c^*X*^*2*^ chi-square significant at *P* < 0.05.

#### Biochemical analysis

The results of cytochrome P-450 monooxygenase analysis against *C. pipiens* larvae indicated that the activities were significantly influenced (*P* < 0.01) after treatment with **17**,** 9**,** 15**,** 10**,** 16**,** 5** and chlorfenapyr compared with untreated for lab strain and **17**, **15**, **9**, **10**, **16**,** 2** and chlorfenapyr for field strain.

**Fig. 8:**
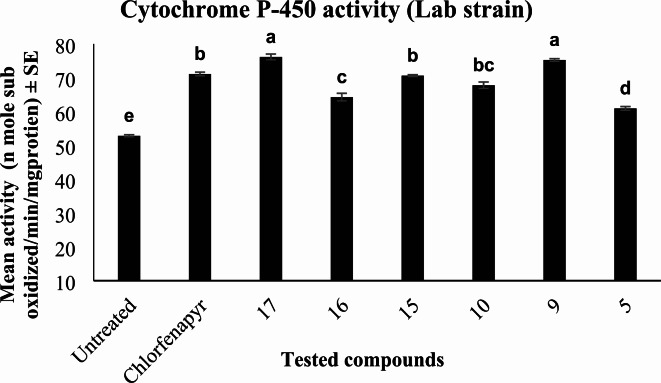
Effect of most toxic pyrrolone derivatives and chlorfenapyr on cytochrome P-450 monooxygenase activity of treated and untreated lab strain of *C. pipiens* larvae. Means with different letters are significantly different (*P* > 0.01) Duncan`s multiple range test.

In lab strain, the cytochrome P-450 activity significantly increased after exposure to all tested compounds, where **17** and **9** greatly enhanced the cytochrome P-450 activity relative to untreated, followed by chlorfenapyr and **15** which showed similar effects. While **16** and **5** showed the lowest changes in cytochrome P-450 activity as shown in Fig. 8.

**Fig. 9:**
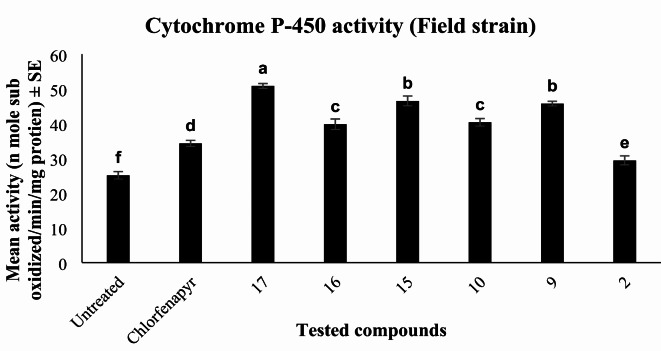
Effect of most toxic pyrrolone derivatives and chlorfenapyr on cytochrome P-450 monooxygenase activity of treated and untreated field strain of *C. pipiens* larvae. Means with different letters are significantly different (*P* > 0.01) Duncan`s multiple range test.

Similarly, in field strain, the tested compounds significantly increased (*P* < 0.01) the activity in the treated larvae relative to untreated. Maximum significant elevation of cytochrome P-450 activity was achieved by **17**. Both **15** and **9** showed similar influence of enzyme activity with a remarkable increase than untreated. As well, the treated larvae exhibited convergent elevation rates post-treatment with **16** and **10** compared to untreated. Also, a significant enhancement in enzyme activity was detected post-treatment with chlorfenapyr and **2**, respectively, relative to untreated Fig. 9.

### Discussion

Pyrrole insecticides impair mitochondrial activity and have been widely employed in urban and agricultural pest control. Chlorfenapyr is considered one of a new generation of malaria-preventative insecticides^40^. This pyrrole insecticide is a highly efficient compound with high residual toxicity, making it an attractive option to reduce insect resistance^41^.

Chlorfenapyr is a pro-insecticide activated and becomes toxic by cytochrome P450s, resulting in improved activity. Chlorfenapyr metabolized by P450s of *An. Gambiae* and *Ae. aegyptia* and a single NADPH was produced. Tralopyril is the main toxic metabolite produced by removing the N-dealkylated group, through P450-mediated oxidation^42–44^. Tralopyril mode of action is a mitochondrial electron transport uncoupler that disrupts the proton gradient across the mitochondrial membranes causing damage to mitochondria [44]. Moreover, it obstructs the production of ATP through oxidative phosphorylation resulting in energy production disorders that ultimately lead to insect death^41,42^.

Unexpectedly, the toxicity of tested compounds revealed higher activity rates in the field than the lab strain of *C. pipiens* larvae which agrees with Oxborough, et al.,^47^ who stated that the application of chlorfenapyr in field trials induced high mortality rates of host-seeking mosquitoes while the in-laboratory bioassay, it exhibited slow-acting mortality levels. Two hypotheses could explain the obtained results, firstly the pyrrole’s mode of action differs significantly from the neurotoxic insecticides, which makes it improbable to show cross-resistance to currently used insecticides^48^. Secondly, metabolic resistance is one of the primary mechanisms of insecticide resistance detected in insects. Neuro-insecticides are used broadly for mosquito larval control or agricultural application and cytochrome P450 monooxygenase production could be increased as one of the metabolic enzymes that break down these insecticides. This detoxification or metabolism process could activate pro-insecticides such as pyrroles and chlorfenapyr in the field rather than the lab strain^49^. Moreover, this potency may increase in nature, mosquito larvae will encounter pyrroles while actively swimming to the water surface to obtain oxygen or search for food when their metabolic enzymes are upregulated. Consequently, it might be exploited to control the resistant insect populations. Generally, a unique mode of action of chlorfenapyr leads to the absence of cross-resistance to conventional insecticides rendering it a potential candidate for inclusion in vector control efforts, especially in areas with high or emergent resistance to other insecticide classes^50^. Chlorfenapyr had a significant insecticidal potency against all developmental stages of *Bradysia odoriphaga* and there were no significant variations in sensitivity between the laboratory and field populations^51^. Raghavendra, et al.,^52^ observed the absence of cross-resistance against chlorfenapyr in multiple insecticides- resistant strains of *An. stephensi* was reared in the laboratory beside field-collected multiple insecticides-resistant *An. culicifacies*. Additionally, Anopheline larvae collected from several locations in rural and urban areas of Cameroon were more susceptible to chlorfenapyr than emerging female adults^16^.

Given that, the activation process depends mainly on insect metabolism. It may take some time to start, but as soon as the conversion begins the insect’s respiration increases, which influences additional conversion of more parent compounds^53^. So, the mortality results were recorded 72 h post-treatment because the toxic efficiency of chlorfenapyr and pyrrole derivatives was slower than that of traditional neurotoxic insecticides. Most mortalities occurred after 24 h to 72 h^19^, Moreover, the chlorfenapyr toxicity increased gradually with time against *Aedes aegypti* larvae, low mortality was observed after 24-h exposure and increased at 72 h of exposure^54^. Chlorfenapyr exerts physiological effects contributing to the significant and extended acute and delayed mortalities detected. Its enzymatic transformation could be quite variable and slow. The uncoupling-oxidative phosphorylation could be affected by cuticular penetrations, food-seeking behaviour, degree of metabolic activity, physical movement of targeted insects, temperature and concentrations of pyrroles^55^. The sulfanyl 5 H-dihydropyrrole derivatives showed good insecticidal activity against larvae of *Ephestia kuehniella* and *Tribolium confusum*. Their residual potencies were influenced by temperature and relative humidity^56^. Six new pyrrole derivatives were revealed as grain protectants against *T. confusum*, *S. oryzae*,* E. kuehniella* and *R. dominica*. The insecticidal efficiency of tested derivatives was moderate and slow acting, where they showed the highest mortality rates 21 days post-treatment. Additionally, the adult population was suppressed, and progeny production was reduced^41^.

Two series of 2-phenylpyrroles exerted moderate larvicidal potency against oriental armyworms and 6b indicated a good acaricidal potency, so 4-cyanopyrrole-2-carboxylate derivatives were promising to be lead compounds for novel pesticides^57^.

The biochemical analyses revealed that the cytochrome P-450 monooxygenase activities were significantly increased in treated larvae of lab and field strains relative to untreated. The populations of insecticide-resistant mosquitoes showed overexpression of P450s that may promote the activation of chlorfenapyr, resulting in improved susceptibility to this insecticide. Furthermore, the pyrethroid-resistant pests such as the tobacco budworm and the cattle horn fly were more susceptible to chlorfenapyr^44^. The increased toxicity of tested compounds caused by increased metabolic activity of cytochrome P-450 leads to the activation of the toxins and increased mortalities of mosquito larvae^55^. Chlorfenapyr was the most toxic tested insecticide against house fly larvae, followed by abamectin and lambda-cyhalothrin, respectively. Chlorfenapyr greatly increased the cytochrome P450 activity relative to other insecticides^58^.

### Structure-activity relationship

The synthesized pyrrolone derivatives resemble chlorfenapyr in the presence of a chlorobenzene ring in the same position at the pyrrole ring, this lipophilic group strengthens the acaricidal and insecticidal activity [46]. In general, the compounds with short linear alkyl or alkoxy substitutions (small bulking moieties) showed substantially high contact or systemic insecticidal effectiveness, while those with longer or branched alkyl insertions (large bulking moieties) tended to decline the efficacy^59^. The insertion of small moieties at the NH position to pyrrolones showed high activity as acetamido group in **17** and chloroacetamido group in **15**, where the acetamide derivatives were widely used as insecticidal agents^60,61^. Also, the insertion of thiourea group in **9** and acetyl, 4-oxobutane hydrazide groups in **16** enhanced the derivatives potency. These results were interpreted by Huang, et al.,^46^ who found that the alkyl or alkoxy substitutions are the most suitable at position N of chlorfenapyr, additionally weak acidic amino groups are desired for the uncoupling action.

Although the good toxicity of **16** with *N*-acetyl hydrazide moiety, the cyclization of this moiety greatly enhanced the activity of compound **17** which showed high toxicity to both field and lab strains. These results are similar to the study of Abdelhamid, et al.,^62^ which revealed that the pyrrole compounds containing the acetohydrazide group showed insecticidal toxicity against the 2nd and 4th larval instars of *S. littoralis* (Boisd.). The insertion of chloroacetylchloride to hydrazide **10** and the formation of pyrrolone with N-acetylchloride in **15** remarkably improved the toxicity to *C. pipiens* larvae where the presence of electron-withdrawing Cl promotes the binding with the target site^63^. The insertion of thiosemicarbazide into pyrrolone significantly increased the activity of **9** due to the presence of electron-withdrawing S increasing the positivity of the amino group^64^.

On the other hand, the compounds with aromatic and long insertions showed moderate to low toxicity according to the bulking of inserted substitutions as methoxybenzylidene in **13**, imino indolinone in **12**, benzyl in **7** and amino methoxybenzylidene in **14** which showed the least toxicity. These results are consistent with Zhao, et al.,^65^ who observed that the aromatic-substituted compounds exerted lower insecticidal potencies against oriental armyworms than short alkyl-substituted compounds. As well as the conversion of the pyrrolone ring to the pyridazine ring in **11** completely changed its toxic characteristics and diminished its potency. All these observations approved the hypothesis of the rational design study.

## Experimental

### Chemistry

All melting points were measured on a Griffin and George melting-point apparatus (Griffin & Georgy Ltd., Wembley, Middlesex, UK) and are uncorrected. IR spectra were recorded on Pye Unicam SP1200 spectrophotometer (Pye Unicam Ltd., Cambridge, UK) by using the KBr wafer technique. ^1^H-NMR spectra were determined on a Varian Gemini 300 MHz on Bruker Avance III using tetramethylsilane as an internal standard (chemical shifts in δ scale), while ^13^C NMR spectra were run at 75 MHz. Elemental analyses were carried out at the Microanalytical Unit, Faculty of Science, Ain Shams University, using a Perkin-Elmer 2400 CHN elemental analyzer (Waltham, MA), and satisfactory analytical data (± 0.4) were obtained for all compounds. The homogeneity of the synthesized compounds was controlled by thin layer chromatography (TLC), using aluminum sheet silica gel F_254_ (Merck). The structures of the compounds in **(**Figs. 1, 2, 3, 4, 5, 6 and 7**)** were drawn in Chem Office Ultra 2004 software, version 8, https://chemoffice-ultra-2004.software.informer.com.

#### ***(E)-5-(4-chlorophenyl)-3-(naphthalen-1-ylmethylene)furan-2(3 H)-one 2***:

A mixture of 4-(4-chlorophenyl)-4-oxobutanoic acid **1** (2.12 g, 10 mmol) and 1-naphthaldehyde (1.3 mL, 10 mmol) was fused in the presence of sodium acetate (0.8 g, 10 mmol) and acetic anhydride (2 mL) on a hot plate then heated on a water bath for 1 h. The crude material was filtered off, washed with ethanol and then recrystallized from dioxane to give **2** as orange crystals; mp 258–260 °C, yield 60%. IR (υ/cm^− 1^): 3049 (aryl-H), 1771 (C = O_lactone_), 1618 (C = C). ^1^H-NMR (DMSO-*d*_*6*_) δ (ppm): 7.49 (s, 1H, CH _Furanone_), 7.55–8.23 (m, 12 H, 11 Ar-H + CH=). Anal. calcd. for C_21_H_13_ClO_2_ (332.78): C, 75.79; H, 3.94; Cl, 10.65. Found: C, 75.66; H, 3.74; Cl, 10.73.

#### ***(E)-5-(4-chlorophenyl)-3-(naphthalen-1-ylmethylene)-1***,***3-dihydro-2 H-pyrrol-2-one 3***:

A solution of furanone derivative **2** (3.3 g, 10 mmol) in formamide (10 mL) was heated under reflux for 1 h. The obtained solid was collected, washed with diethyl ether, and then recrystallized from EtOH to give **3** as red-orange crystals; mp 294–296 °C, yield 65%. IR (υ/cm^1^): 3154 (NH), 1695 (C = O_lactam_), 1614 (C = C). ^1^H-NMR (DMSO-*d*_*6*_) δ (ppm): 6.80 (s, 1H, CH_pyrrolone_), 7.50–7.65 (m, 5 H, Ar-H), 7.86–8.02 (m, 6 H, Ar-H), 8.18 (d, 1H, Ar-H, *J* = 8.4 Hz), 10.71 (s, 1H, NH, exchangeable with D_2_O). ^13^C-NMR (DMSO-*d*_*6*_) δ (ppm): 98.1, 123.3, 125.9, 126(2), 127(2), 128(4), 129.9, 131(2), 132.8, 133.3, 134.2, 145.1, 170.4. Anal. calcd. for C_21_H_14_ClNO (331.80): C, 76.02; H, 4.25; Cl, 10.68; N, 4.22. Found: C, 76.21; H, 4.16; Cl, 10.55; N, 4.29.

#### ***(E)-4-(4-chlorophenyl)-N-cyclohexyl-2-(naphthalen-1-ylmethylene)-4-oxobutanamide 4***:

Cyclohexyl amine (1 mL, 10 mmol) was added dropwise to a solution of furanone derivative **2** (3.3 g, 10 mmol) in dioxane (20 mL) at ambient temperature and then refluxed for 10 h. After cooling, the deposited solid was collected by filtration, dried and then recrystallize from EtOH/dioxane to give **4** as white crystals; mp 266–268 °C, yield 75%. IR (υ/cm^− 1^): 3161 (NH), 2938, 2848 (C-H, aliphatic), 1669 (C = O), 1640 (C = C).^1^H-NMR (DMSO-*d*_*6*_) δ (ppm): 0.85–1.81 (m, 10H_cyclohexyl_), 3.25 (s, 2 H, CH_2_), 3.71 (m, 1H, NH-*CH*_cyclohexyl_), 6.81 (br s, 1H, NH, exchangeable with D_2_O), 7.41–8.15 (m, 11 H, Ar-H + 1H_olefinic_).^13^C-NMR (DMSO-*d*_*6*_) δ (ppm): 24.3, 25(3), 29(2), 31.5, 32.5, 38.7, 53.0, 123.5, 124(2), 125(2), 126(3), 127(2), 128(4), 130(2), 131(3), 132.4, 133(3), 134.4, 135.2, 138.1, 143.2, 166(2), 196.7. Anal. calcd. for C_27_H_26_ClNO_2_ (431.96): C, 75.08; H, 6.07; Cl, 8.21; N, 3.24. Found: C, 75.18; H, 6.00; Cl, 8.39; N, 3.16.

#### ***(E)-N-benzyl-4-(4-chlorophenyl)-2-(naphthalen-1-ylmethylene)-4-oxobutanamide 5***:

A mixture of furanone derivative **2** (3.3 g, 10 mmol) and benzyl amine (1.1 mL, 10 mmol) in dioxane (20 mL) was heated under reflux for 12 h. The obtained oil after concentration of the reaction mixture was solidified by boiling with ethanol. The precipitated solid was filtered off, dried and recrystallized from dioxane to give **5** as white crystals; mp 240–242 °C, yield 60%. IR (υ/cm^1^): 3315 (br NH), 3051 (C-H, aromatic), 2925, 2851 (C-H, aliphatic), 1679 (C = O), 1649 (C = C). ^1^H-NMR (DMSO-*d*_*6*_) δ (ppm): 3.35 (s, 2 H, =C-*CH*_*2*_-CO), 4.26, 4.36 (d, d, 2 H, NH-*CH*_*2*_-, *J* = 15.3 Hz), 6.92 (s, 1H, NH, exchangeable with D_2_O), 7.18–8.16 (m, 16 H, Ar-H + 1H_olefinic_). ^13^C-NMR (DMSO-*d*_*6*_) δ (ppm): 38(2), 43(2), 123.4, 125(2), 126(4), 127(2), 128(2), 129.0, 131(2), 132(2), 133.2, 138.1, 142.1, 167.7. Anal. calcd. for C_28_H_22_ClNO_2_ (439.94): C, 76.44; H, 5.04; Cl, 8.06; N, 3.18. Found: C, 76.52; H, 5.17; Cl, 8.21; N, 3.11.

#### ***Synthesis of compounds 6 and 7***

A mixture of acetic acid (10 mL) and concentrated hydrochloric acid (1 mL) was added to compound **4** and/or **5** (10 mmol) then refluxed for 15 min. The deposited solid while heating was collected, washed with diethyl ether, dried and recrystallized from appropriate solvent to give **6** and **7**, respectively.

#### ***(E)-5-(4-chlorophenyl)-1-cyclohexyl-3-(naphthalen-1-ylmethylene)-1***,***3-dihydro-2 H-pyrrol-2-one 6***:

Recrystallized from EtOH as orange crystals; mp 208–210 °C, yield 70%. IR (υ/cm^− 1^): 3050 (C-H, aromatic), 2954, 2926, 2864 (C-H, aliphatic), 1685 (C = O), 1608 (C = C). ^1^H-NMR (DMSO-*d*_*6*_) δ (ppm): 1.11 (m, 3 H, CH_cyclohexyl_), 1.56–1.75 (m, 5 H, CH_cyclohexyl_), 2.26 (m, 2 H, CH_2cyclohexyl_), 3.4 (m, 1H, NH-*CH*_cyclohexyl_), 6.20 (s, 1H _pyrrolone ring_), 7.50–7.66 (m, 8 H, Ar-H), 7.89–8.17 (m, 4 H, 3 Ar-H + CH=), ^13^C-NMR (DMSO-*d*_*6*_) δ (ppm): 24.9, 25.7, 28.6, 29(2), 63.4, 101.2, 120(2), 123.5, 126(2), 127(3), 128(2), 129(3), 131.6, 133.4, 134.3, 143.9, 149.7, 169.2. Anal. calcd. for C_27_H_24_ClNO (413.95): C, 78.34; H, 5.84; Cl, 8.56; N, 3.38. Found: C, 78.23; H, 5.92; Cl, 8.70; N, 3.18.

#### ***(E)-1-benzyl-5-(4-chlorophenyl)-3-(naphthalen-1-ylmethylene)-1***,***3-dihydro-2 H-pyrrol-2-one 7***:

Recrystallized from EtOH as orange crystals; mp 220–222 °C, yield 80%. IR (υ/cm^− 1^): 3057 (C-H, aromatic), 2911 (C-H, aliphatic) 1701 (C = O _pyrrolone_). ^1^H-NMR (DMSO-*d*_*6*_) δ (ppm): 4.89 (s, 2 H, *CH*_*2*_-Ph), 6.41 (s, 1H, =CH _pyrrolone_), 7.02–7.05 (m, 2 H, Ar-H), 7.17–7.29 (m, 3 H, Ar-H), 7.34–7.50 (m, 4 H, Ar-H), 7.57–7.67 (m, 3 H, Ar-H), 7.97–8.03 (m, 3 H, Ar-H), 8.13 (s, 1H, CH_olefinic_), 8.20 (d, 1H, Ar-H, *J* = 8.1 Hz).^13^C-NMR (DMSO-*d*_*6*_) δ (ppm): 43.8, 101.3, 120.7, 123.4, 125.9, 126.4, 127(2), 128(4), 129.4, 130.2, 131(2), 134.2, 137.5, 148.3, 169.0. Anal. calcd. for C_28_H_20_ClNO (421.92): C, 79.71; H, 4.78; Cl, 8.40; N, 3.32. Found: C, 79.80; H, 4.69; Cl, 8.53; N, 3.12.

#### ***Ethyl (E)-(5-(4-chlorophenyl)-3-(naphthalen-1-ylmethylene)-2-oxo-2***,***3-dihydro-1 H-pyrrol-1-yl)carbamate 8***:

A mixture of furanone derivative **2** (3.3 g, 10 mmol), ethyl carbazate (1 g, 10 mmol) in dioxane (20 mL) and glacial acetic acid (10 mL) was heated under reflux for 15 h. After evaporation of the excess solvent, the precipitated solid was collected by filtration, washed with diethyl ether, dried and then recrystallized from EtOH to give **8** as yellow crystals; mp 184–186 °C, yield 67%. IR (υ/cm^1^): 3260 (NH), 1736 (C = O_ester_), 1704 (C = O _pyrrolone_), 1621 (C = C). ^1^H-NMR (DMSO-*d*_*6*_) δ (ppm): 1.17 (t, 3 H, CH_2_*CH*_*3*_), 4.07 (q, 2 H, *CH*_*2*_CH_3_), 6.60 (s, 1H, CH_pyrrolone_), 7.53–7.60 (m, 7 H, Ar-H), 8.00-8.18 (m, 5 H, 4 Ar-H + 1CH=), 10.15 (br s, 1H, NH, exchangeable with D_2_O). ^13^C-NMR (DMSO-*d*_*6*_) δ (ppm): 14.3, 61.4, 99.8, 123.4, 125.9, 126.5, 127(2), 128(3), 129(2), 130.4, 131(2), 133.3, 134.5, 146.8, 155.4, 168.4. Anal. calcd. for C_24_H_19_ClN_2_O_3_ (418.88): C, 68.82; H, 4.57; Cl, 8.46; N, 6.69. Found: C, 68.74; H, 4.42; Cl, 8.39; N, 6.79.

#### ***(E)-1-(5-(4-chlorophenyl)-3-(naphthalen-1-ylmethylene)-2-oxo-2***,***3-dihydro-1 H-pyrrol-1-yl)thiourea 9***:

A mixture of furanone derivative **2** (3.3 g, 10 mmol) and thiosemicarbazide (0.9 g, 10 mmol) in dioxane (20 mL) and glacial acetic acid (10 mL) was refluxed for 14 h. After evaporation of the excess solvent, the precipitated solid was collected by filtration, washed with diethyl ether, dried and then recrystallized from EtOH to give **9** as red-orange crystals; mp 235–237 °C, yield 60%. IR (υ/cm^1^): 3450, 3239, 3191 (NH_2_, NH), 1701 (C = O _pyrrolone_). ^1^H-NMR (DMSO-*d*_*6*_) δ (ppm): 6.61 (s, 1H CH_pyrrolone_), 7.51–8.30 (m, 11 H, Ar-H + 1H, CH_olefinic_+ 2 H, NH_2_, exchangeable with D_2_O), 10.66 (br s, 1H, NH, exchangeable with D_2_O). ^13^C-NMR (DMSO-*d*_*6*_) δ (ppm): 100.5, 123.4, 125.9, 126.5, 127.3, 128(4), 129.0, 131(2), 132.7, 133.3, 134.25, 134.4, 146.0, 147.2, 168.3, 182.5. Anal. calcd. For C_22_H_16_ClN_3_OS (405.90): C, 65.10; H, 3.97; Cl, 8.73; N, 10.35; S, 7.90. Found: C, 65.16; H, 3.86; Cl, 8.56; N, 10.49; S, 8.12.

#### ***(E)-4-(4-Chlorophenyl)-2-(naphthalen-1-ylmethylene)-4-oxobutanehydrazide 10***:

Hydrazine hydrate (1 mL, 20 mmol) was added dropwise to a stirred solution of furanone derivative **2** (3.3 g, 10 mmol) in dioxane (30 mL) at ambient temperature. The reaction mixture was further stirred for 1 h, then poured into water. The deposited solid was filtered off, dried and recrystallized from EtOH to give **10** as white crystals; mp 178–180 °C, yield 72%. IR (υ/cm^1^): 3312, 3210, 3195 (NH_2_, NH), 1691 (C = O). ^1^H-NMR (DMSO-*d*_*6*_) δ (ppm): 3.16 (s, 2 H, CH_2_), 4.52 (br s, 2 H, NH_2_, exchangeable with D_2_O), 6.75 (s, 1H, NH, exchangeable with D_2_O), 7.40–8.11 (m, 11 H, Ar-H + 1H_olefinic_). ^13^C-NMR (DMSO-*d*_*6*_) δ (ppm): 42.6, 123.4, 125(2), 126(3), 127.7, 128(3), 130.8, 131(2), 132.2, 133.2, 142.4, 166.2. Anal. calcd. for C_21_H_17_ClN_2_O_2_ (364.83): C, 69.14; H, 4.70; Cl, 9.72; N, 7.68. Found: C, 69.23; H, 4.79; Cl, 9.85; N, 7.49.

#### *6-(4-Chlorophenyl)-4-(naphthalen-1-ylmethyl)pyridazin-3(2 H)-one 11*

A solution of hydrazide derivative **10** (3.6 g, 10 mmol) in sodium hydroxide (10%, 30 mL) was heated under reflux for 10 h. After cooling, the reaction mixture was acidified with cold dilute hydrochloric acid and the deposited solid was collected then recrystallized from EtOH/dioxane to give **11** as white crystals; mp 290–292 °C, yield 65%. IR (υ/cm^1^): 3200, 3129 (NH), 1653 (C = O), 1604 (C = N). ^1^H-NMR (DMSO-*d*_*6*_) δ (ppm): 4.32 (s, 2 H, CH_2_), 7.39–8.07 (m, 11 H, Ar-H + 1H _pyridazine_), 13.32 (br. s, 1H, NH, exchangeable with D_2_O). ^13^C-NMR (DMSO-*d*_*6*_) δ (ppm): 31.7, 123.9, 125(2), 126.4, 127(3), 128(2), 131.5, 133(3), 134.1, 142(2), 160.7. Anal. calcd. for C_21_H_15_ClN_2_O (346.81): C, 72.73; H, 4.36; Cl, 10.22; N, 8.08. Found: C, 72.59; H, 4.19; Cl, 10.33; N, 7.95.

#### ***(E)-3-(((E)-5-(4-chlorophenyl)-3-(naphthalen-1-ylmethylene)-2-oxo-2***,***3-dihydro-1 H-pyrrol-1-yl)imino)indolin-2-one 12***:

A mixture of the hydrazide **10** (3.6 g, 10 mmol) and isatin (1.4 g, 10 mmol) in absolute ethanol (20 mL) containing glacial acetic acid (10 mL) was refluxed for 2 h. The obtained solid while heating was filtered off, dried and then recrystallized from dioxane to give **12** as orange crystals; mp 292–294 °C, yield 60%. IR (υ/cm^1^): 3165 (NH), 1723 (C = O). ^1^H-NMR (DMSO-*d*_*6*_) δ (ppm): 6.91–8.22 (m, 17 H, 1H _pyrrolone ring_ + 1H_olefinic_ + 15Ar-H), 11.04 (s, 1H, NH, exchangeable with D_2_O). ^13^C-NMR (DMSO-*d*_*6*_) δ (ppm): 102.5, 103.5, 104.2, 111.0, 116.8, 117.7, 120(2), 122(2), 123(2), 126.1, 127.5, 128(2), 129(3), 130.9, 131(2), 133.4. 134(2) 140.7, 144.2, 145.3, 147(2), 152.4, 153.0, 155.6, 163.5, 164.1. Anal. calcd. for C_29_H_18_ClN_3_O_2_ (475.93): C, 73.19; H, 3.81; Cl, 7.45; N, 8.83. Found: C, 73.06; H, 3.59; Cl, 7.53; N, 8.77.

#### ***(E)-4-(4-Chlorophenyl)-N’-((Z)-4-methoxybenzylidene)-2-(naphthalen-1-ylmethylene)-4-oxobutanehydrazide 13***:

A mixture of the hydrazide **10** (3.6 g, 10 mmol) and *p*-methoxybenzaldehyde (1.2 mL, 10 mmol) in absolute ethanol (20 mL) was refluxed for 6 h. The deposited solid on hot was collected, dried and then recrystallized from dioxane to give **13** as pale-yellow crystals; mp 222–224 °C, yield 70%. IR (υ/cm^1^): 3167 (NH), 3075 (C-H, aromatic), 2934, 2836 (C-H, aliphatic), 1677 (C = O), 1638 (C = N), 1611 (C = C). ^1^H-NMR (DMSO-*d*_*6*_) δ (ppm): 3.15 (s, 2 H, CH_2_), 3.76 (s, 3 H, OCH_3_), 6.78 (s, 1H, NH, exchangeable with D_2_O), 6.96–8.12 (m, 15 H, Ar-H), 9.06 (s, 1H, N = C*H*). ^13^C-NMR (DMSO-*d*_*6*_) δ (ppm): 43.2, 55.4, 112.1, 123.5, 125.7, 126.4, 127(2), 128(4), 129(2), 130.5, 131(2), 132.5, 133.3, 140.5, 142.6, 145.9, 153.8, 161.4, 164.4. Anal. calcd. for C_29_H_23_ClN_2_O_3_ (482.96): C, 72.12; H, 4.80; Cl, 7.34; N, 5.80. Found: C, 72.04; H, 4.64; Cl, 7.48; N, 5.87.

#### ***(E)-5-(4-Chlorophenyl)-1-(((E)-4-methoxybenzylidene)amino)-3-(naphthalen-1-ylmethylene)-1***,***3-dihydro-2 H-pyrrol-2-one 14***:

Method A: A mixture of the hydrazide **10** (3.6 g, 10 mmol) and *p*-methoxybenzaldehyde (1.2 ml, 10 mmol) in absolute ethanol (20 mL) in the presence of glacial acetic acid (10 mL) was heated under reflux for 30 min. The precipitated solid while heating was filtered off, dried and recrystallized from EtOH to give **14** as orange crystals; mp 242–244 °C, yield 82%. IR (υ/cm^1^): 3047 (C-H, aromatic), 2932, 2836 (C-H, aliphatic), 1703 (C = O), 1622 (C = N), 1608 (C = C). ^1^H-NMR (DMSO-*d*_*6*_) δ (ppm): 3.81 (s, 3 H, CH_3_), 6.68 (s, 1H _pyrrolone ring_), 7.05–8.20 (m, 15 H, Ar-H + 1H_olefinic_), 9.39 (s, 1H, N = C*H*). Anal. calcd. for C_29_H_21_ClN_2_O_2_ (464.95): C, 74.92; H, 4.55; Cl, 7.62; N, 6.03. Found: C, 74.78; H, 4.51; Cl, 7.83; N, 6.13.

Method B: A solution of compound **13** (4.8 g, 10 mmol) in glacial acetic acid (20 mL) containing concentrated hydrochloric acid (1 mL) was heated under reflux for 30 min. The precipitated solid while heating was filtered off, dried and recrystallized from EtOH to give **14** as orange crystals; yield 85%.

#### ***(E)-2-Chloro-N-(5-(4-chlorophenyl)-3-(naphthalen-1-ylmethylene)-2-oxo-2***,***3-dihydro-1H-pyrrol***

To a solution of the hydrazide **10** (3.6 g, 10 mmol) in ethanol (20 mL), chloroacetyl chloride (0.8 mL, 10 mmol) was added dropwise and then refluxed on water-bath for 2 h. The reaction mixture was then poured into water and the obtained solid was collected, dried and recrystallized from petroleum ether 80–100 °C to give **15** as red crystals; mp 218–220 °C, yield 64%. IR (υ/cm^1^): 3242 (NH), 3043 (C-H, aromatic), 1736 (C = O_pyrrolone ring_), 1676 (C = O_amide_), 1612 (C = C). ^1^H-NMR (DMSO-*d*_*6*_) δ (ppm): 4.25 (s, 2 H, *CH*_*2*_Cl), 6.64 (s, 1H_pyrrolone ring_), 7.50–8.23 (m, 11 H, Ar-H + 1H_olefinic_), 11.12 (s, 1H, NH, exchangeable with D_2_O).^13^C-NMR (DMSO-*d*_*6*_) δ (ppm): 40.4, 100.1, 123.4, 125.8, 126.5, 127(2), 128 (3), 129.6, 130.4, 131(2), 133.3, 134.5, 146.7, 165.8, 167.9. Anal. calcd. for C_23_H_16_Cl_2_N_2_O_2_ (423.29): C, 65.26; H, 3.81; Cl, 16.75; N, 6.62. Found: C, 65.35; H, 3.70; Cl, 16.68; N, 6.41.

#### ***(E)-N’-Acetyl-4-(4-chlorophenyl)-2-(naphthalen-1-ylmethylene)-4-oxobutanehydrazide 16***:

The hydrazide derivative **10** (3.6 g, 10 mmol) in acetic anhydride (30 mL) was stirred at room temperature for 3 h. The separated yellow solid was filtered off, wash with diethyl ether, dried and then recrystallized from EtOH to give **16** as yellow crystals; mp 230–232 °C, yield 65%. IR (υ/cm^1^): 3276, 3189 (NH), 3022 (C-H, aromatic), 2912, 2808 (C-H, aliphatic), 1713, 1687, 1667 (C = O), 1644 (C = C). ^1^H-NMR (DMSO-*d*_*6*_) δ (ppm): 1.85 (s, 3 H, CH_3_), 3.35 (s, 2 H, CH_2_), 6.88 (s, 1H, NH-*NH*-COCH_3_, exchangeable with D_2_O), 7.38–8.13 (m, 11 H, Ar-H + 1H_olefinic_), 9.86 (s, 1H, *NH*-NH-COCH_3_, exchangeable with D_2_O). ^13^C-NMR (DMSO-*d*_*6*_) δ (ppm): 20.5, 42.7, 123.4, 125.5, 126 (3), 127 (2), 128(2), 129(2), 131(2), 132.5, 133.2, 141.2, 165.3, 168.6. Anal. calcd. for C_23_H_19_ClN_2_O_3_ (406.87): C, 67.90; H, 4.71; Cl, 8.71; N, 6.89. Found: C, 67.96; H, 4.62; Cl, 8.86; N, 7.06.

#### ***(E)-N-(5-(4-chlorophenyl)-3-(naphthalen-1-ylmethylene)-2-oxo-2***,***3-dihydro-1 H-pyrrol-1-yl)acetamide 17***:

Method A: The hydrazide derivative **10** (3.6 gm, 10 mmol) in acetic anhydride (30 mL) was heated under reflux for 30 min. The precipitated solid while heating was filtered off, dried and recrystallized from EtOH to give **17** as yellow crystals; mp 264–266 °C, yield 68%. IR (υ/cm^1^): 3230 (NH), 3018 (C-H, aromatic), 2962, 2885 (C-H, aliphatic), 1728 (C = O_pyrrolone ring_), 1674 (C = O_amide_), 1655 (C = C). ^1^H-NMR (DMSO-*d*_*6*_) δ (ppm): 1.91 (s, 3 H, CH_3_), 6.60 (s, 1H_pyrrolone ring_), 7.42–8.08 (m, 11 H, Ar-H + 1H_olefinic_), 8.16 (d, 1H, Ar-H, *J* = 8.1 Hz), 10.69 (s, 1H, NH, exchangeable with D_2_O). ^13^C-NMR (DMSO-*d*_*6*_) δ (ppm): 20.3, 99.8, 123(2), 125(2), 126(3), 127.4, 128(5), 129(2), 130.5, 131(2), 133(3), 134(3), 142(2), 147.3, 160.7, 168.3, 169.0. Anal. calcd. for C_23_H_17_ClN_2_O_2_ (388.85): C, 71.04; H, 4.41; Cl, 9.12; N, 7.20. Found: C, 70.92; H, 4.32; Cl, 8.98; N, 7.28.

Method B: A solution of compound **16** (4 g, 10 mmol) in glacial acetic acid (20 mL) containing concentrated hydrochloric acid (1 mL) was heated under reflux for 30 min. The precipitated solid while heating was filtered off, dried and recrystallized from EtOH to give 14 as orange crystals; yield 72%.

### Biological evaluation

#### Insect rearing

A field population was collected from breeding sites in Abou Rawash, Giza, Egypt. The laboratory strain of *C. pipiens* larvae used in larvicidal activity was reared at the insectary of the Entomology Department, Faculty of Science, Ain Shams University, Cairo, Egypt, for several generations. This strain was reared in optimized conditions of temperature (27 ± 2 °C), relative humidity (75 ± 5%) and light and dark circadian (14 h: 10 h) according to Kufman, et al.,^66^ with some modifications. The adults were reared in a wooden cage (40 × 40 × 40 cm) for feeding, mating and oviposition so it was provided with deionized water-filled glass jars for adult emergence and then egg laying. Adults were fed on sugar solution (10%) and only females were fed on a blood meal from live pigeons for egg production. The egg rafts were transferred to enamel containers for larval hatching. The larvae were fed on a freshly prepared larval diet (biscuits dried: yeasts (3: 1))^67^. The pupae were collected in emergency jars daily.

#### Larvicidal activity

The potency of sixteen tested pyrrolone derivatives and chlorfenapyr as reference insecticide was assessed against the *C. pipiens* (third larval instar) following^68^ with some modifications. The stock solutions were prepared by dissolving the synthesized compounds in DMSO, while the chlorfenapyr (Challenger 24% EC) was obtained from BASF Chemicals Co. Egypt and dissolved in deionized water. Six concentrations were prepared in deionized water and Triton X-100 for synthesized compounds and in deionized water only for chlorfenapyr as follows (10, 25, 50, 100, 200 and 300 µg/mL) for lab strain, and (5, 10, 25, 50, 100 and 200 µg/mL) for field strain. The control was conducted by application of solvents only without exposure to tested compounds. Each concentration of the tested compounds, reference insecticide and control were replicated three times. A batch of 20 early third larval instar, was transferred to each prepared concentration (80 mL)^69^. The treatments and control were examined after 72 h and the mortality rates were estimated.

### Biochemical analysis

To evaluate the biochemical effects of pyrrolone derivatives, the 3rd instar of *C. pipiens* larvae was treated with LC_50_ of the six most effective pyrrolone derivatives for 72 h. The treated and untreated larvae were kept at freezing conditions at −20˚C. Then the larval bodies were homogenized in a chilled glass Teflon tissue grinder for 5 min, according to^70^. Homogenates were centrifuged at 8000 r.p.m in a refrigerated centrifuge for 15 min. The supernatants were stored at 5 ˚C until used for biochemical analysis^71^. For quantitative determination of the cytochrome P-450 monooxygenase activity, the p-nitro anisole o-demethylation was used according to the method of Hansen and Hodgson^72^ with some modifications.

### Statistical analysis

By using LdP-Line^©^ package software (Ehabsoft, Egypt), different lethal concentrations were determined at the 95% fiducial limits, in addition, slope ± standard error and *Chi-square test* were calculated for the goodness of fit according to **Finney**^73^. The relative potency was calculated to compare the effectiveness of tested compounds^74^. The biochemical analysis results were tested by one-way analysis of variance (ANOVA) by using the CoStat system for Windows, (Version 6.311, (CoHort software, Berkeley, CA 94701 https://www.cohortsoftware.com/costat.html*)*). The mean values were compared by Duncan’s multiple range test^75^ ANOVA statistics were significant (*P < 0.01*).

## Conclusion

A series of new pyrrolone and pyridazinone derivatives bearing naphthalene moiety were synthesized. The chemical structures of the synthesized compounds were assured by spectroscopic techniques. All these compounds were evaluated against field and laboratory strains of *Culex pipiens* larvae in comparison with reference pyrrole insecticide (chlorfenapyr). In the lab strain, the pyrrolone derivatives showed a great variation of activities, hence the compounds **17 > 9 > 15 > 10 > 16 > 5** were the most effective derivatives against *C. pipiens* larvae, which exhibited 6.77, 6.24, 5.84, 5.32, 4.44 and 3.74 folds than **11**, respectively. While in field strain, **17** > **15** > **9** > **10** > **16** > **2** showed toxicity 7.76, 7.11, 6.65, 6.04, 5.00 and 4.17 folds than **13**, respectively. The structure-activity relationship study illustrated the variations observed in the toxicity of synthesized compounds. The cytochrome P-450 monooxygenase activities were significantly increased in treated larvae of lab and field strains in comparison to untreated which is remarkably responsible for tested compounds activation. Pyrrolone derivatives are promising compounds with novel mode of action in pest management programs and more studies should be prepared to create more active compounds.

## Electronic supplementary material

Below is the link to the electronic supplementary material.

Supplementary Material 1

## Data Availability

All data generated or analyzed during this study are included in this published article and its supplementary information files.
