# Publisher Correction: Rational design and synthesis of new pyrrolone candidates as prospective insecticidal agents against *Culex pipiens* L. Larvae

**DOI:** 10.1038/s41598-024-79443-7

**Published:** 2024-12-03

**Authors:** Mohamed H. Hekal, Ahmed I. Hashem, Fatma S.M. Abu El-Azm, Doaa R. Abdel-Haleem, El-Hady Rafat, Yasmeen M. Ali

**Affiliations:** 1 Department of Chemistry, Faculty of Science, Ain Shams University, Abbassia, Cairo, 11566 Egypt; 2 Entomology Department, Faculty of Science, Ain Shams University, Abbassia, Cairo, 11566 Egypt

Correction to: *Scientific Reports* 10.1038/s41598-024-74011-5, published online 18 October 2024

In the original version of the Article, Figure 6 was a duplication of Figure 5.

The original Article has been corrected.

