# Supplementary material for: Rational design and synthesis of new pyrrolone candidates as prospective insecticidal agents against Culex pipiens L. Larvae
Source: Sci Rep. 2024 Oct 18;14:24467. doi: 10.1038/s41598-024-74011-5 (PMC11489436; doi:10.1038/s41598-024-74011-5)
Supplement: Supplementary file 1 — Supplementary Material 1 [file 41598_2024_74011_MOESM1_ESM.pdf]

SUPPLEMENTARY INFORMATION

Rational design and synthesis of new pyrrolone candidates as prospective insecticidal agents against *Culex pipiens* L. Larvae

Mohamed H. Hekal[1]*, Ahmed I. Hashem[1], Fatma S.M. Abu El-Azm[1]*, Doaa R. Abdel-Haleem[2], El-Hady Rafat[1], Yasmeen M. Ali[1]

[1] Department of Chemistry, Faculty of Science, Ain Shams University, Abbassia 11566, Cairo, Egypt

[2] Entomology Department, Faculty of Science, Ain Shams University, Abbassia 11566, Cairo, Egypt.

Corresponding authors;

* Mohamed H. Hekal

Email: mohamed.hekal@sci.asu.edu.eg, mohahekal2007@yahoo.com

* Fatma S.M. Abu El-Azm

Email: fatma.abuelazm@sci.asu.edu.eg, ftmsaber@yahoo.com

CONTENTS

Copies of IR, ^{1}H NMR and ^{13}C NMR spectra of the products

Figure Legends:

Figure 1S. Compound **2** IR

Figure 2S. Compound **2** ^{1}H NMR

Figure 3S. Compound **3** IR

Figure 4S. Compound **3** ^{1}H NMR

Figure 5S. Compound **3** ^{1}H NMR (DMSO-d_6 + D_2O)

Figure 6S.Compound **3** ^{13}C NMR

Figure 7S. Compound **4** IR

Figure 8S. Compound **4** ^{1}H NMR

Figure 9S. Compound **4** ^{1}H NMR (DMSO-d_6 + D_2O)

Figure 10S.Compound **4** ^{13}C NMR

Figure 11S. Compound **5** IR

Figure 12S. Compound **5** ^{1}H NMR

Figure 13S. Compound **5** ^{1}H NMR (DMSO-d_6 + D_2O)

Figure 14S.Compound **5** ^{13}C NMR

Figure 15S. Compound **6** IR

Figure 16S. Compound **6** ^{1}H NMR

Figure 17S.Compound **6** ^{13}C NMR

Figure 18S. Compound **7** IR

Figure 19S. Compound **7** ^{1}H NMR

Figure 20S.Compound **7** ^{13}C NMR

Figure 21S. Compound **8** IR

Figure 22S. Compound **8** ^{1}H NMR

Figure 23S. Compound **8** ^{1}H NMR (DMSO-d_6 + D_2O)

Figure 24S.Compound **8** ^{13}C NMR

Figure 25S. Compound **9** IR

Figure 26S. Compound **9** ^{1}H NMR

Figure 27S. Compound **9** ^{1}H NMR (DMSO-d_6 + D_2O)

Figure 28S.Compound **9** ^{13}C NMR

Figure 29S. Compound **10** IR

Figure 30S. Compound **10** ^{1}H NMR

Figure 31S. Compound **10** ^{1}H NMR (DMSO-d_6 + D_2O)

Figure 32S.Compound **10** ^{13}C NMR

Figure 33S. Compound **11** IR

Figure 34S. Compound **11** ^{1}H NMR

Figure 35S. Compound **11** ^{1}H NMR (DMSO-d_6 + D_2O)

Figure 36S.Compound **11** ^{13}C NMR

Figure 37S. Compound **12** IR

Figure 38S. Compound **12** ^{1}H NMR

Figure 39S. Compound **12** ^{1}H NMR (DMSO-d_6 + D_2O)

Figure 40S.Compound **12** ^{13}C NMR

Figure 41S. Compound 1**3** IR

Figure 42S. Compound 1**3** ^{1}H NMR

Figure 43S. Compound 1**3** ^{1}H NMR (DMSO-d_6 + D_2O)

Figure 44S.Compound 1**3** ^{13}C NMR

Figure 45S. Compound **14** IR

Figure 46S. Compound **14** ^{1}H NMR

Figure 47S. Compound **15** IR

Figure 48S. Compound **15** ^{1}H NMR

Figure 49S. Compound **15** ^{1}H NMR (DMSO-d_6 + D_2O)

Figure 50S.Compound **15** ^{13}C NMR

Figure 51S. Compound **16** IR

Figure 52S. Compound **16** ^{1}H NMR

Figure 53S. Compound **16** ^{1}H NMR (DMSO-d_6 + D_2O)

Figure 54S.Compound **16** ^{13}C NMR

Figure 55S. Compound **17** IR

Figure 56S. Compound **17** ^{1}H NMR

Figure 57S. Compound **17** ^{1}H NMR (DMSO-d_6 + D_2O)

Figure 58S.Compound **17** ^{13}C NMR

Copies of IR, ^{1}H NMR and ^{13}C NMR spectra of the products

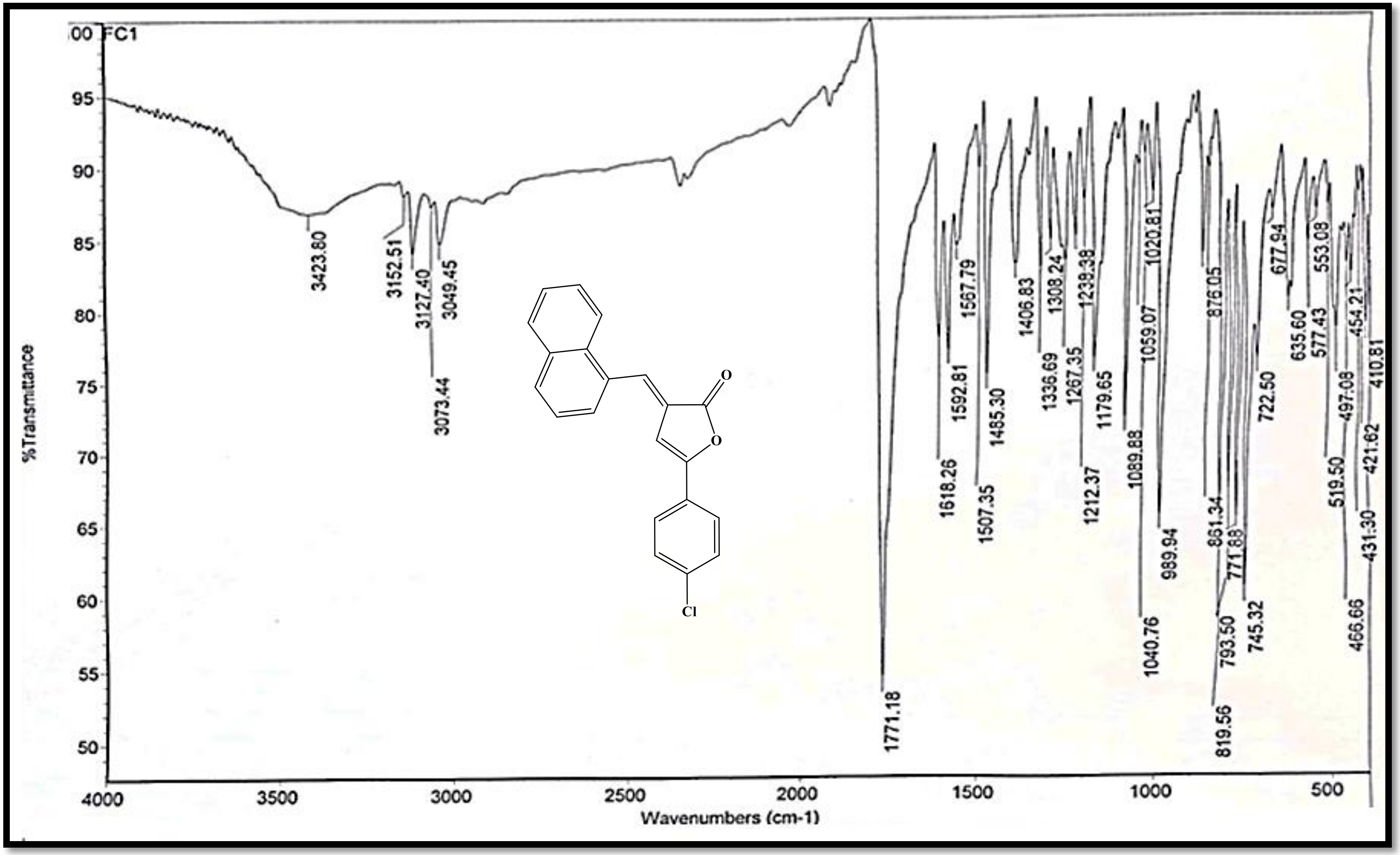

Figure 1S. IR spectrum of compound (2)

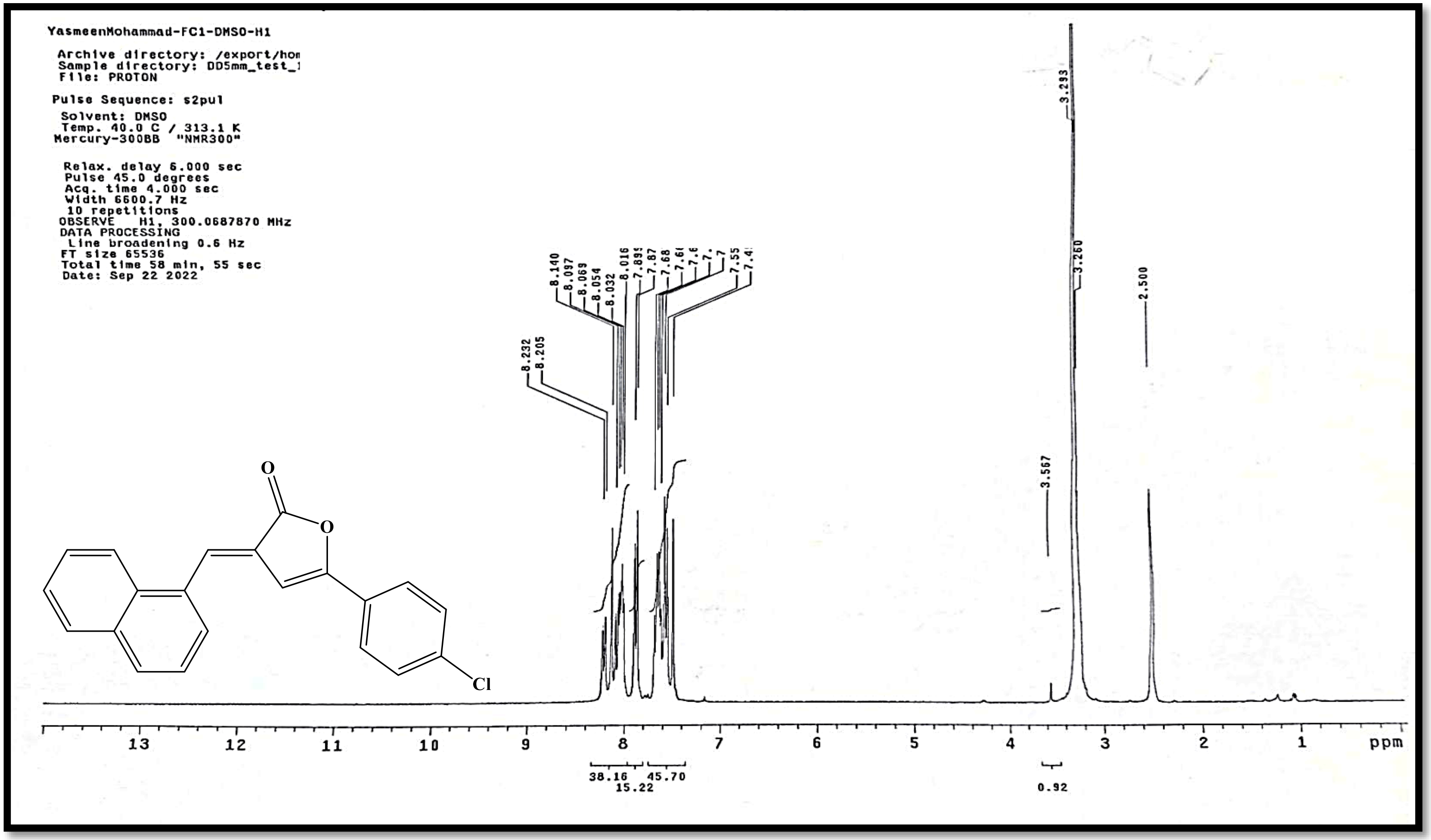

Figure 2S. ^{1}H-NMR (DMSO- d_6) of compound (2)

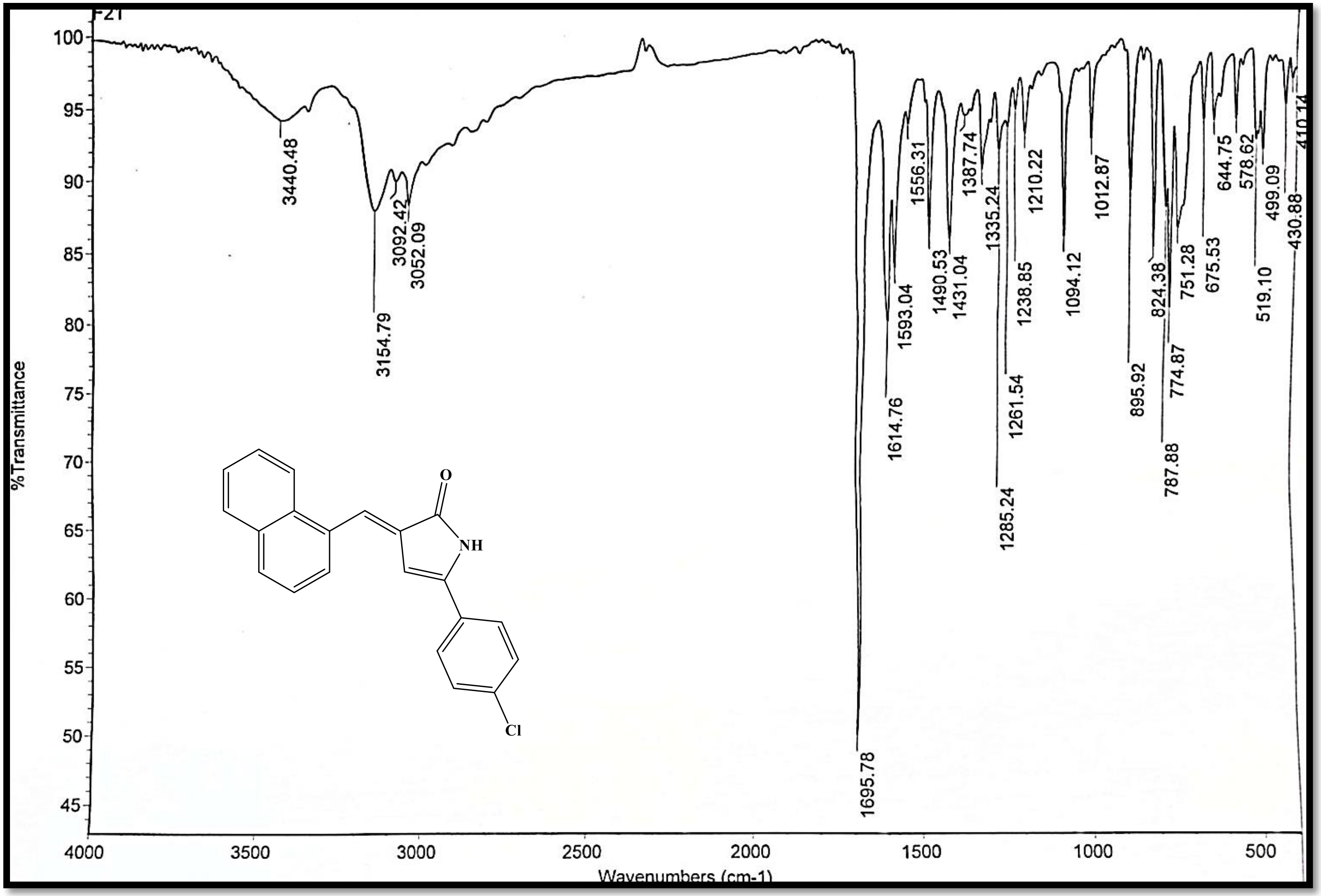

Figure 3S. IR spectrum of compound (3)

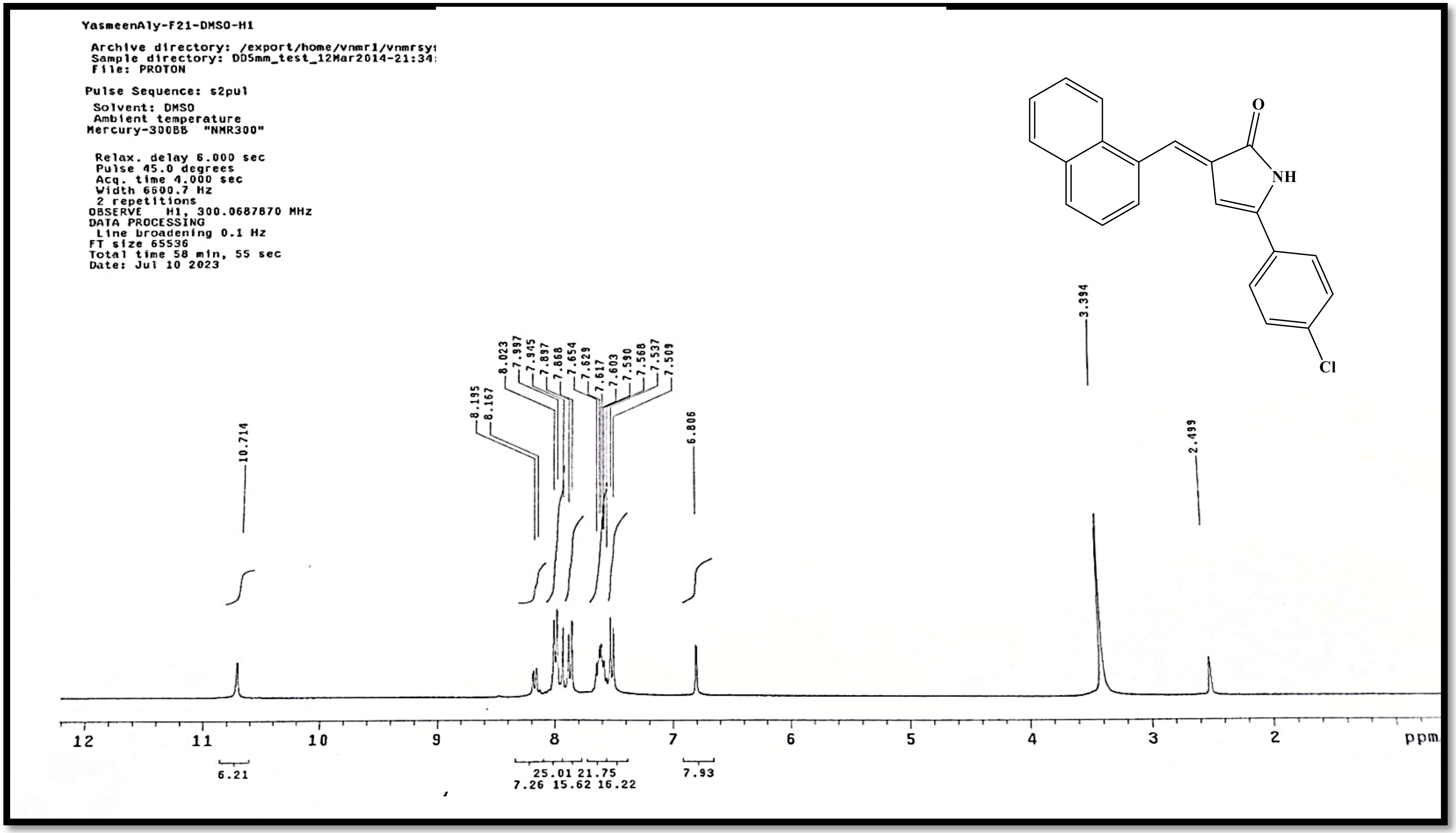

Figure 4S. ^{1}**H-NMR (DMSO- d**$_{6}$**) of compound (3)**

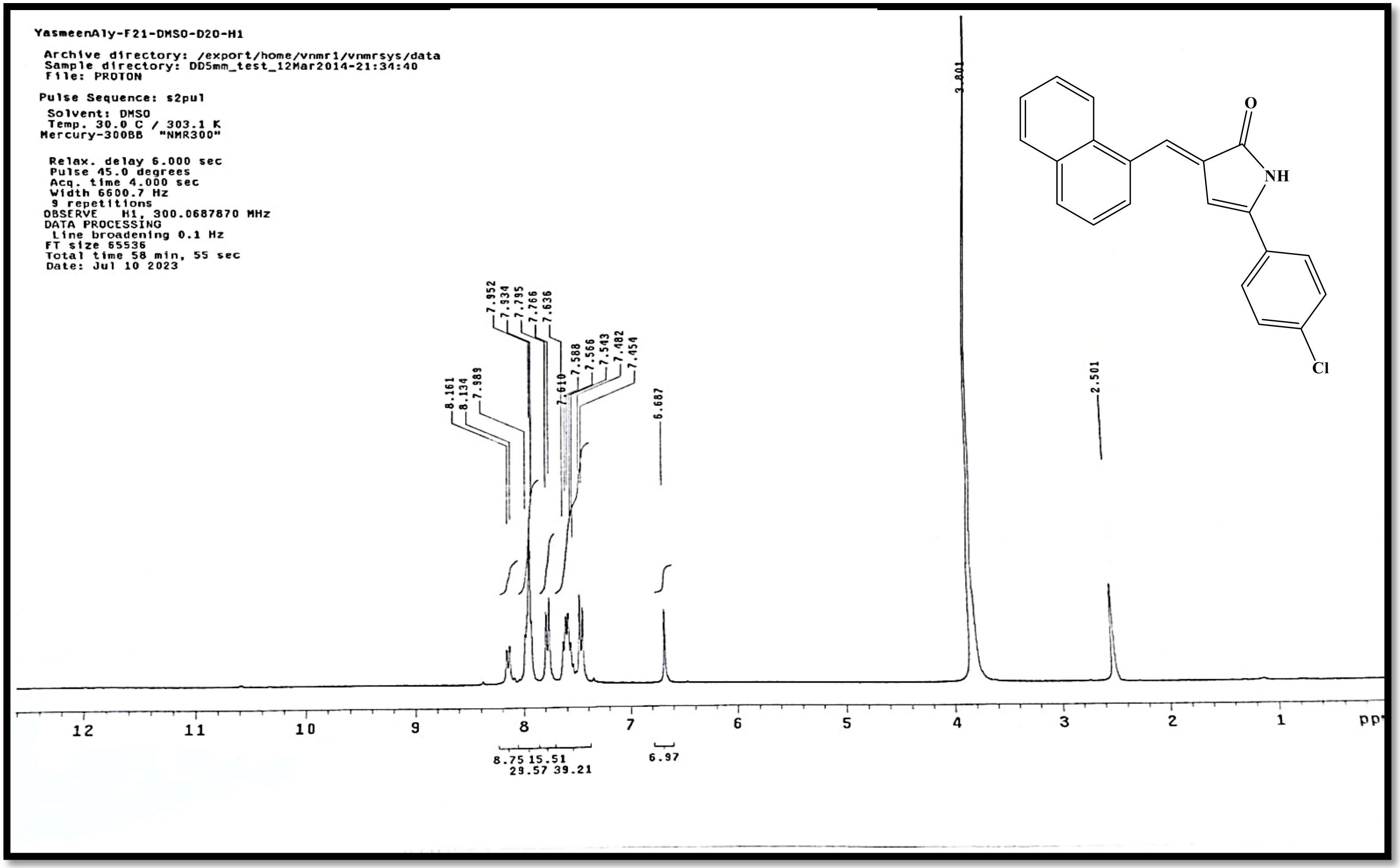

Figure 5S. ^{1}H-NMR spectrum (DMSO-d_6 + D_2O) of compound (3)

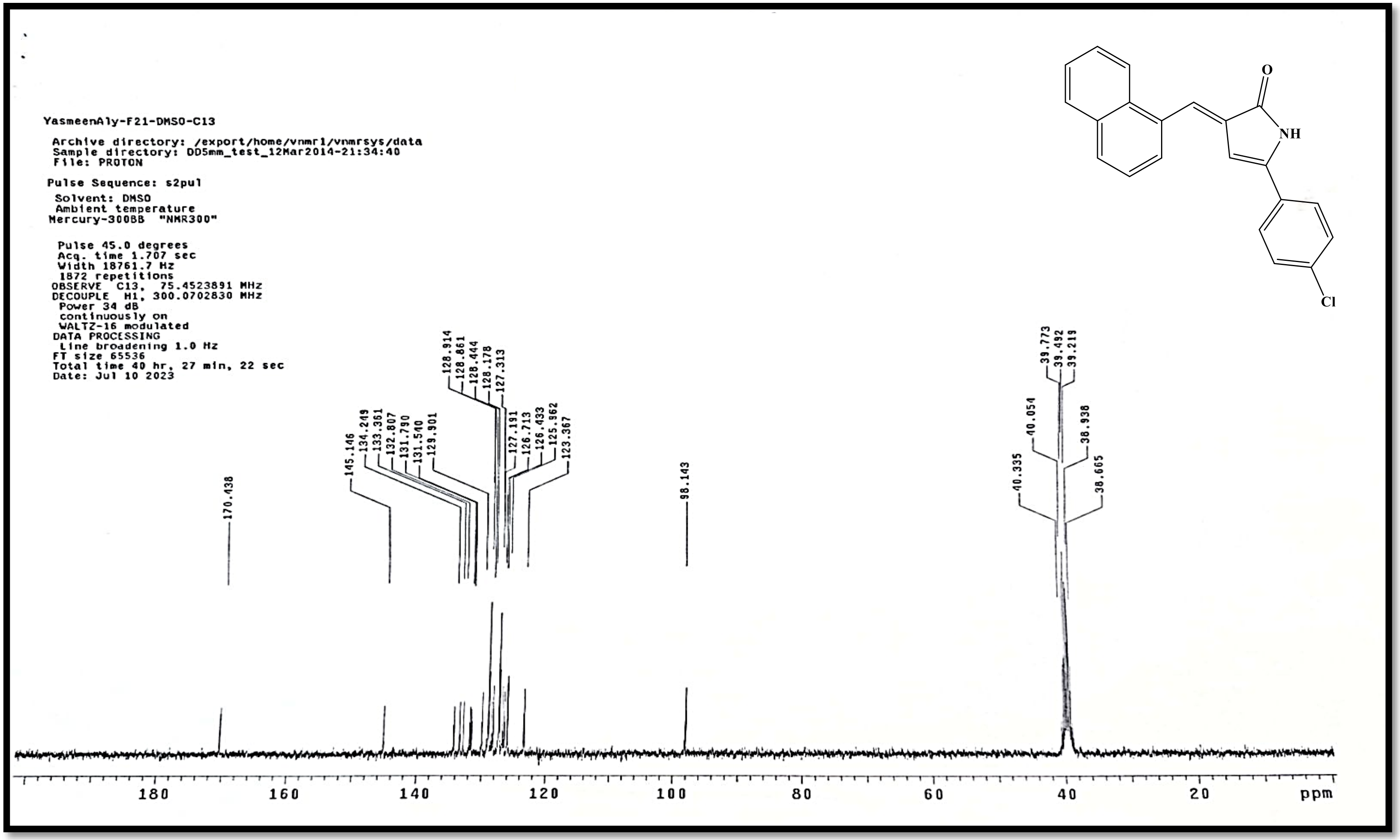

Figure 6S. ^{13}**C-NMR spectrum (DMSO-d$_6$) of Compound (3)**

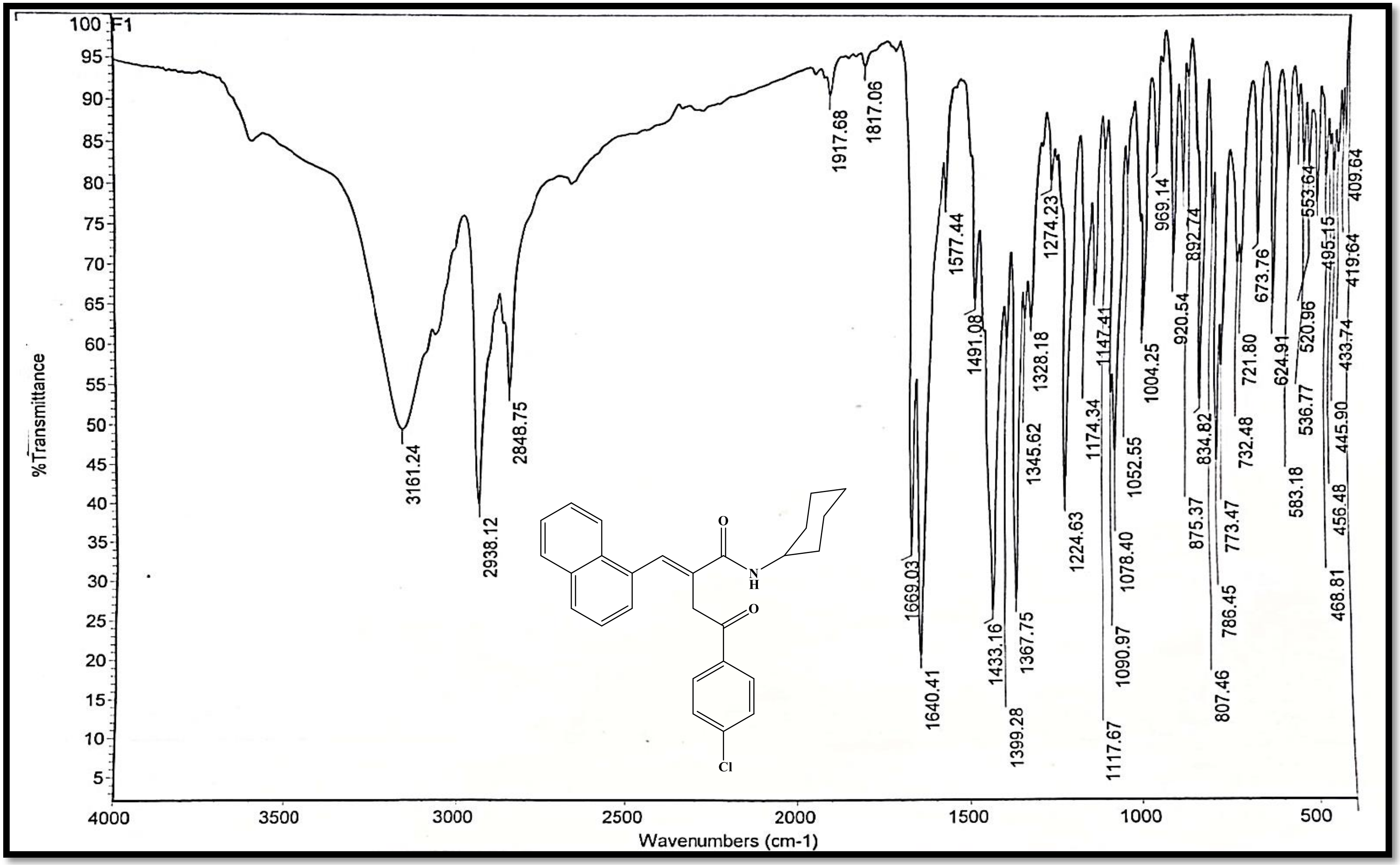

Figure 7S. IR spectrum of compound (4)

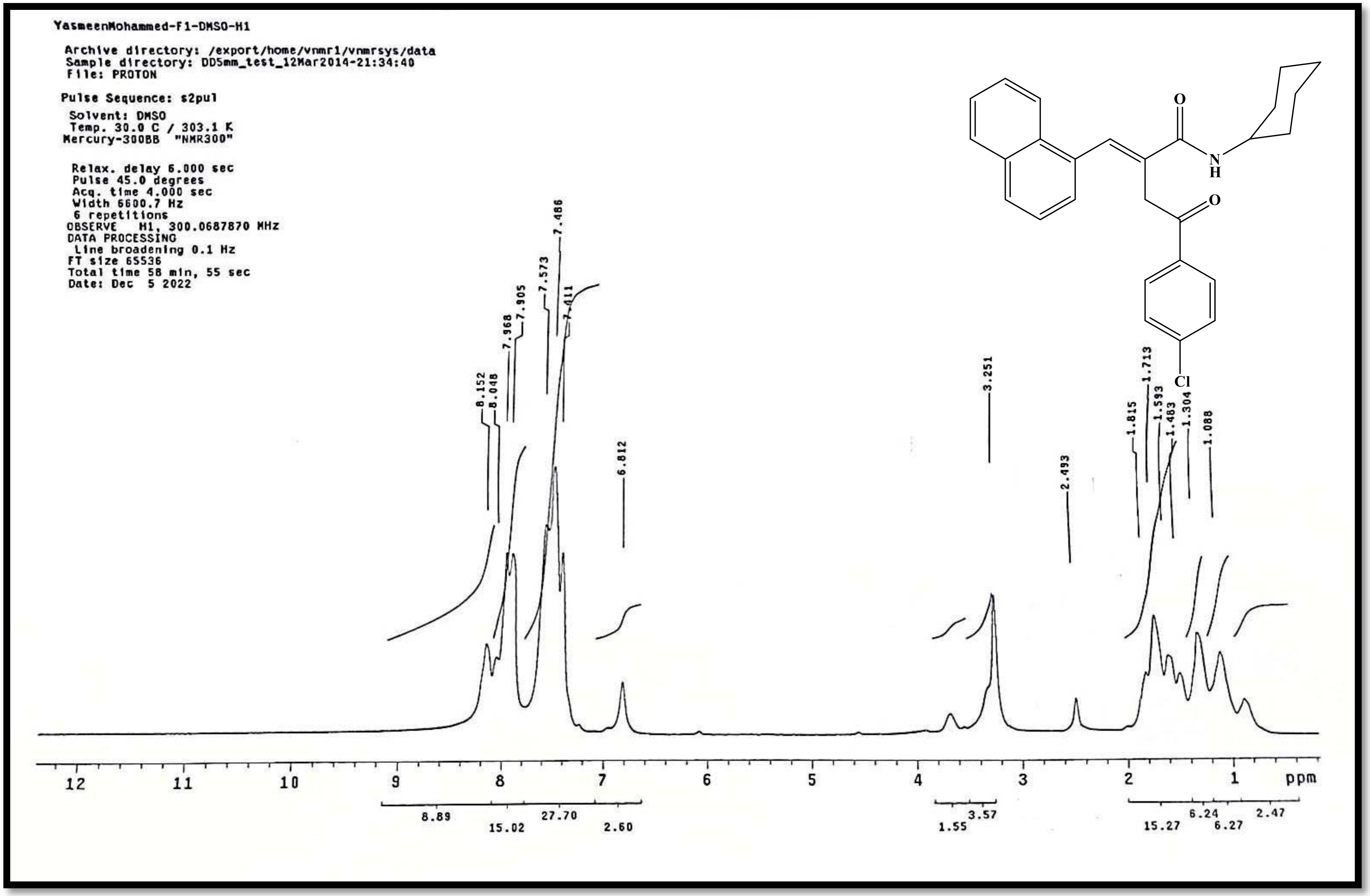

Figure 8S. ^{1}H-NMR (DMSO- d_6) of compound (4)

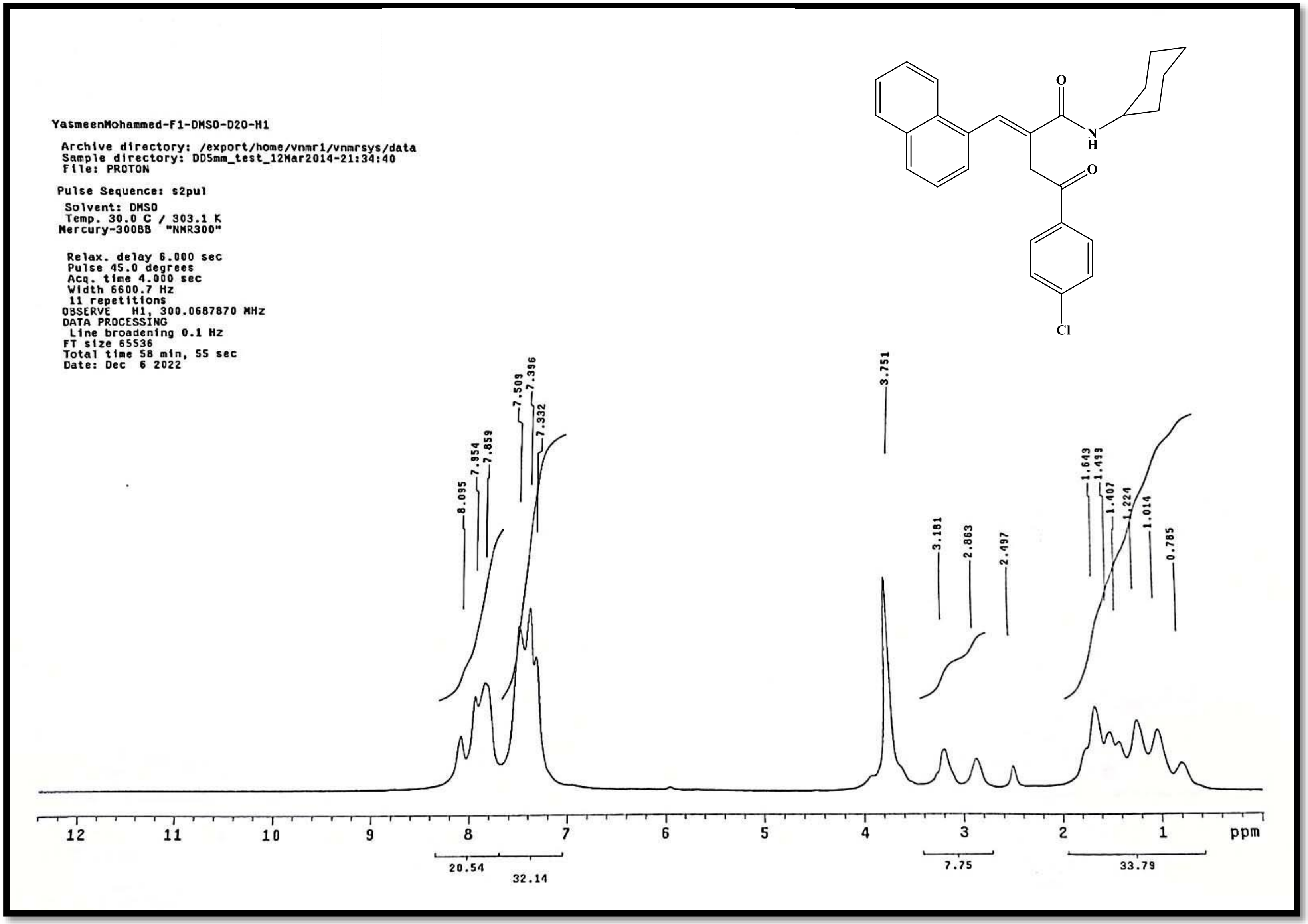

Figure 9S. ^{1}H-NMR spectrum (DMSO-d_6 + D_2O) of Compound (4)

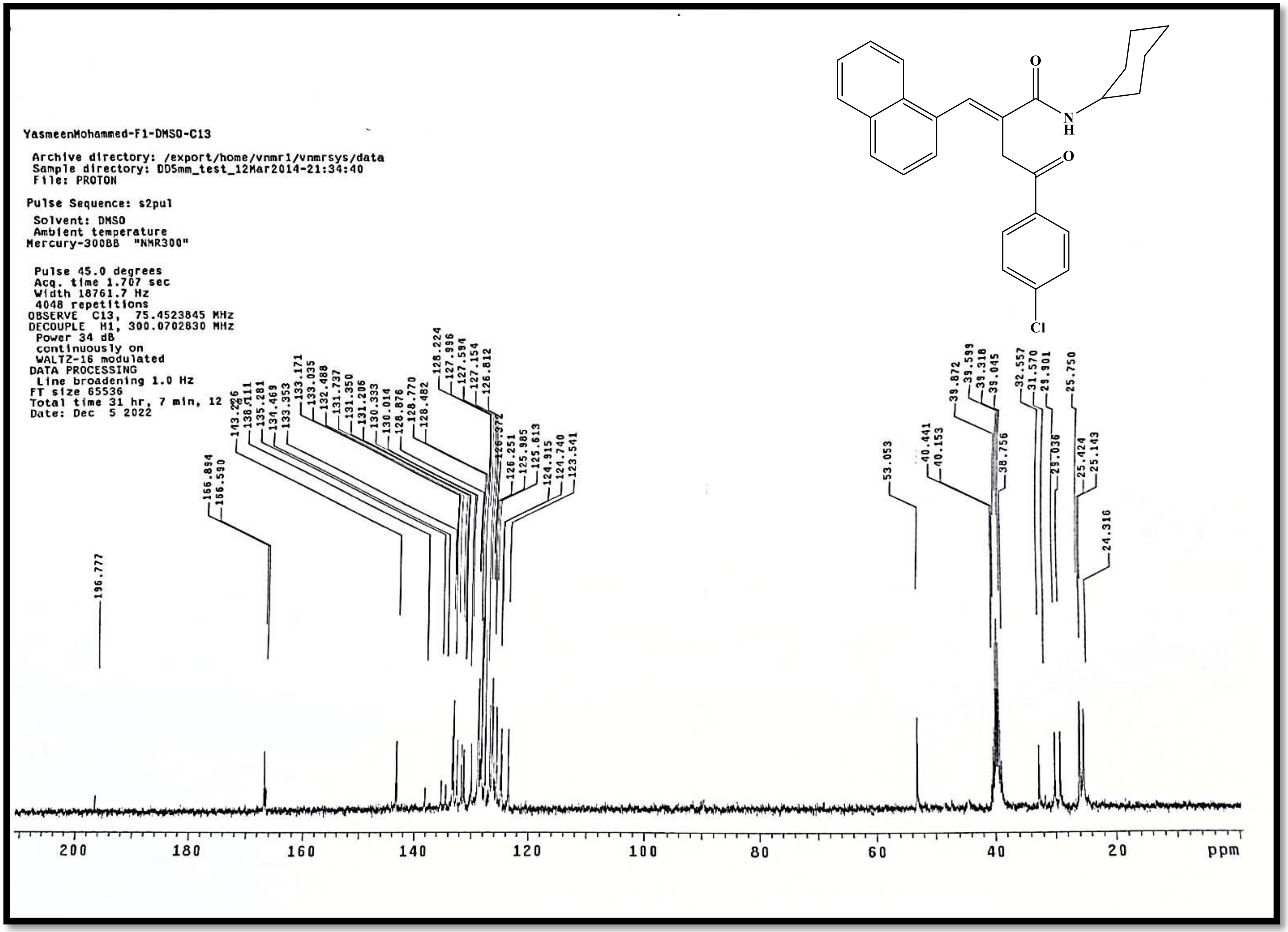

Figure 10S. ^{13}**C-NMR spectrum (DMSO-d**$_6$**) of compound (4)**

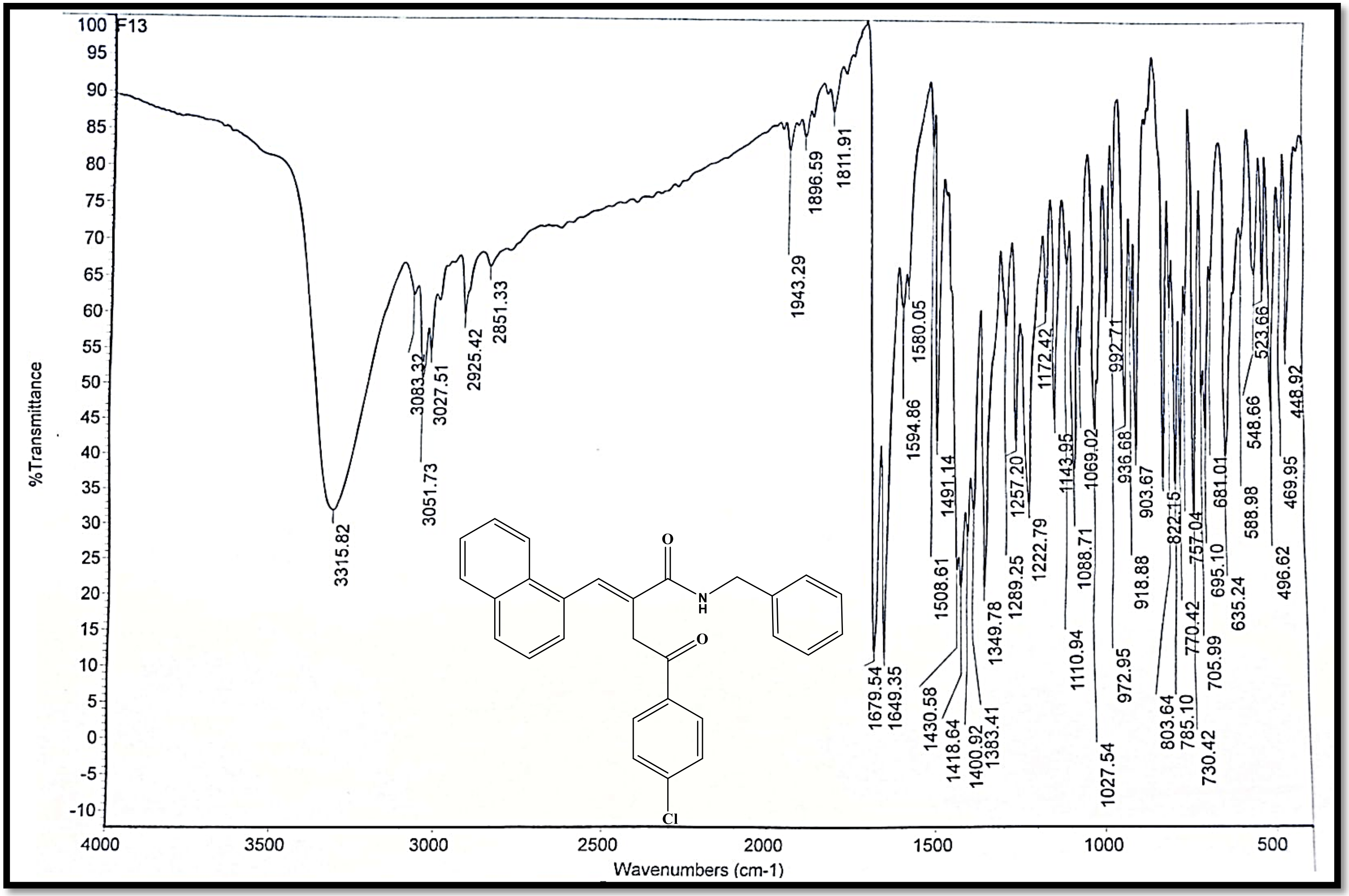

Figure 11S. IR spectrum of compound (5)

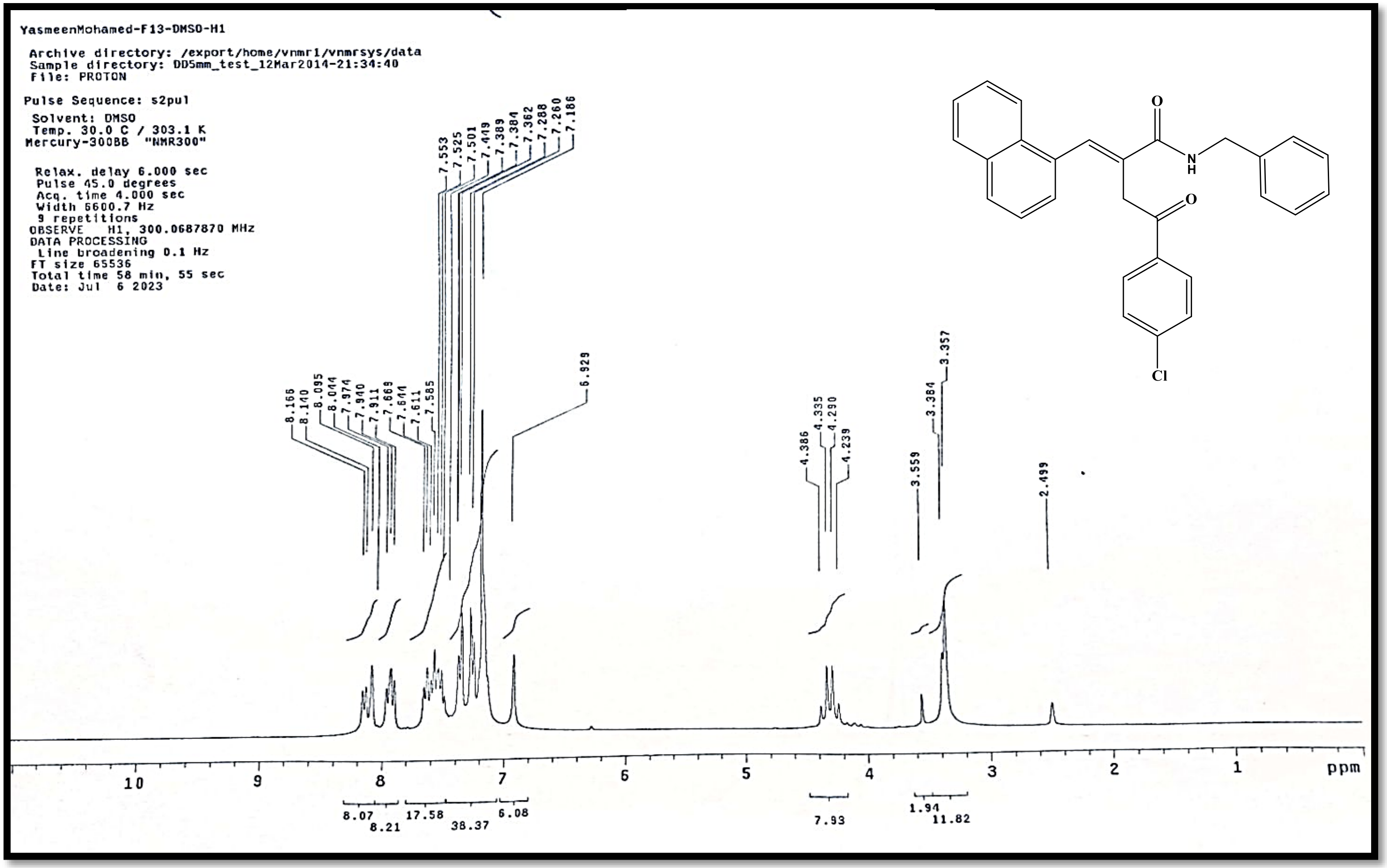

Figure 12S. ^{1}H-NMR (DMSO- d_6) of compound (5)

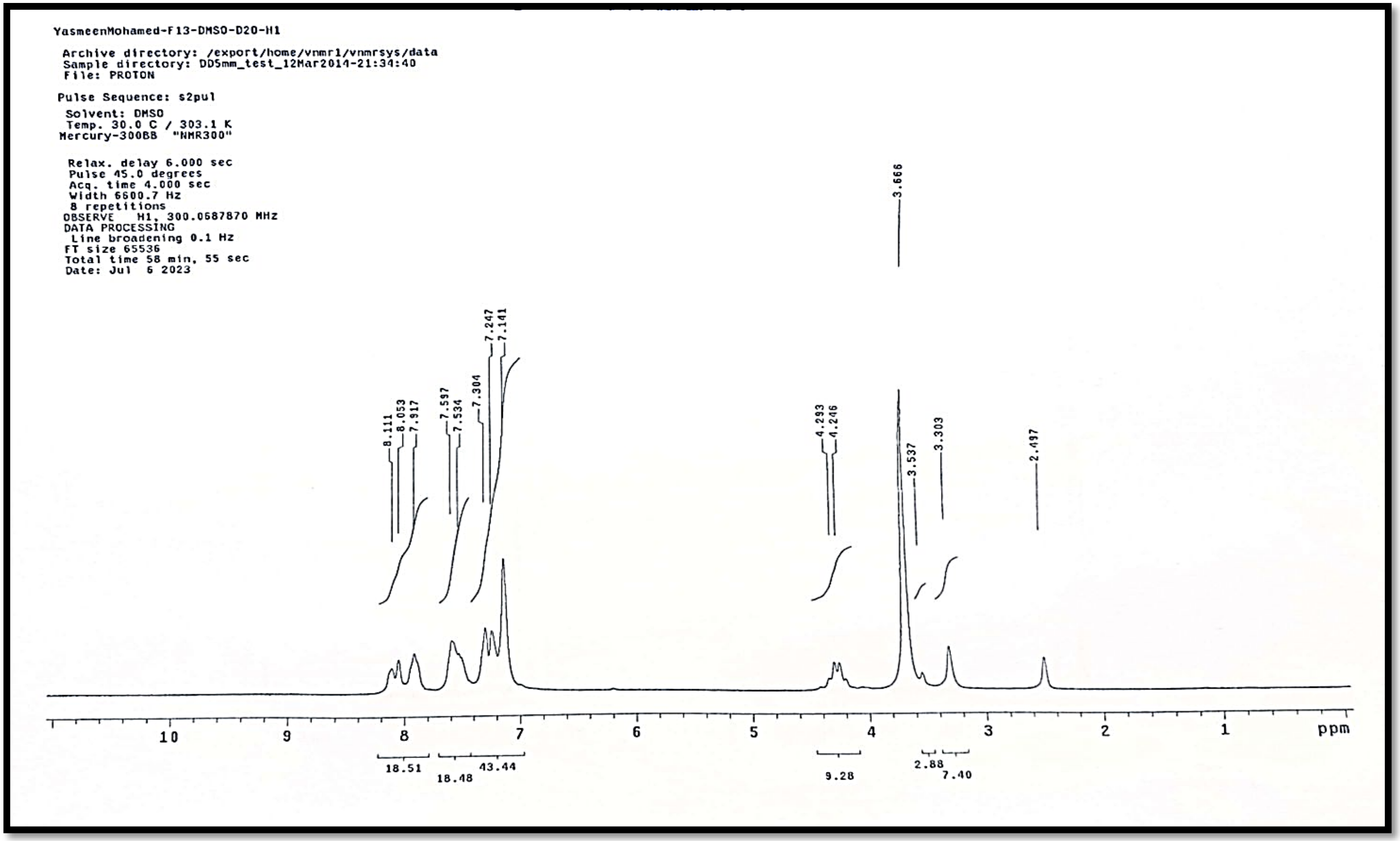

Figure 13S. ^{1}H-NMR spectrum (DMSO-d_6 + D_2O) of compound (5)

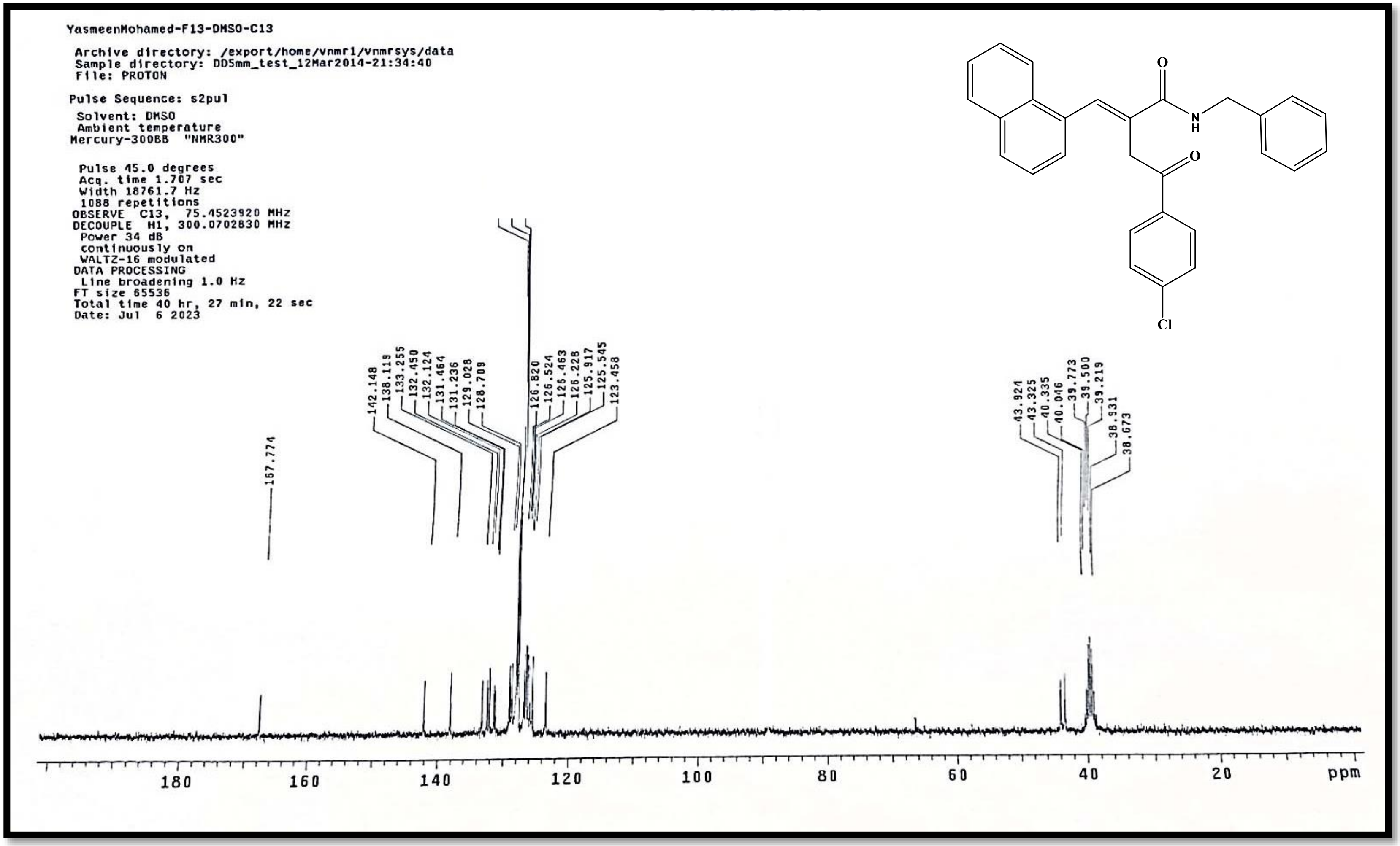

Figure 14S. ^{13}**C-NMR spectrum (DMSO-d**$_6$**) of compound (5)**

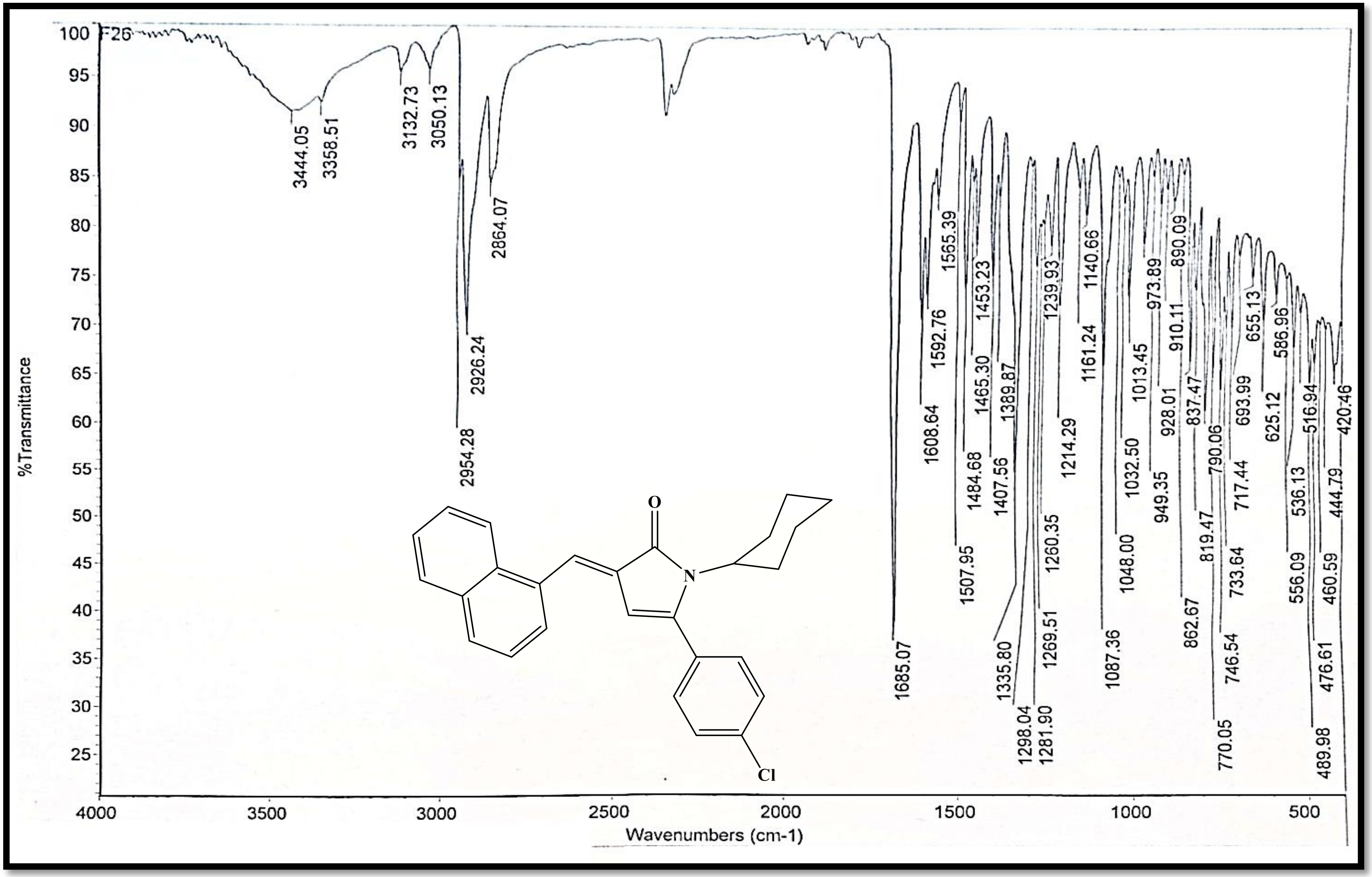

Figure 15S. IR spectrum of compound (6)

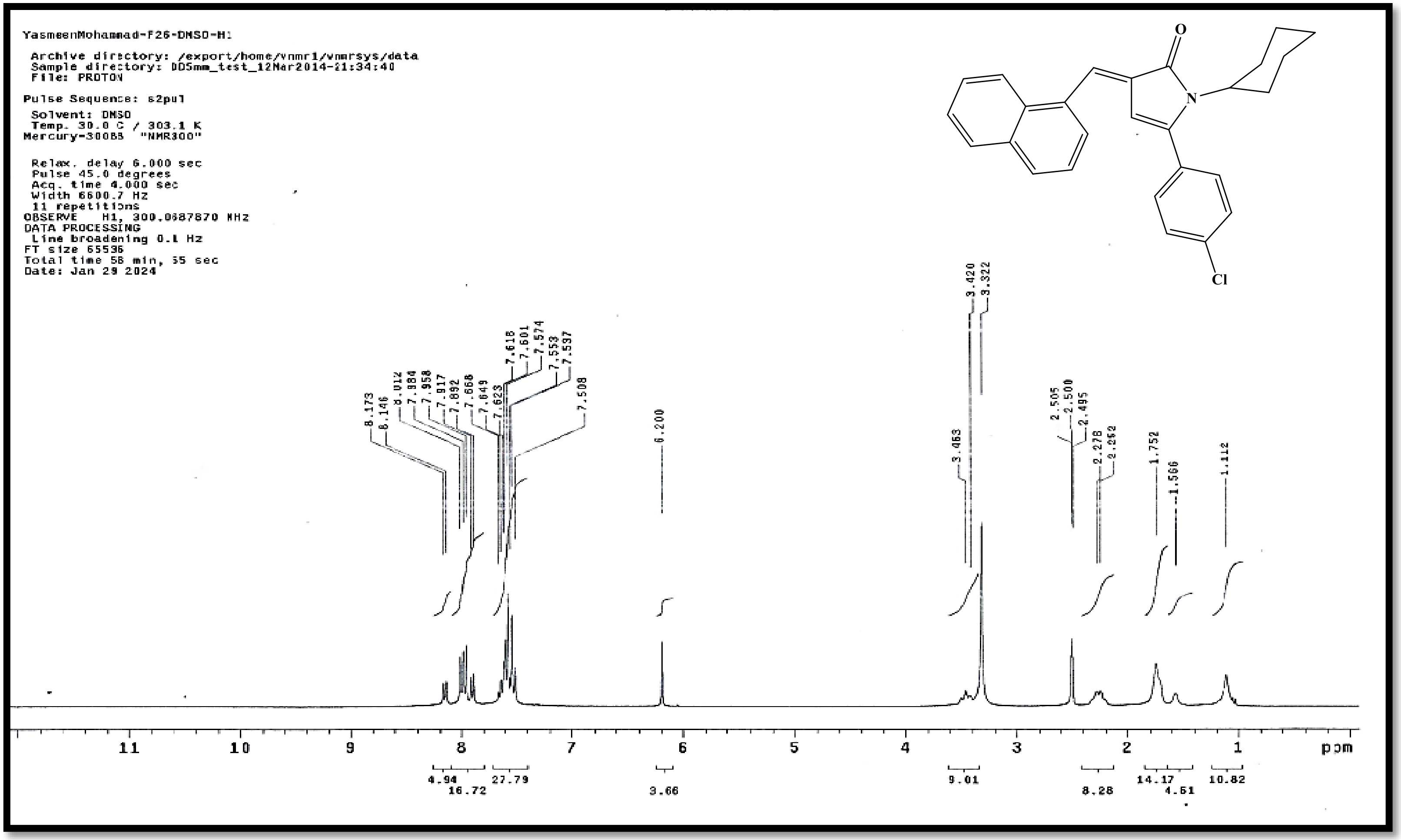

Figure 16S. ^{1}H-NMR (DMSO- d_6) of compound (6)

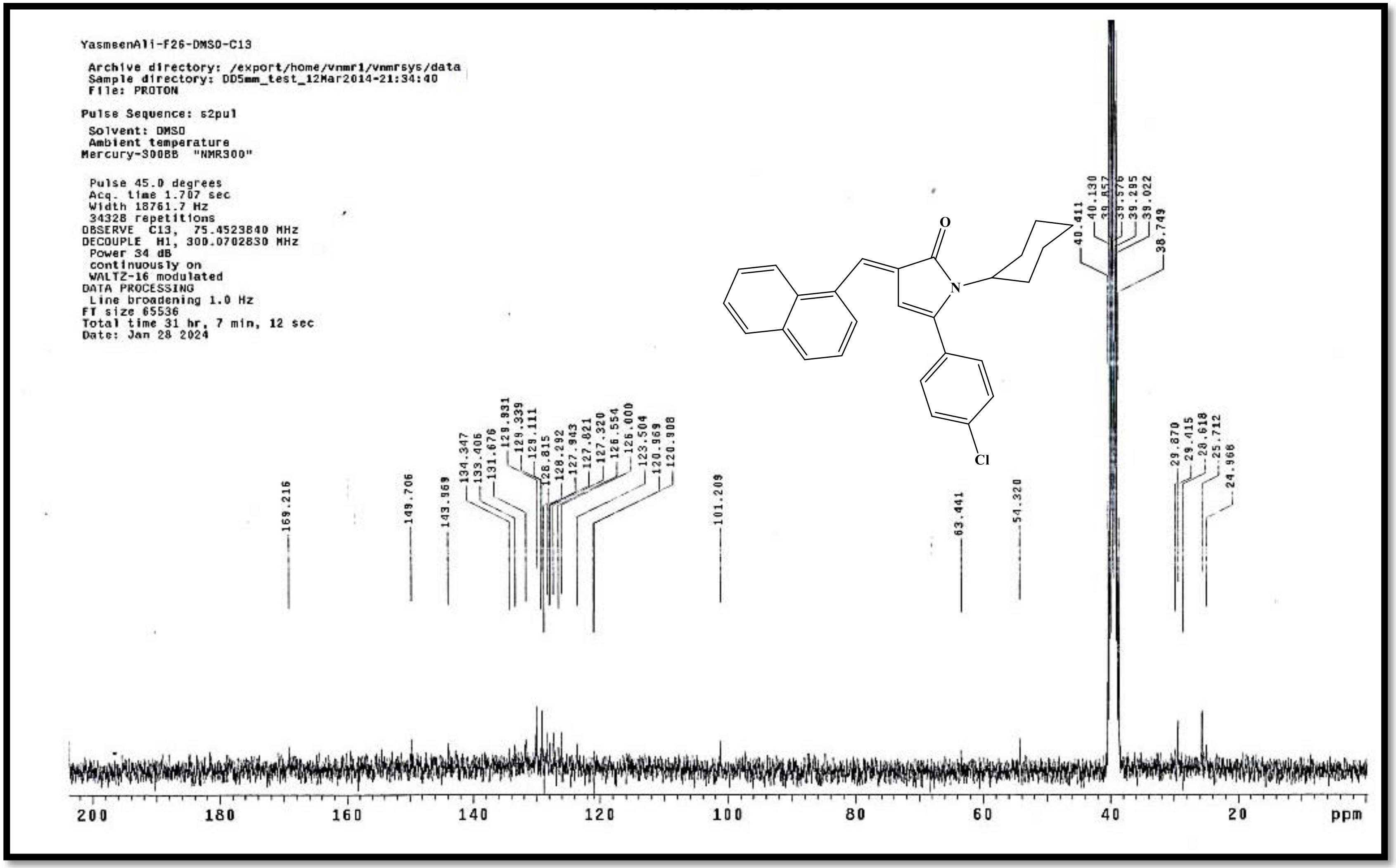

Figure 17S. ^{13}**C-NMR spectrum (DMSO-d$_6$) of compound (6)**

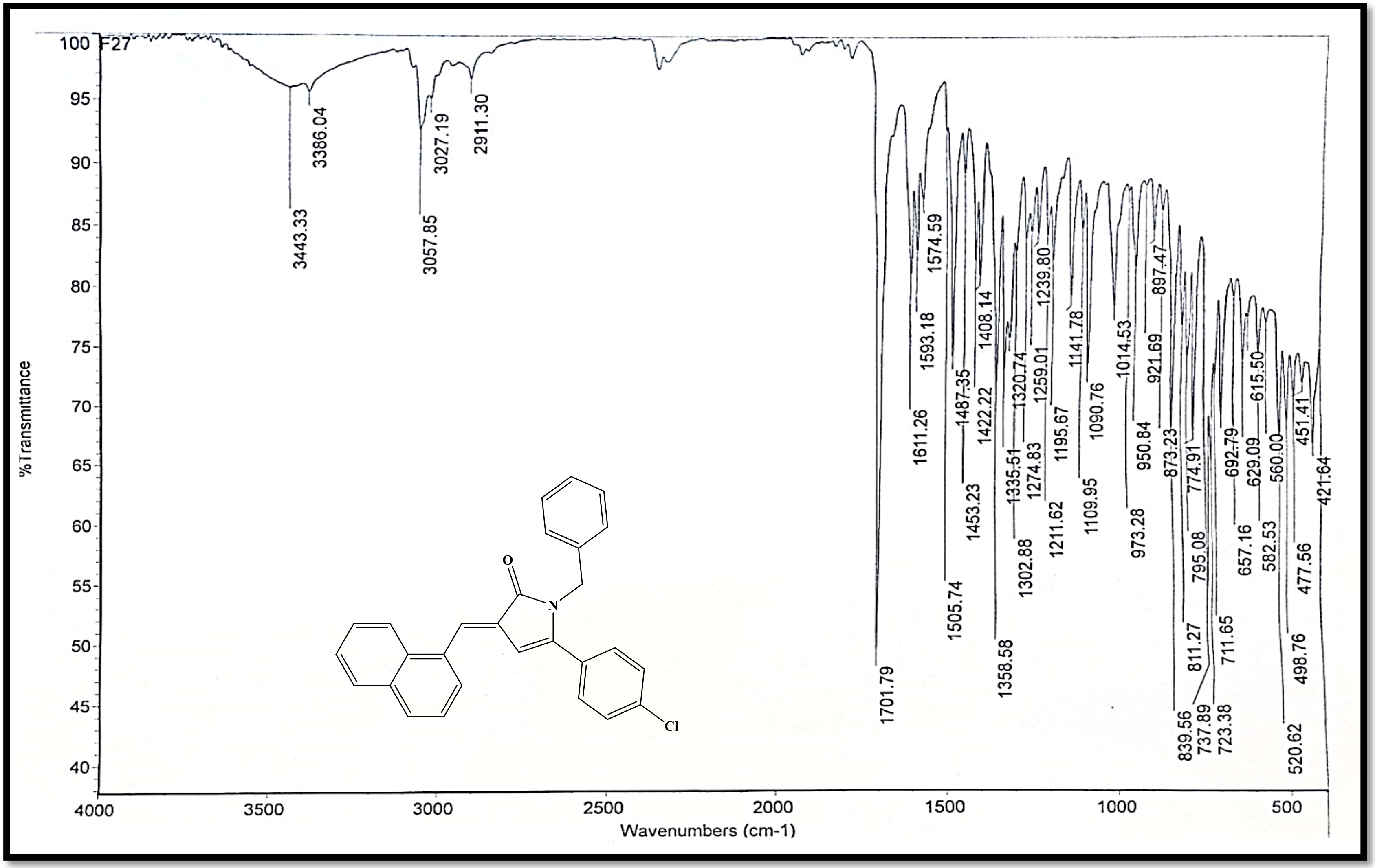

Figure 18S. IR spectrum of compound (7)

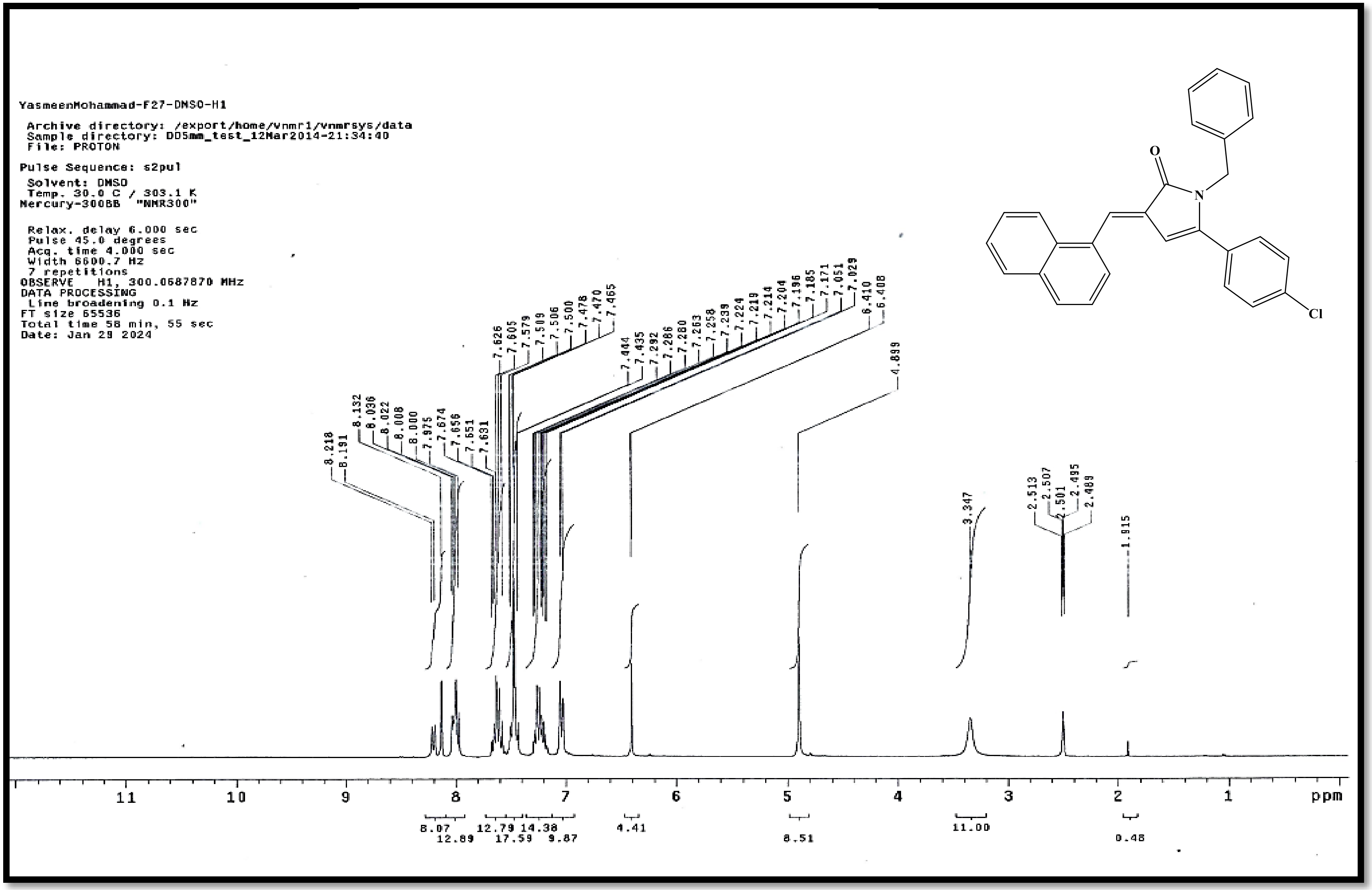

Figure 19S. ^{1}**H-NMR (DMSO-** d_6**) of compound (7)**

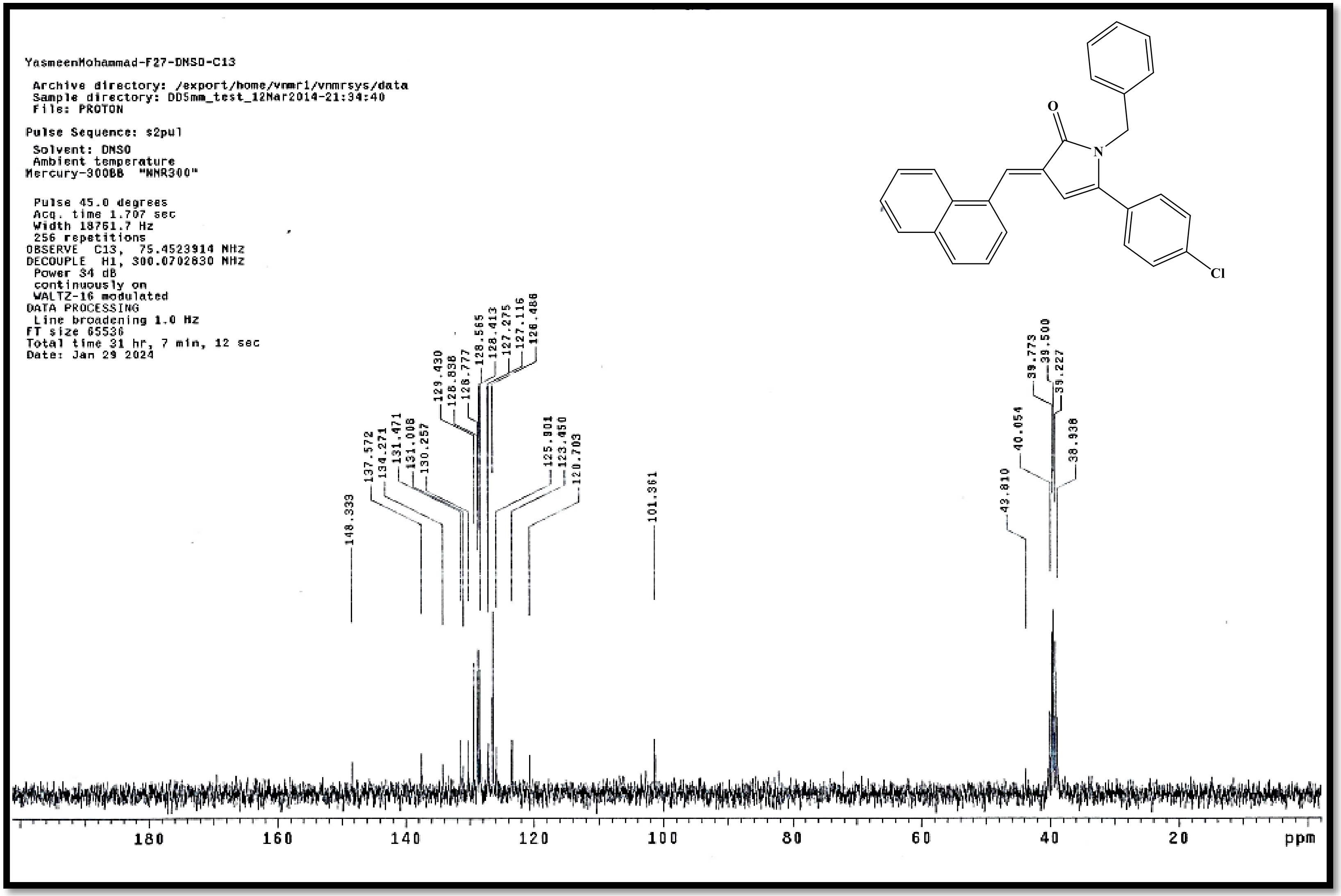

Figure 20S. ^{13}**C-NMR spectrum (DMSO-d$_6$) of compound (7)**

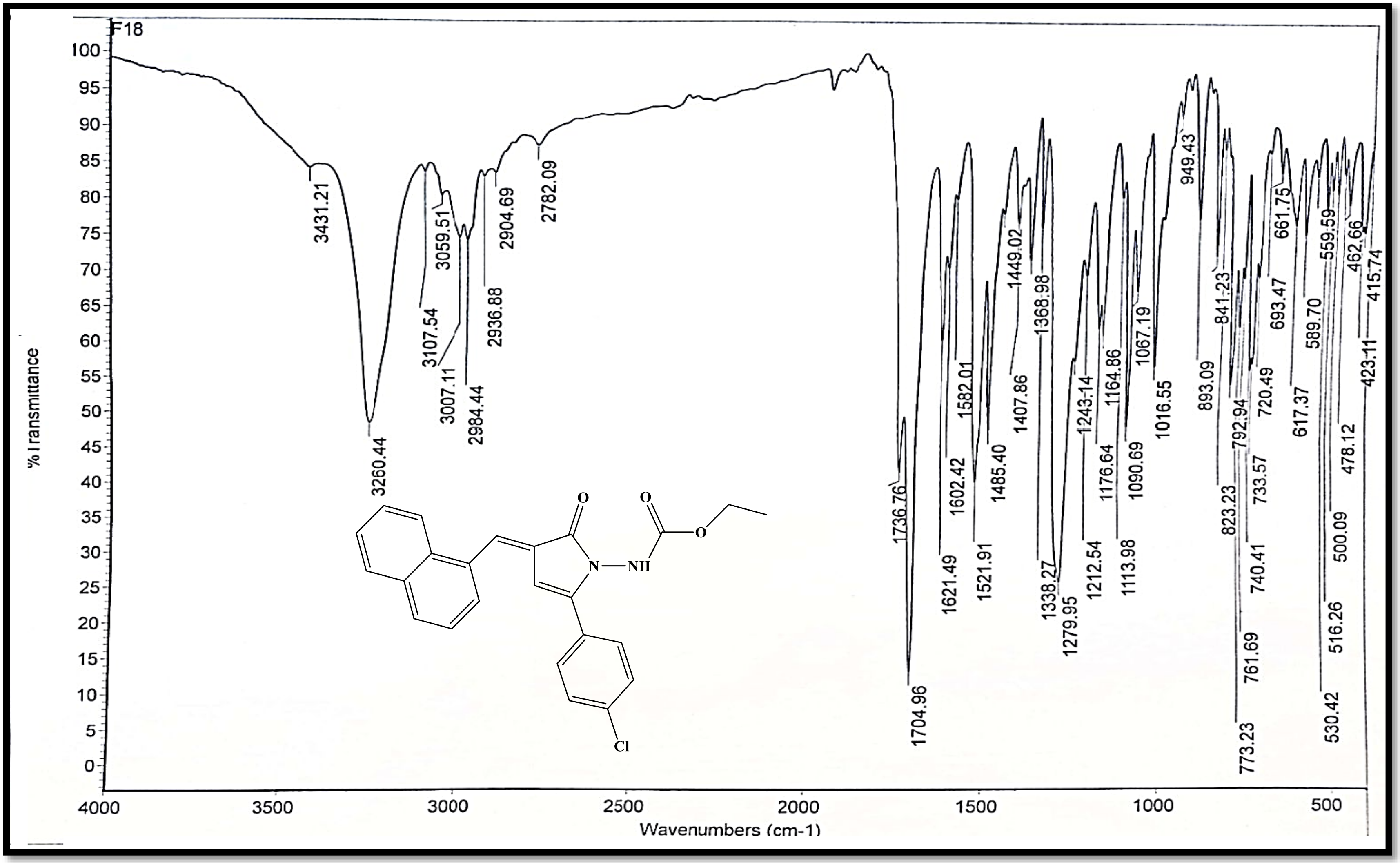

Figure 21S. IR spectrum of compound (8)

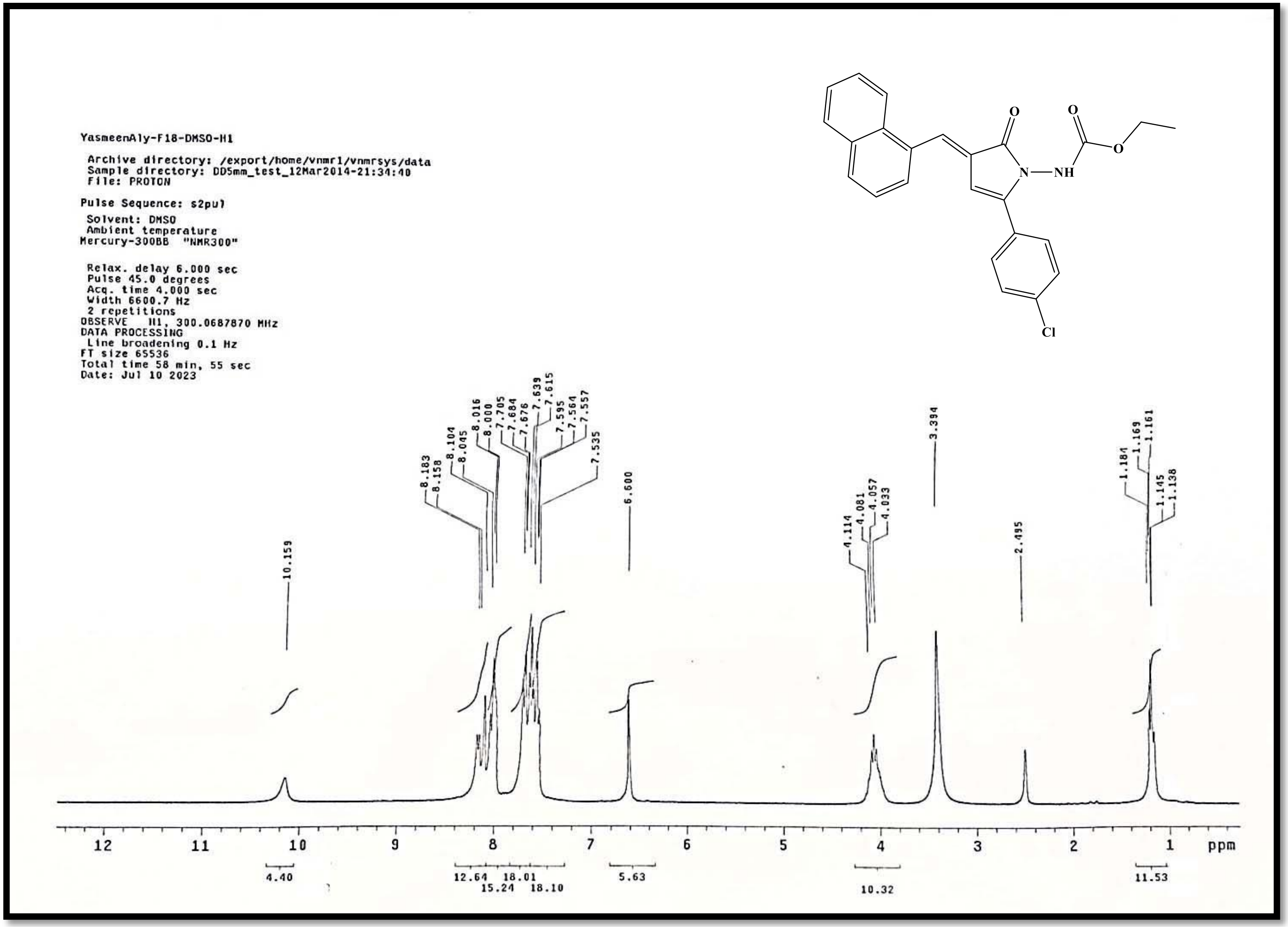

Figure 22S. ^{1}H-NMR (DMSO- d_6) of compound (8)

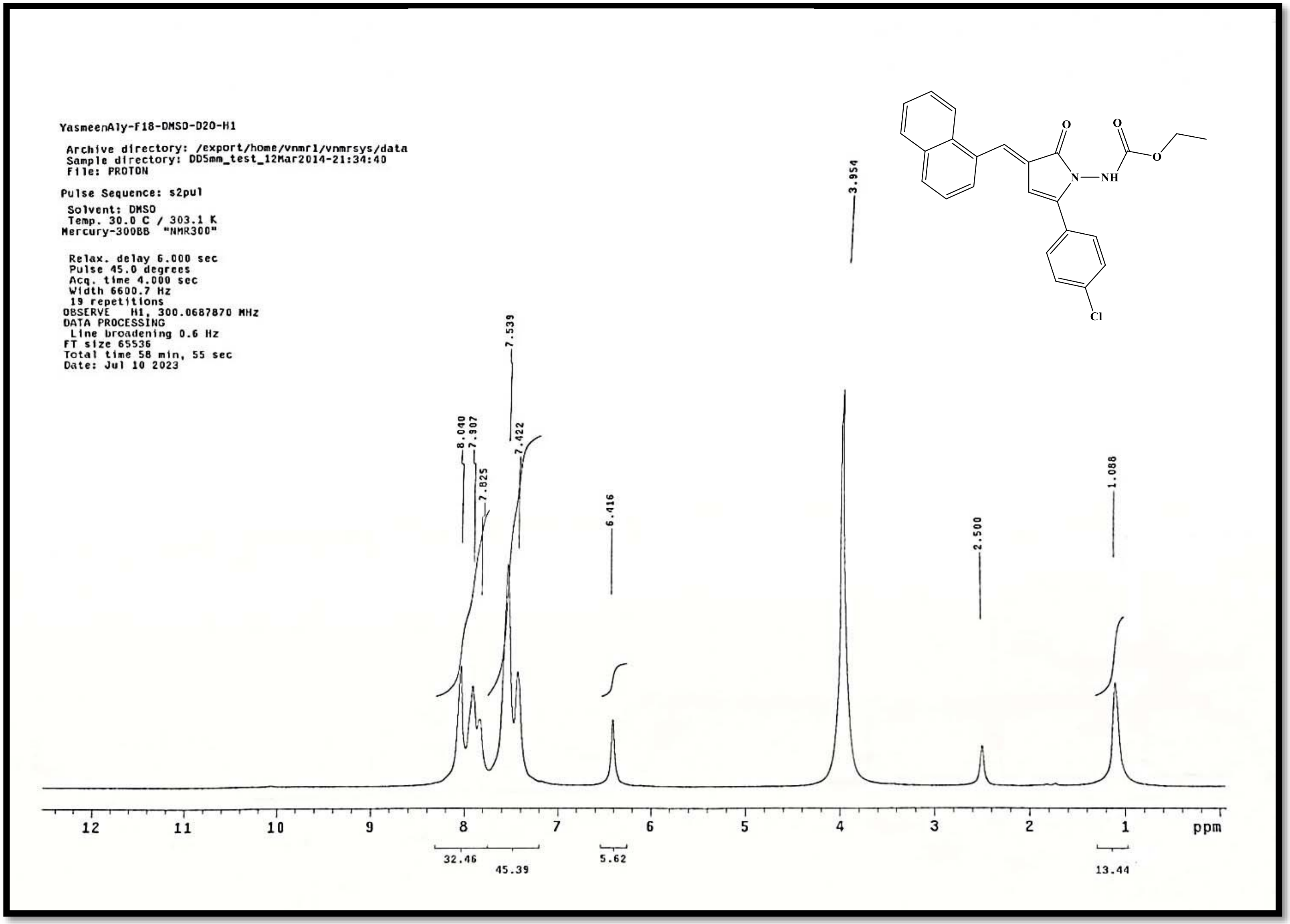

Figure 23S. ^{1}H-NMR spectrum (DMSO-d_6 + D_2O) of compound (8)

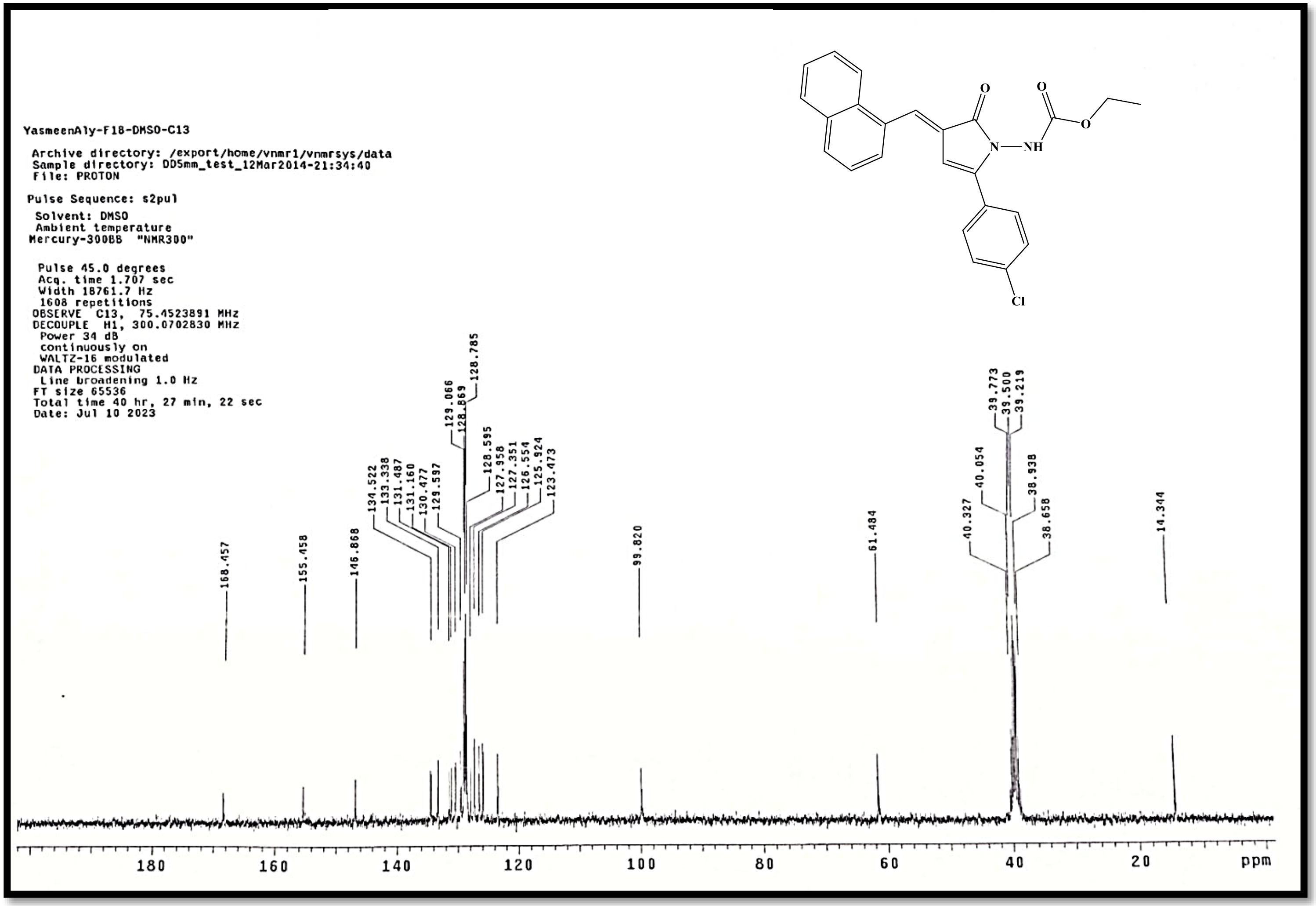

Figure 24S. ^{13}**C-NMR spectrum (DMSO-d$_6$) of Compound (8)**

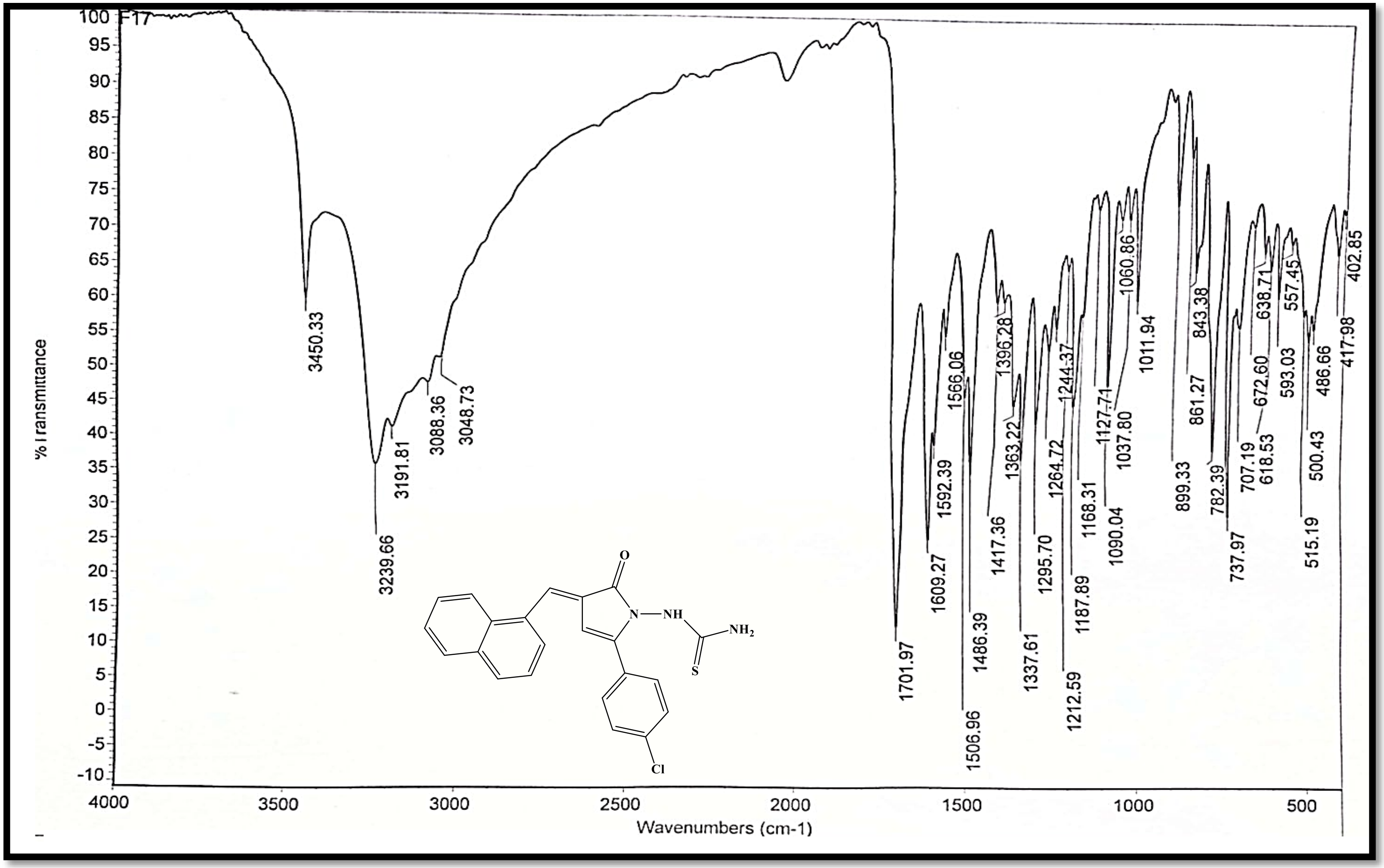

Figure 25S. IR spectrum of compound (9)

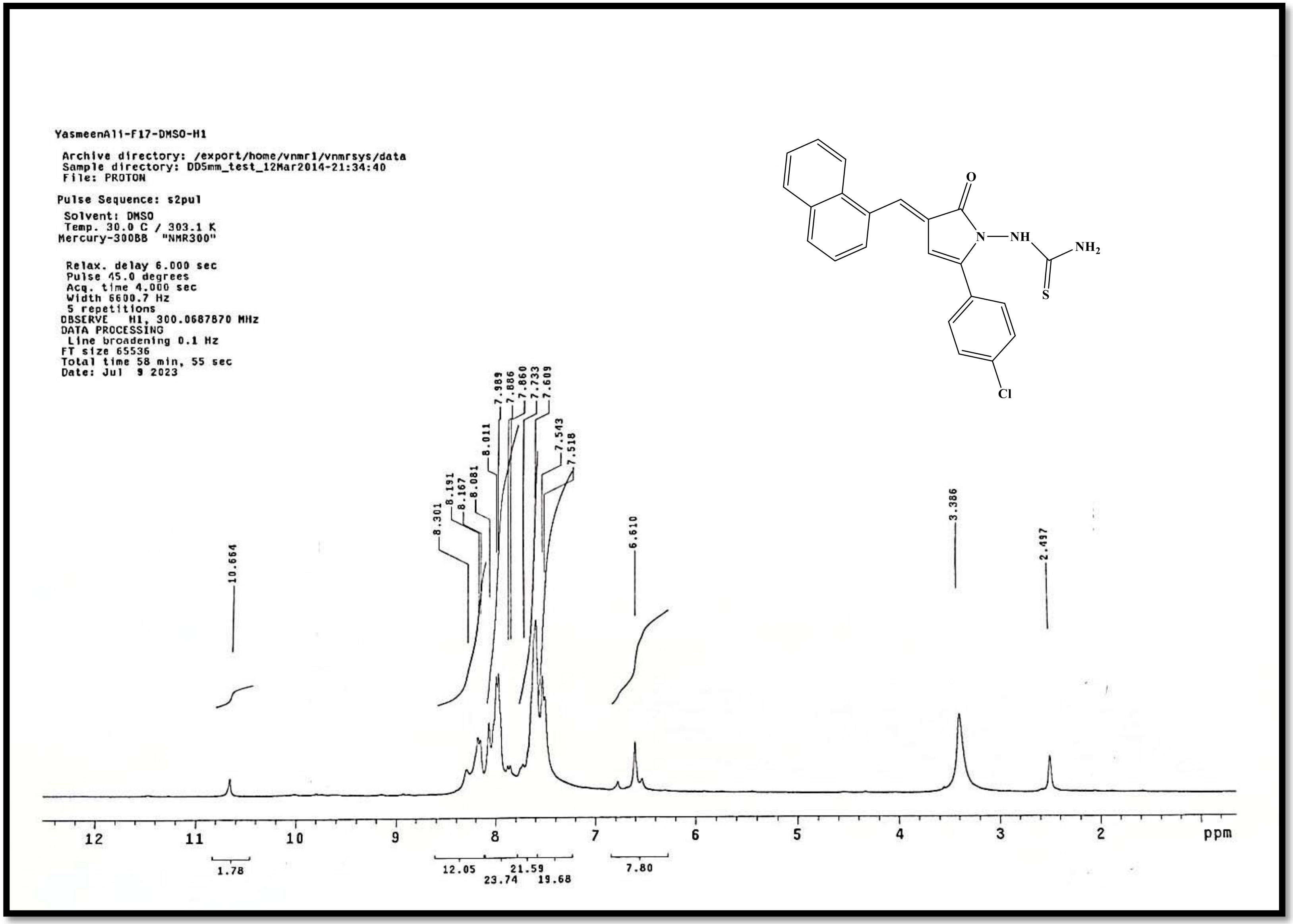

Figure 26S. ^{1}H-NMR (DMSO- d_6) of Compound (9)

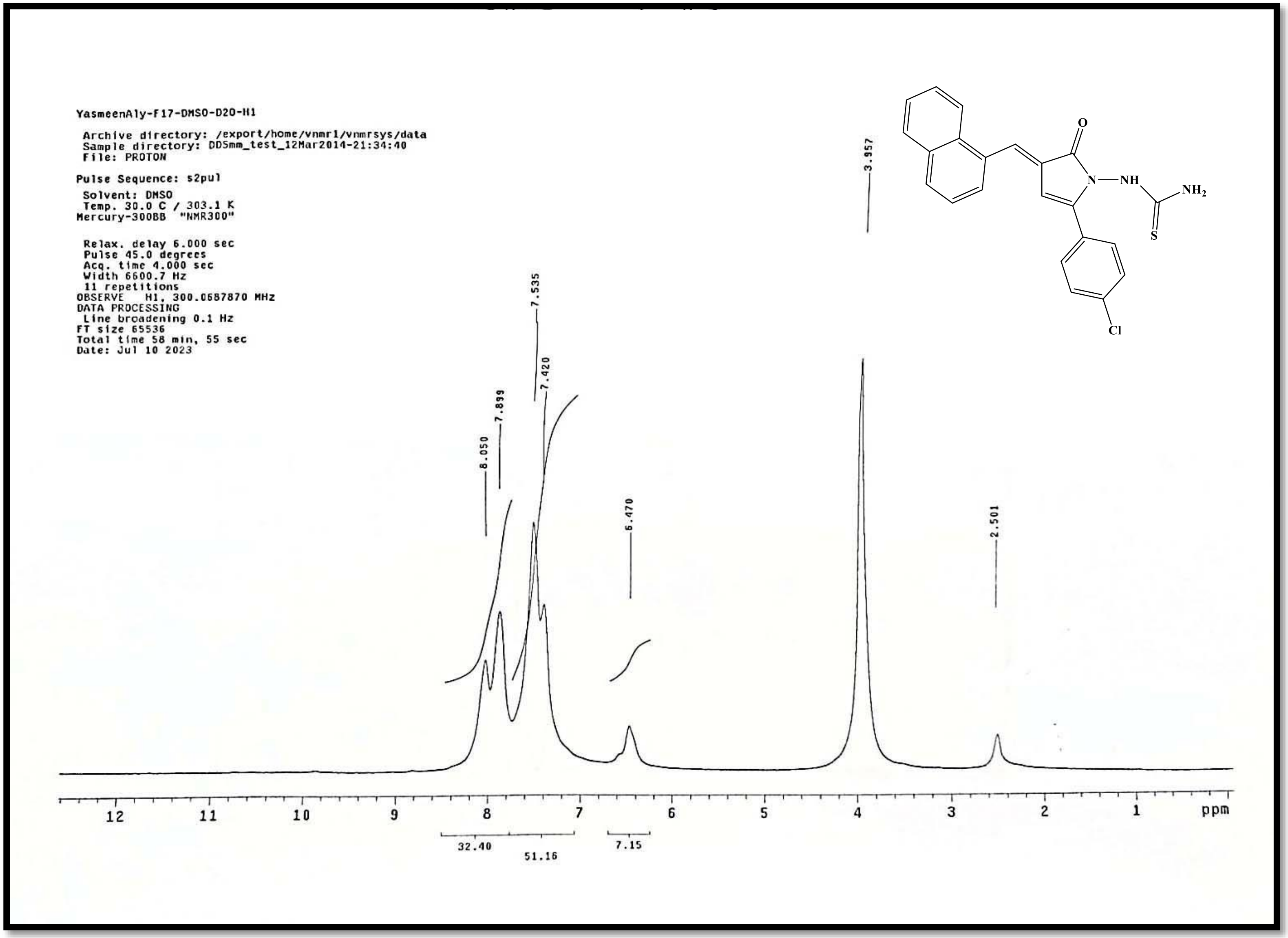

Figure 27S. ^{1}H-NMR spectrum (DMSO-d_6 + D_2O) of Compound (9)

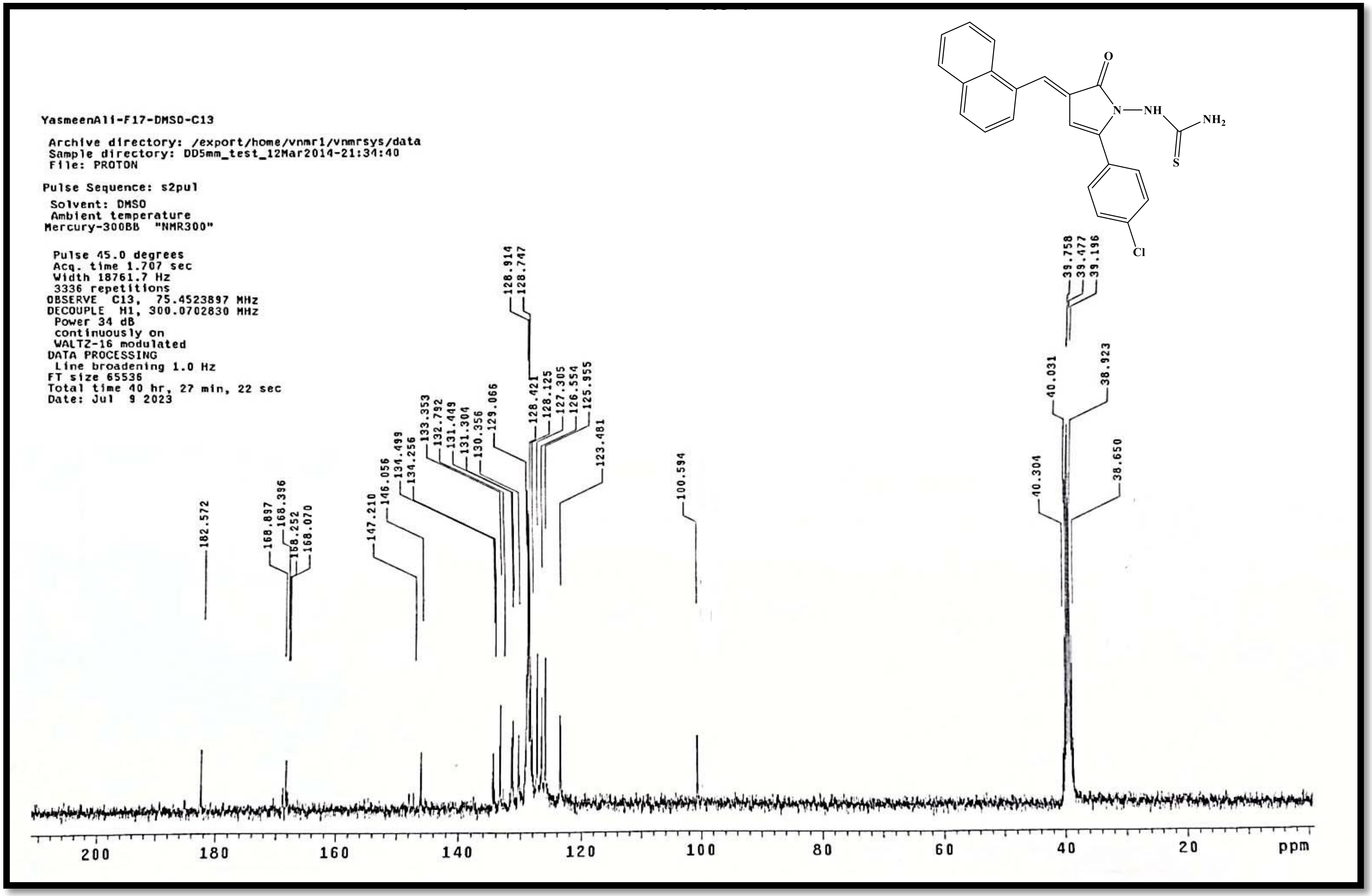

Figure 28S. ^{13}C-NMR spectrum (DMSO-d_6) of Compound (9)

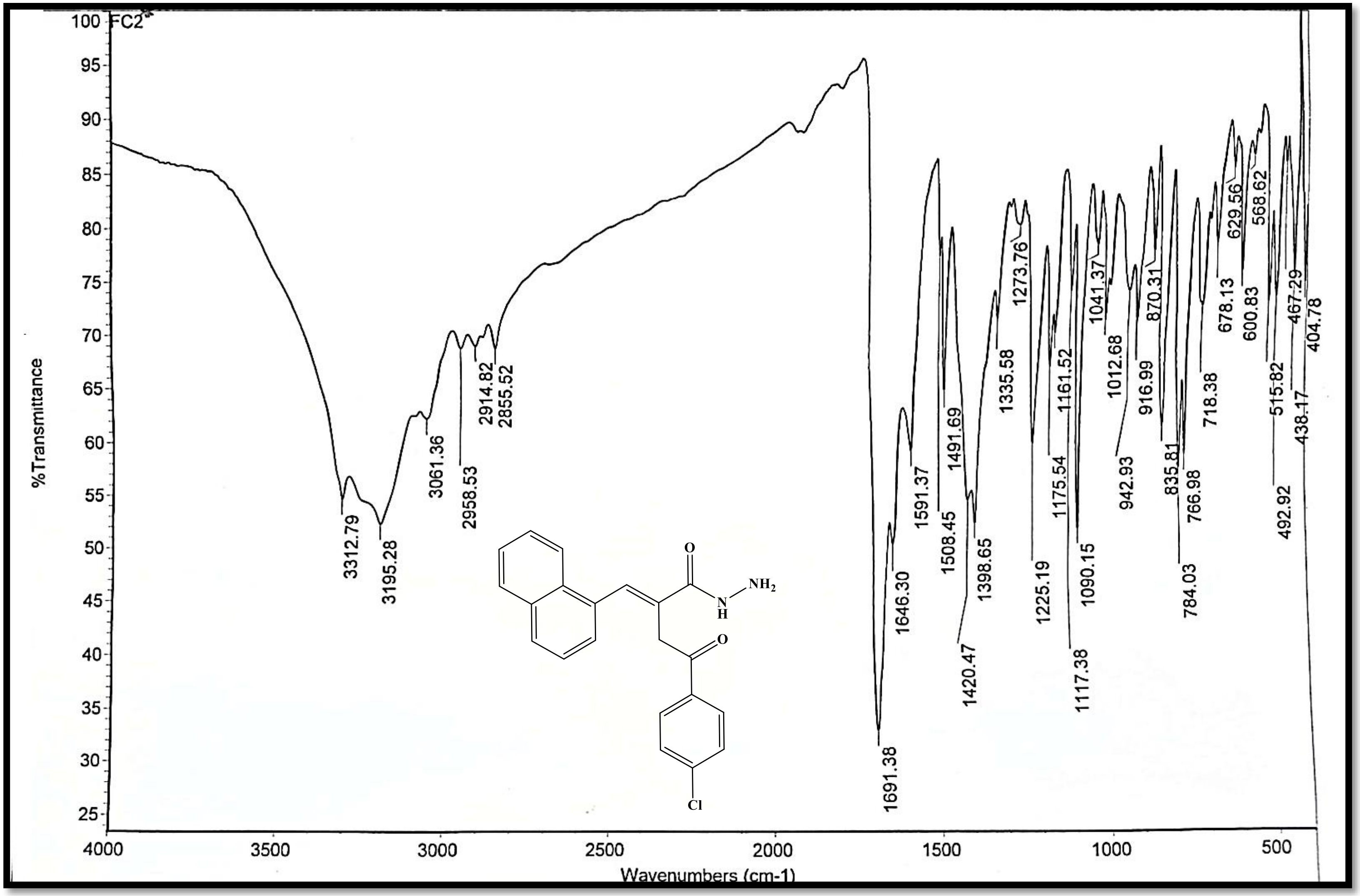

Figure 29S. IR spectrum of compound (10)

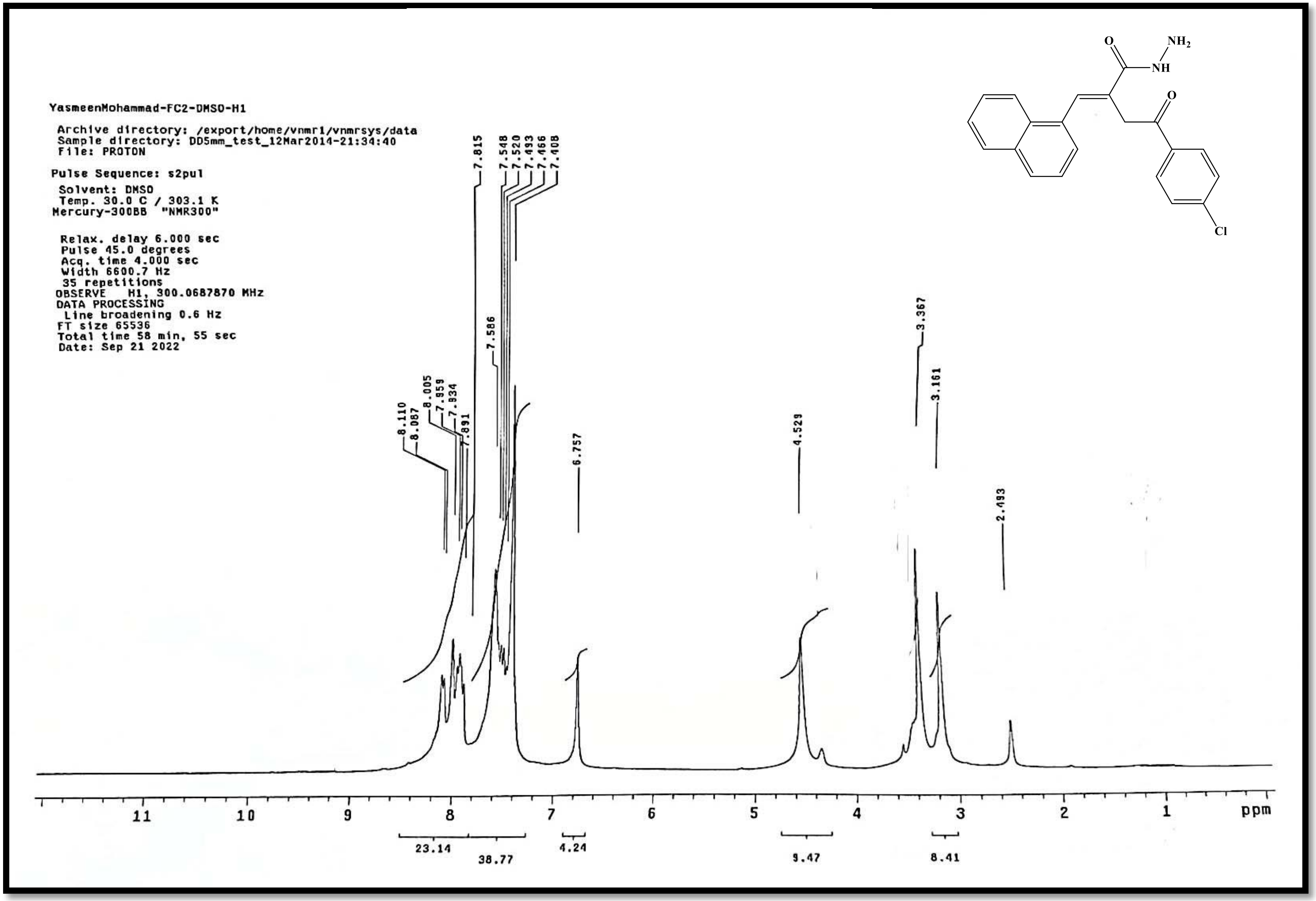

Figure 30S. ^{1}H-NMR (DMSO- d_6) of Compound (10)

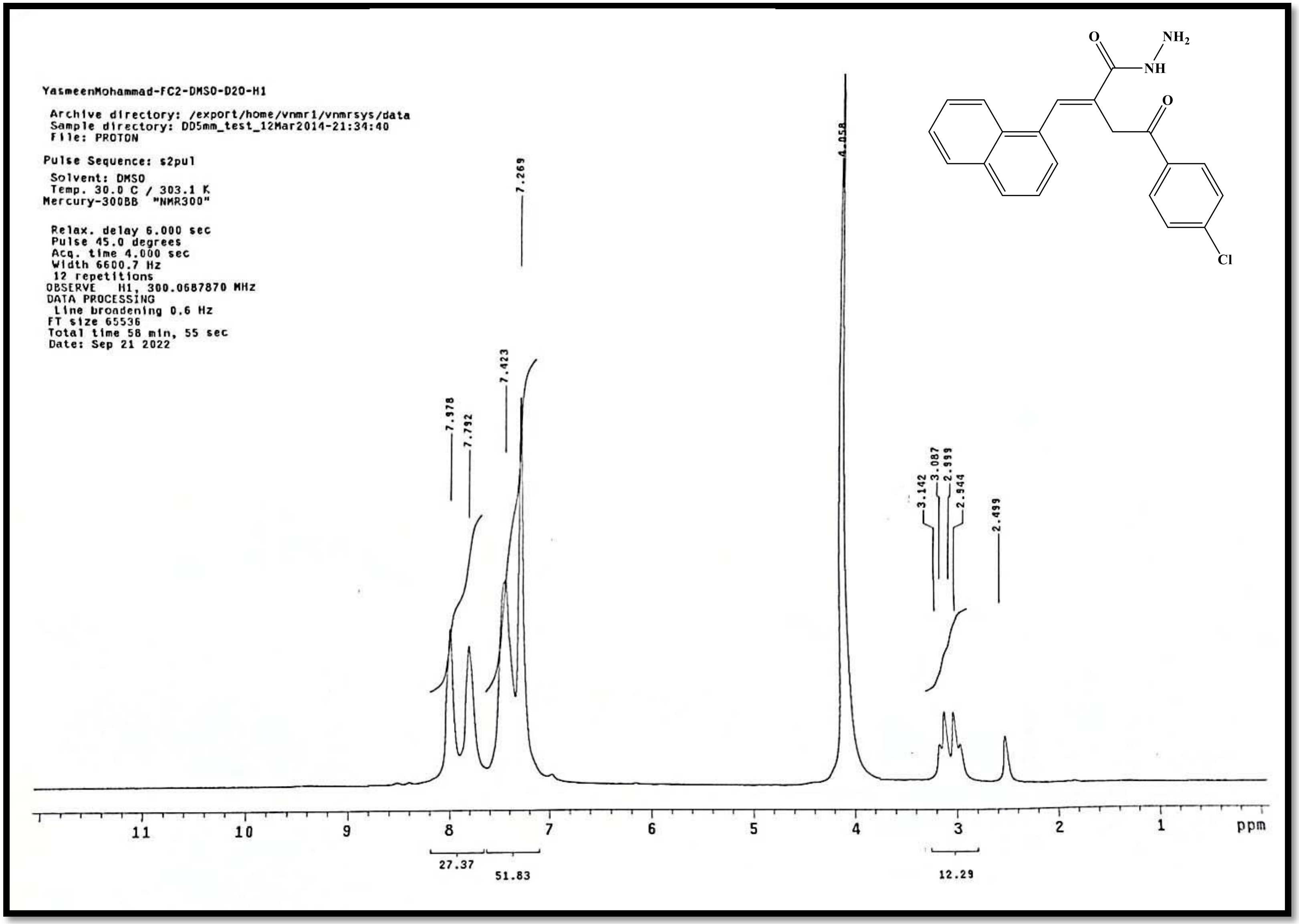

Figure 31S. ^{1}H-NMR spectrum (DMSO-d_6 + D_2O) of Compound (10)

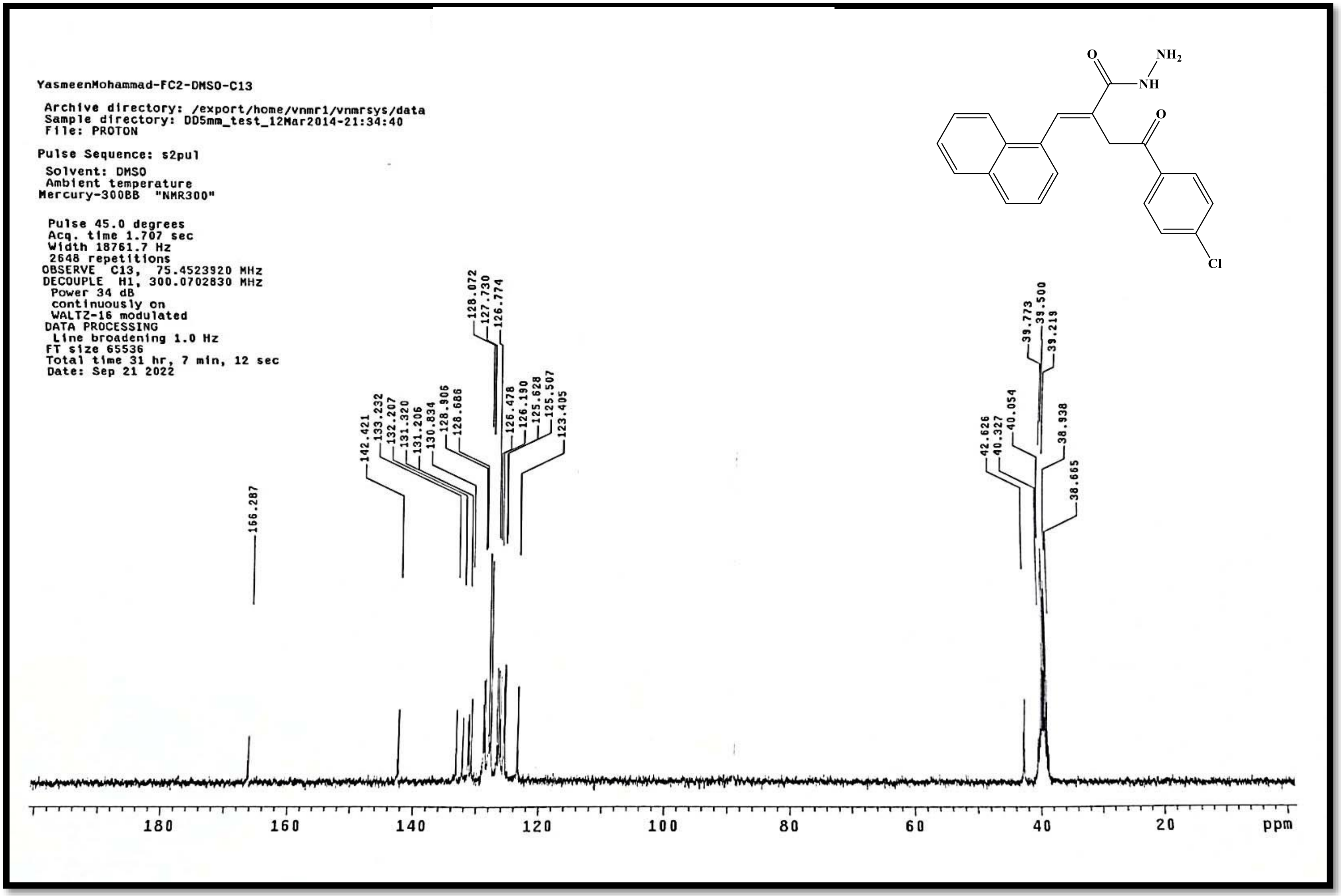

Figure 32S. ^{13}C-NMR spectrum (DMSO-d$_6$) of Compound (10)

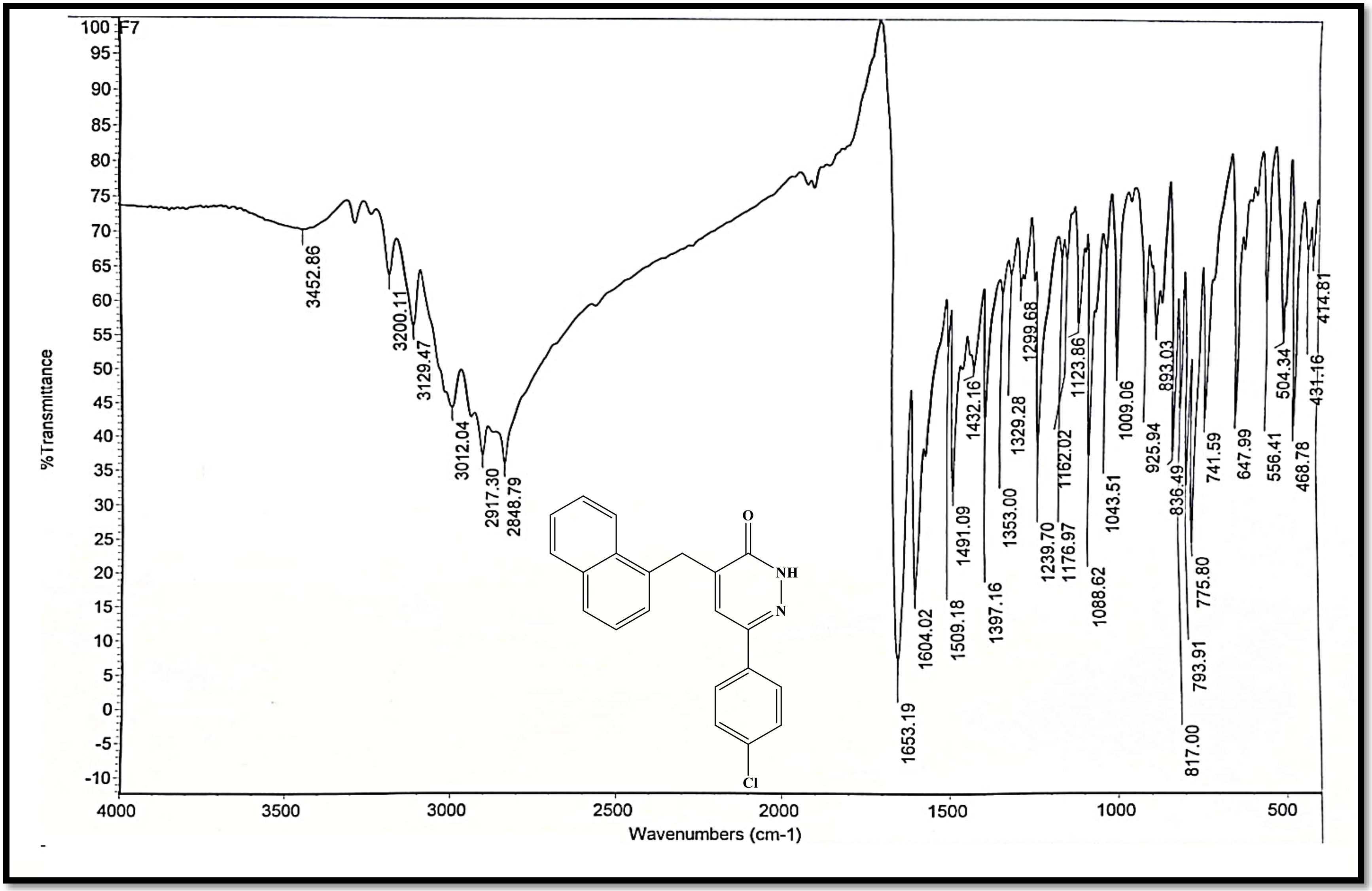

Figure 33S. IR spectrum of compound (11)

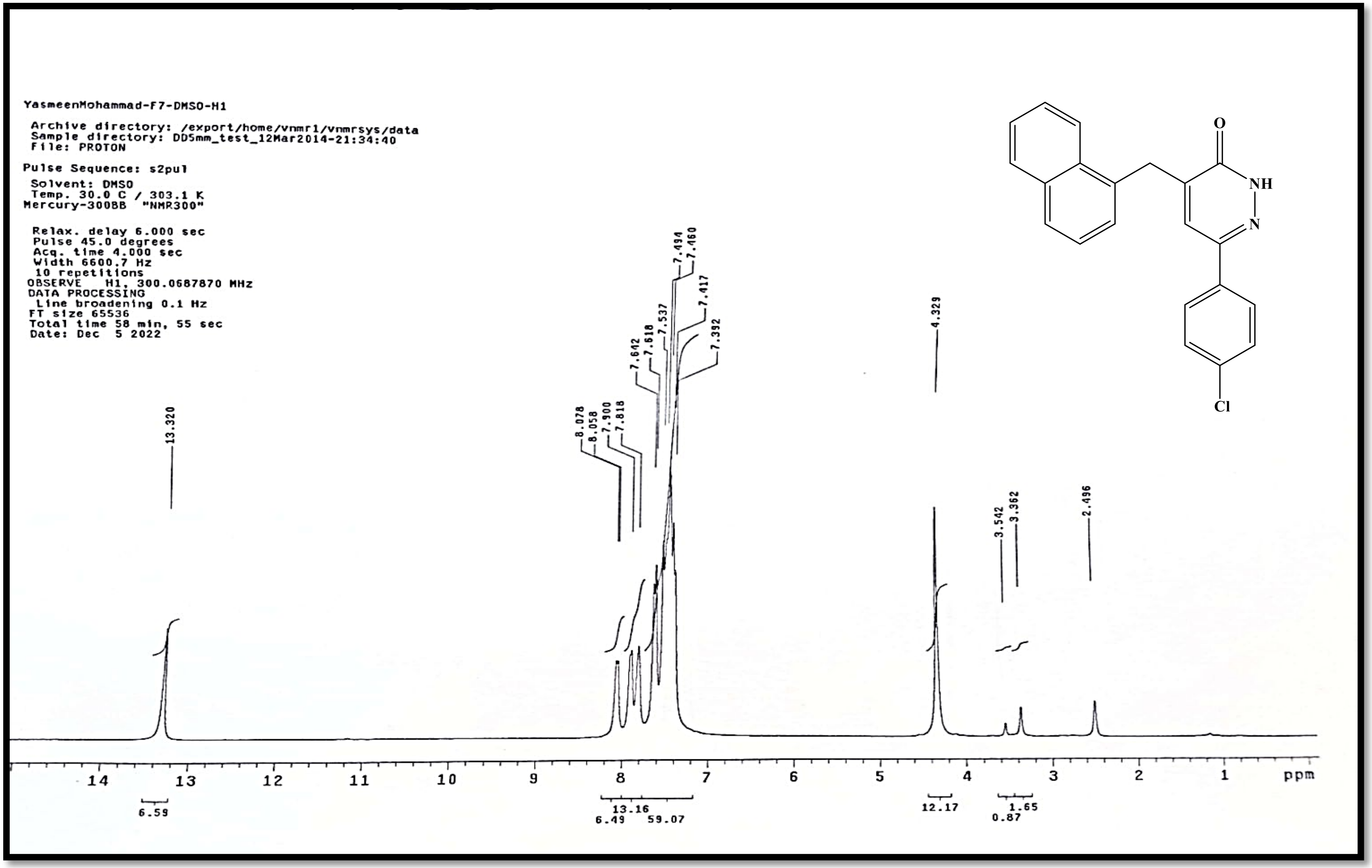

Figure 34S. ^{1}H-NMR (DMSO- d_6) of Compound (11)

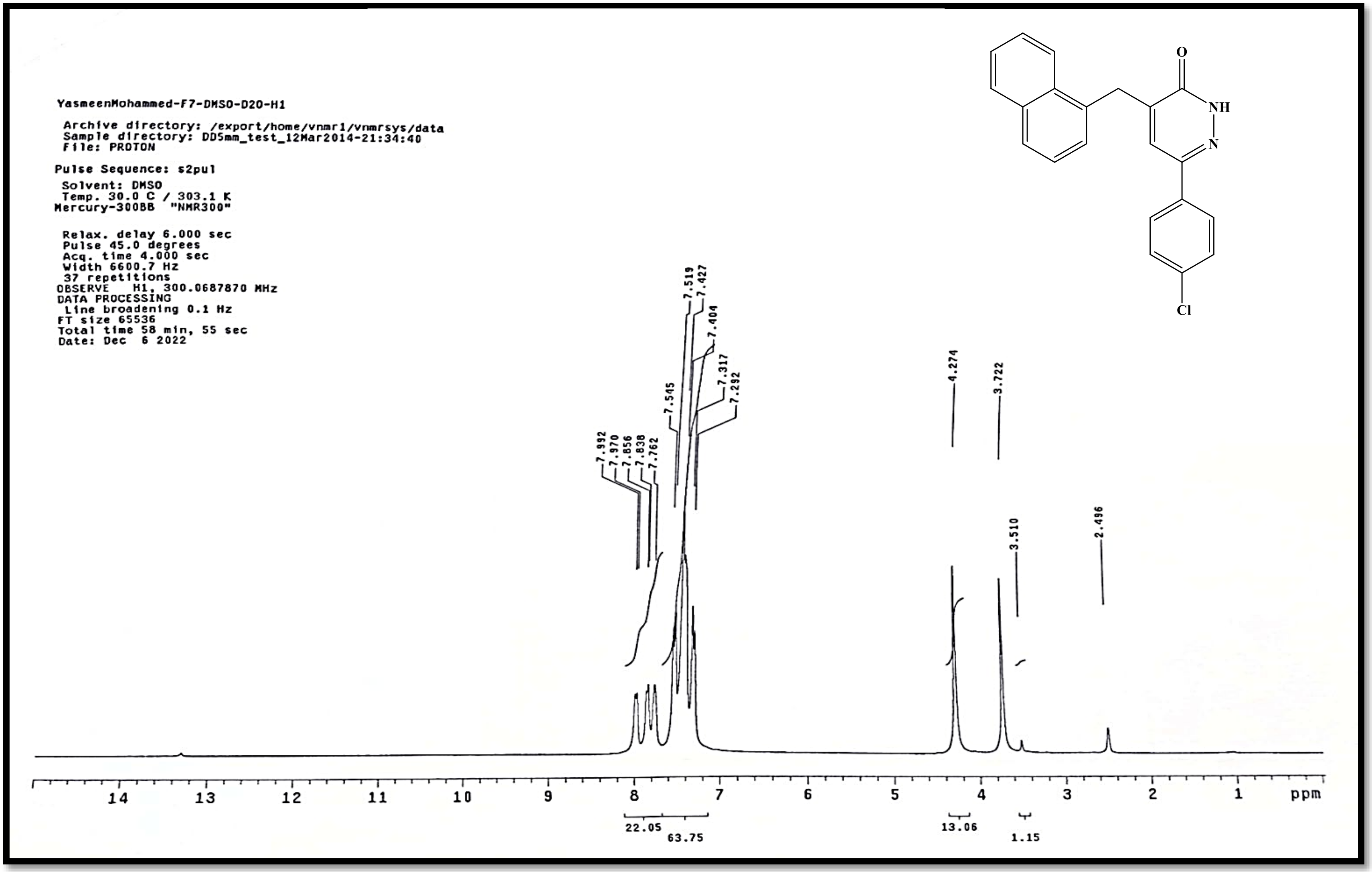

Figure 35S. ^{1}**H-NMR spectrum (DMSO-d$_6$ + D$_2$O) of Compound (11)**

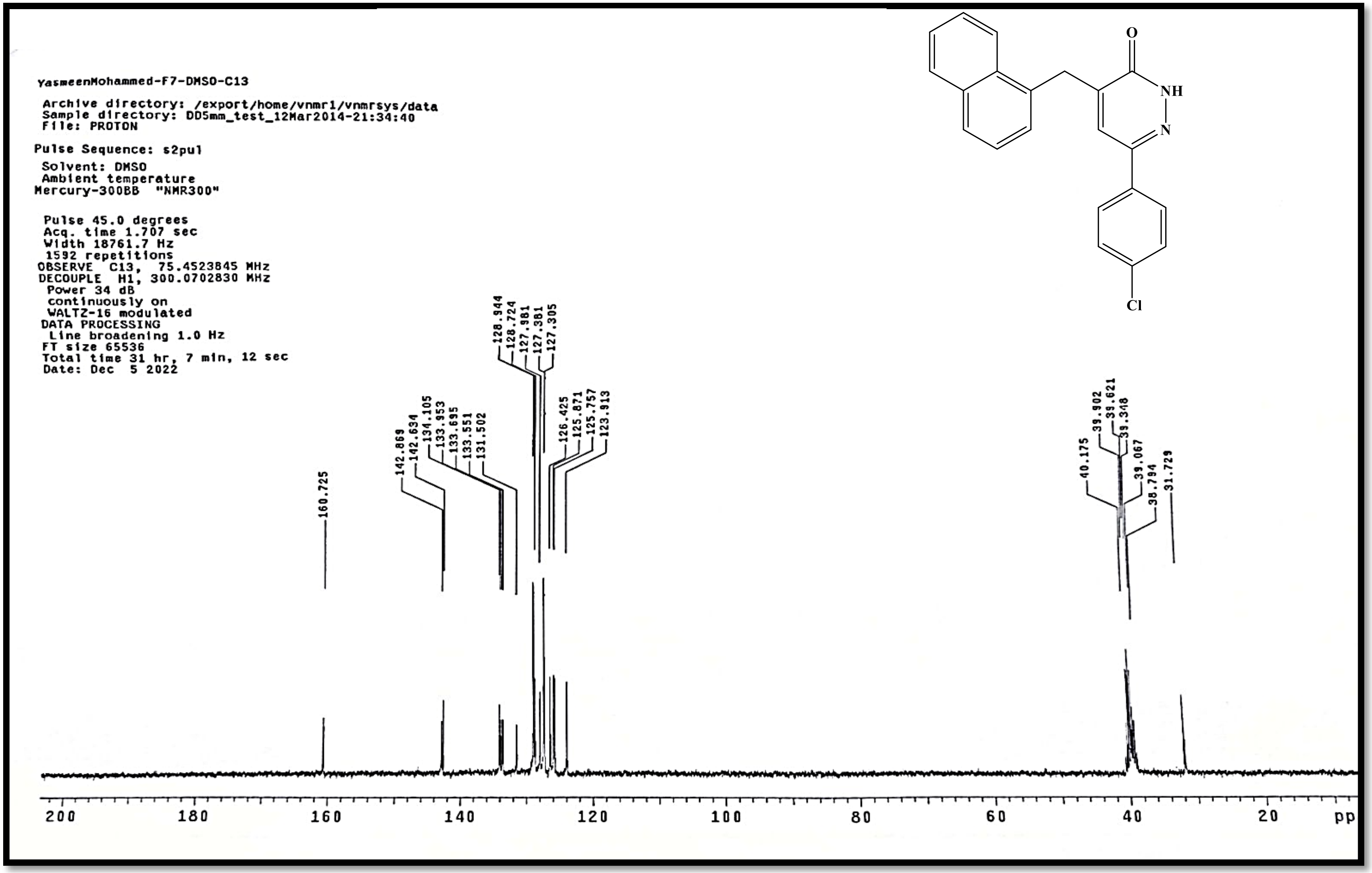

Figure 36S. ^{13}C-NMR spectrum (DMSO-d_6) of Compound (11)

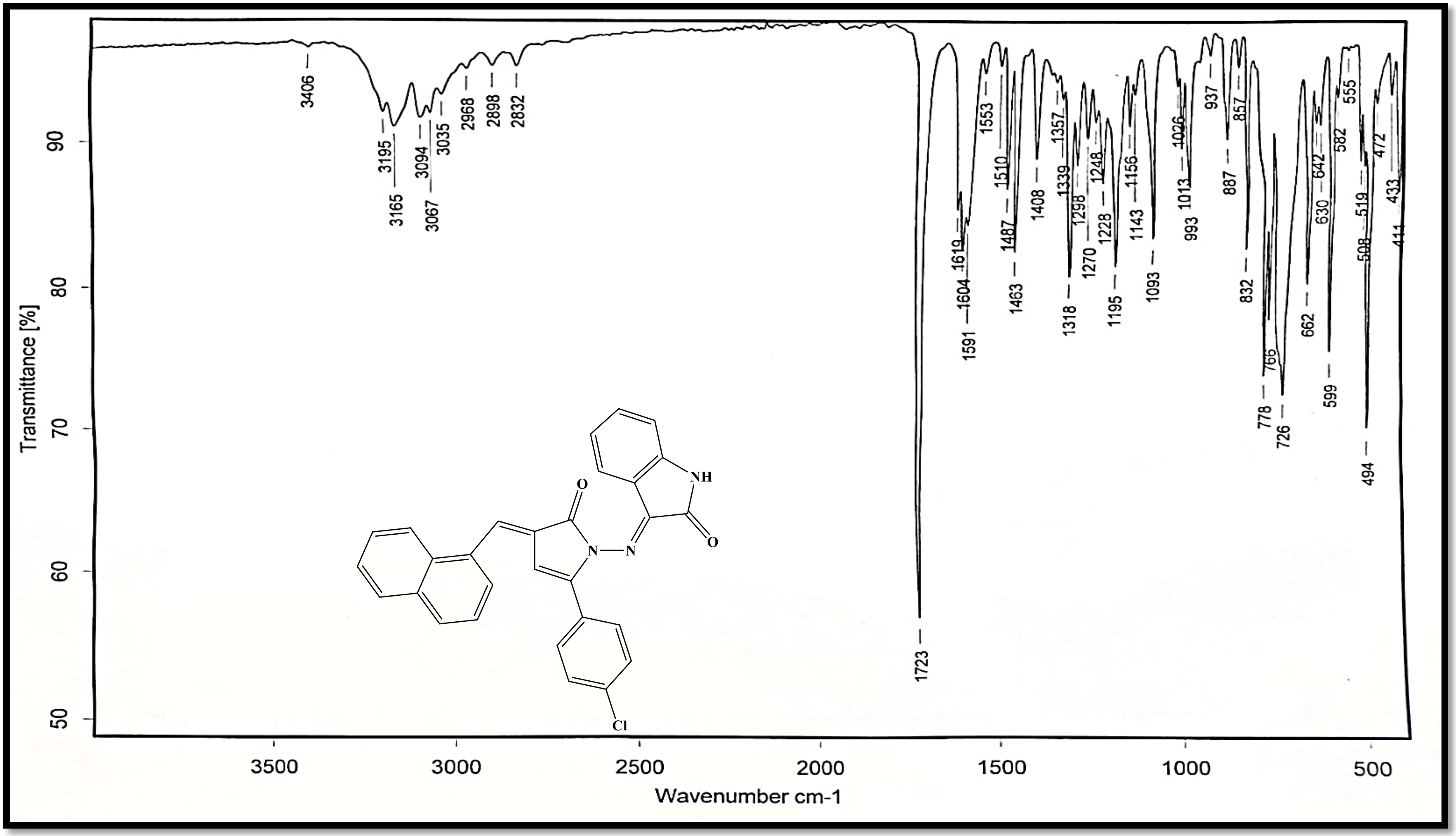

Figure 37S. IR spectrum of compound (12)

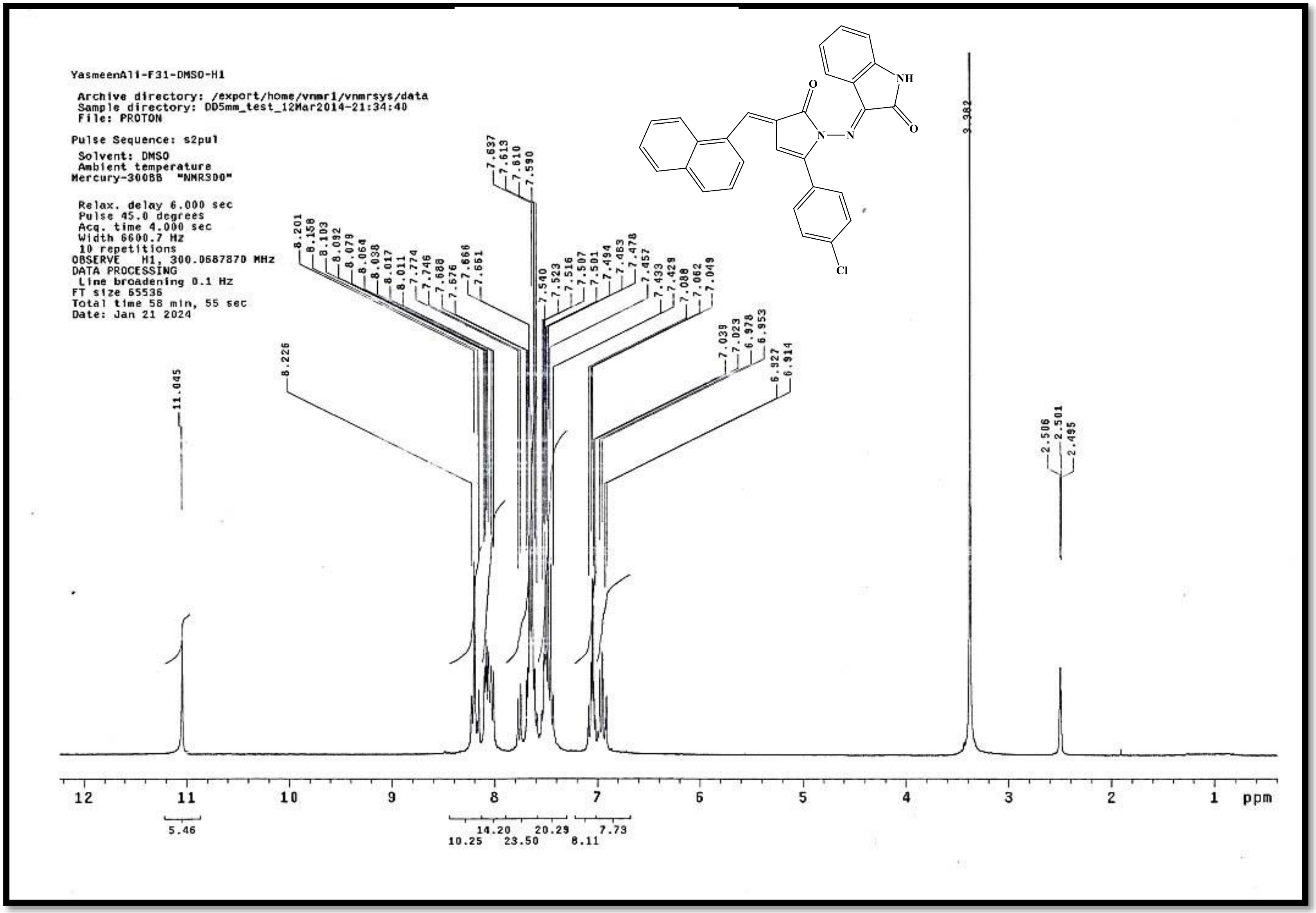

Figure 38S. ^{1}H-NMR (DMSO- d_6) of Compound (12)

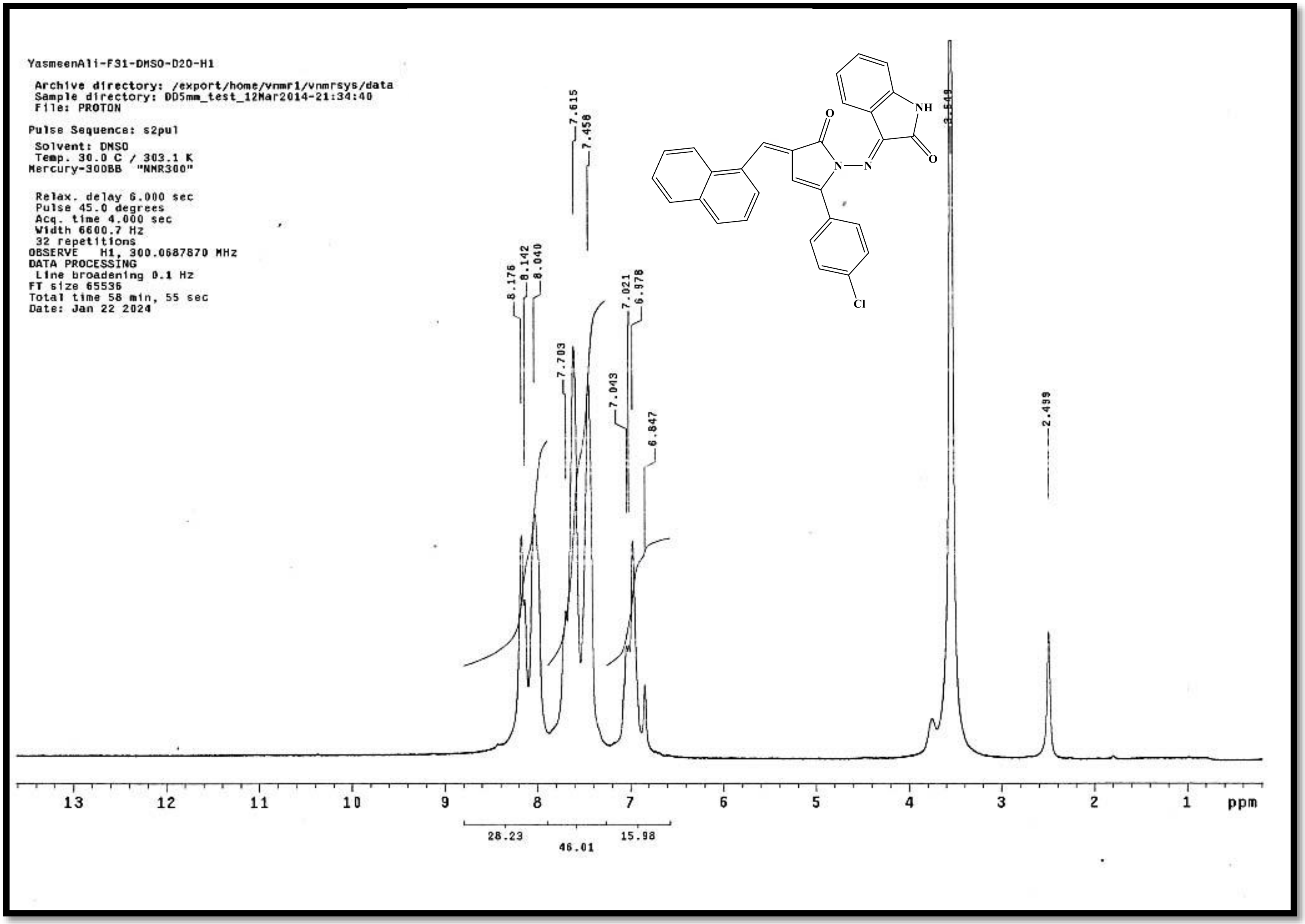

Figure 39S. ^{1}H-NMR spectrum (DMSO-d_6 + D_2O) of Compound (12)

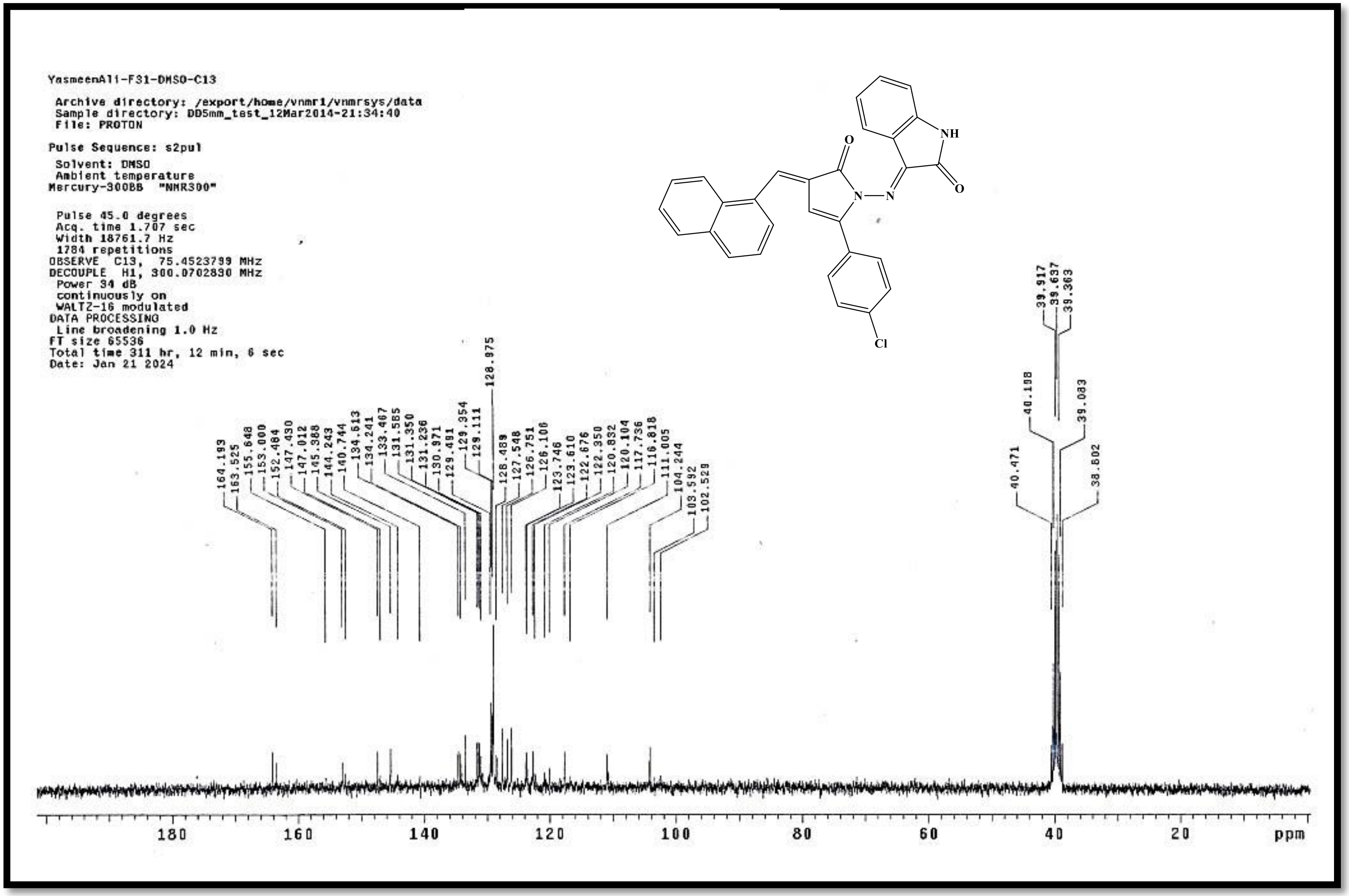

Figure 40S. ^{13}C-NMR spectrum (DMSO-d_6) of Compound (12)

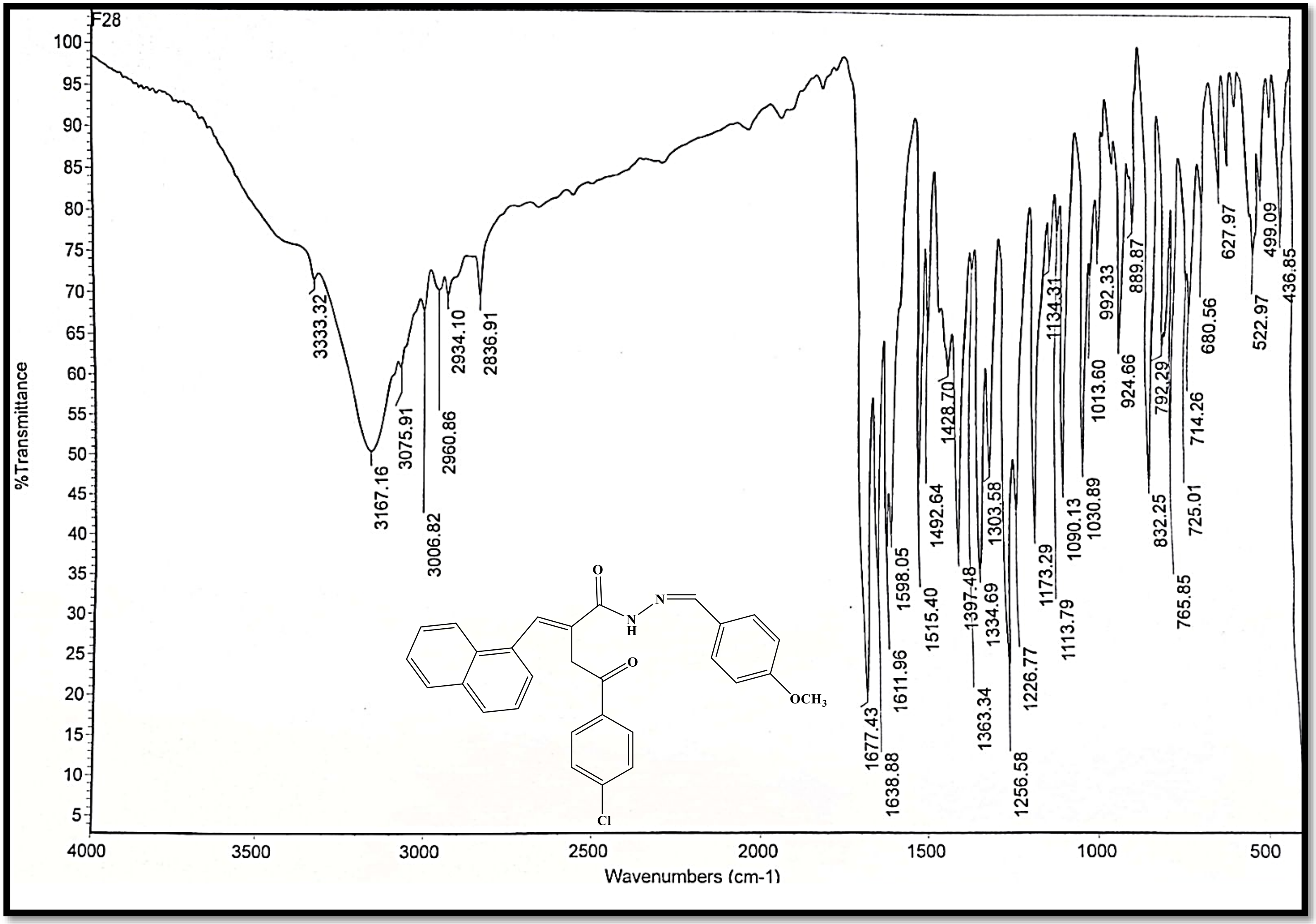

Figure 41S. IR spectrum of compound (13)

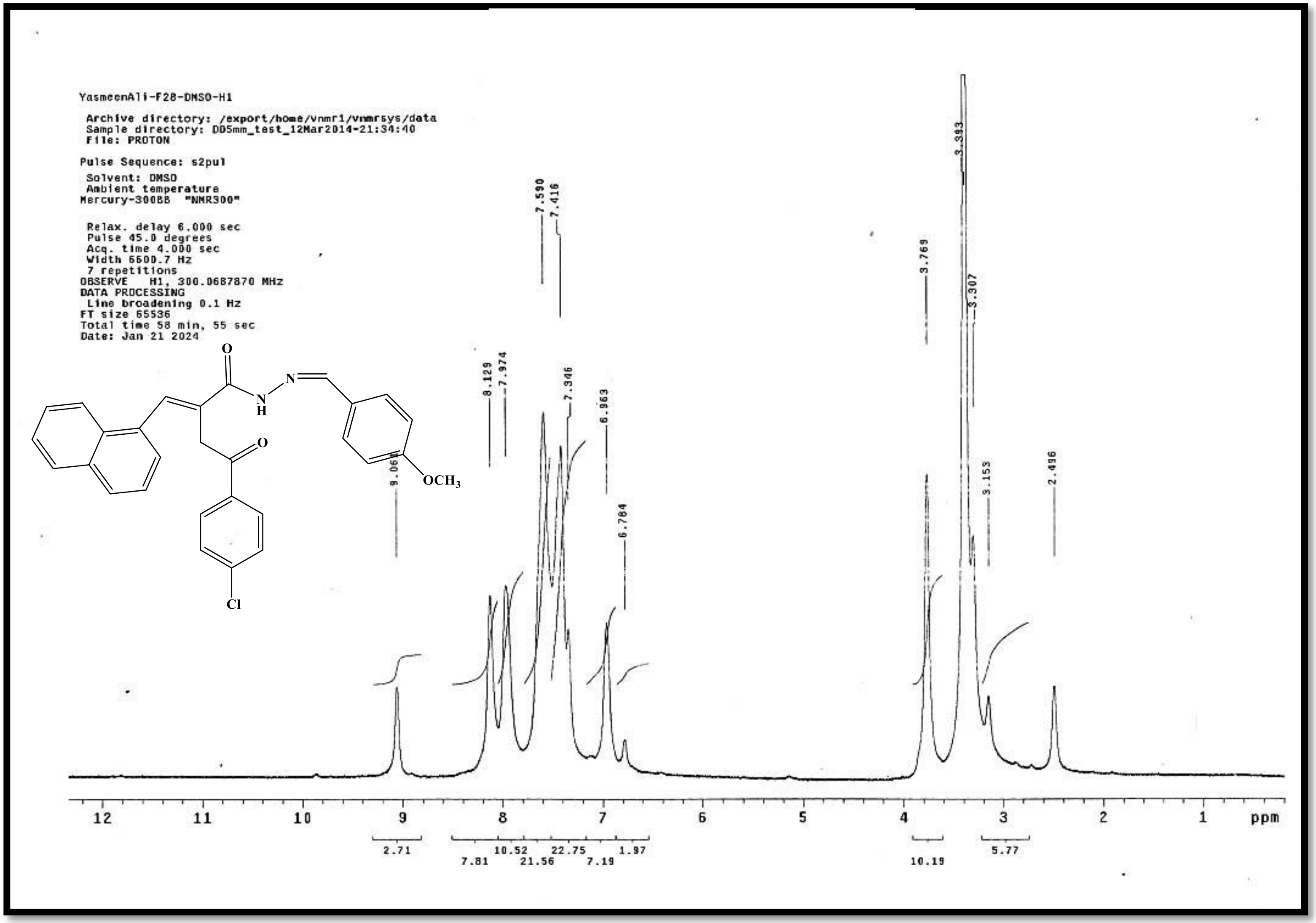

Figure 42S. ^{1}H-NMR (DMSO- d_6) of Compound (13)

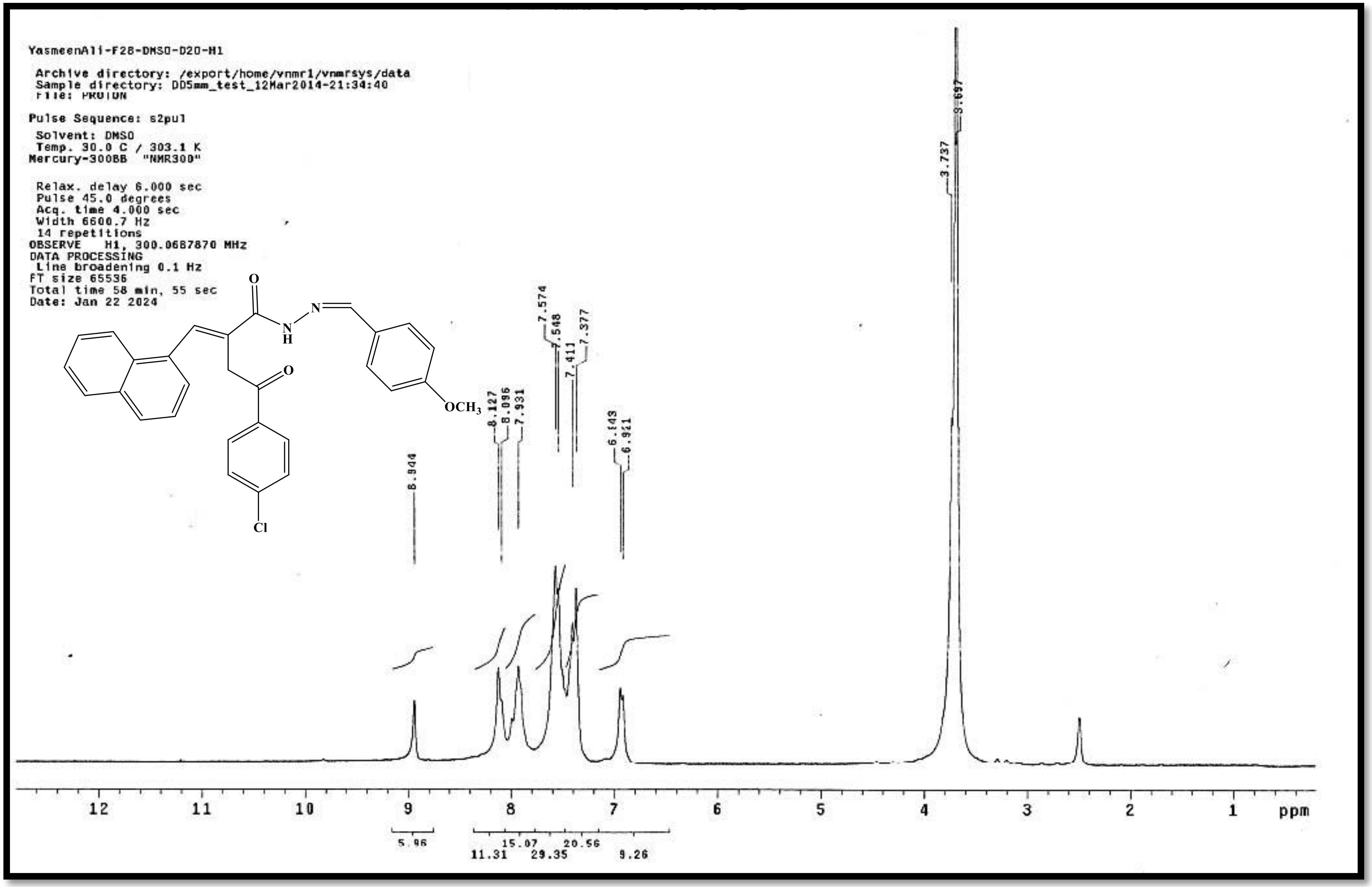

Figure 43S. **^{1}H-NMR spectrum (DMSO-d_6 + D_2O) of Compound (13)**

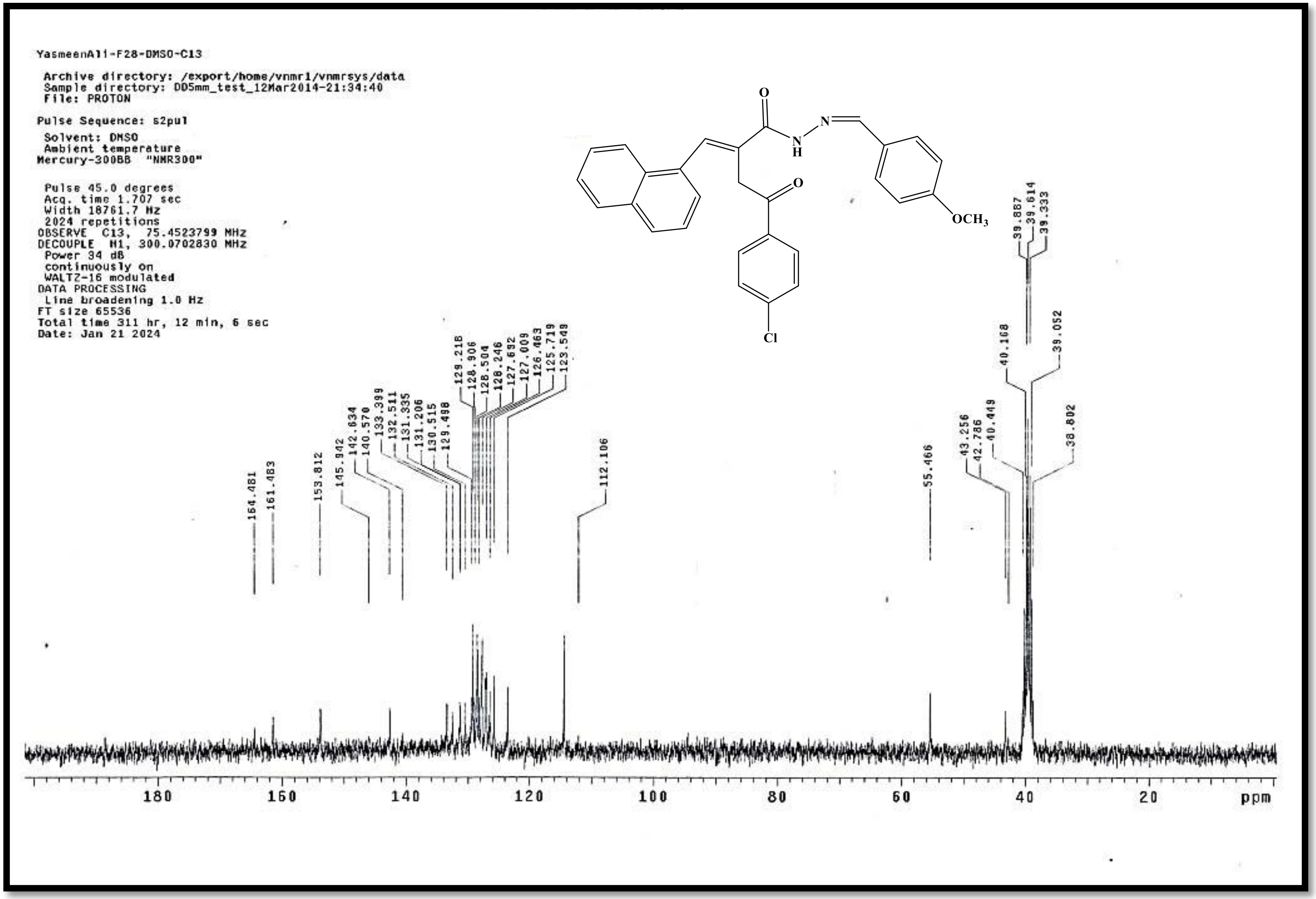

Figure 44S. ^{13}**C-NMR spectrum (DMSO-d**$_6$**) of Compound (13)**

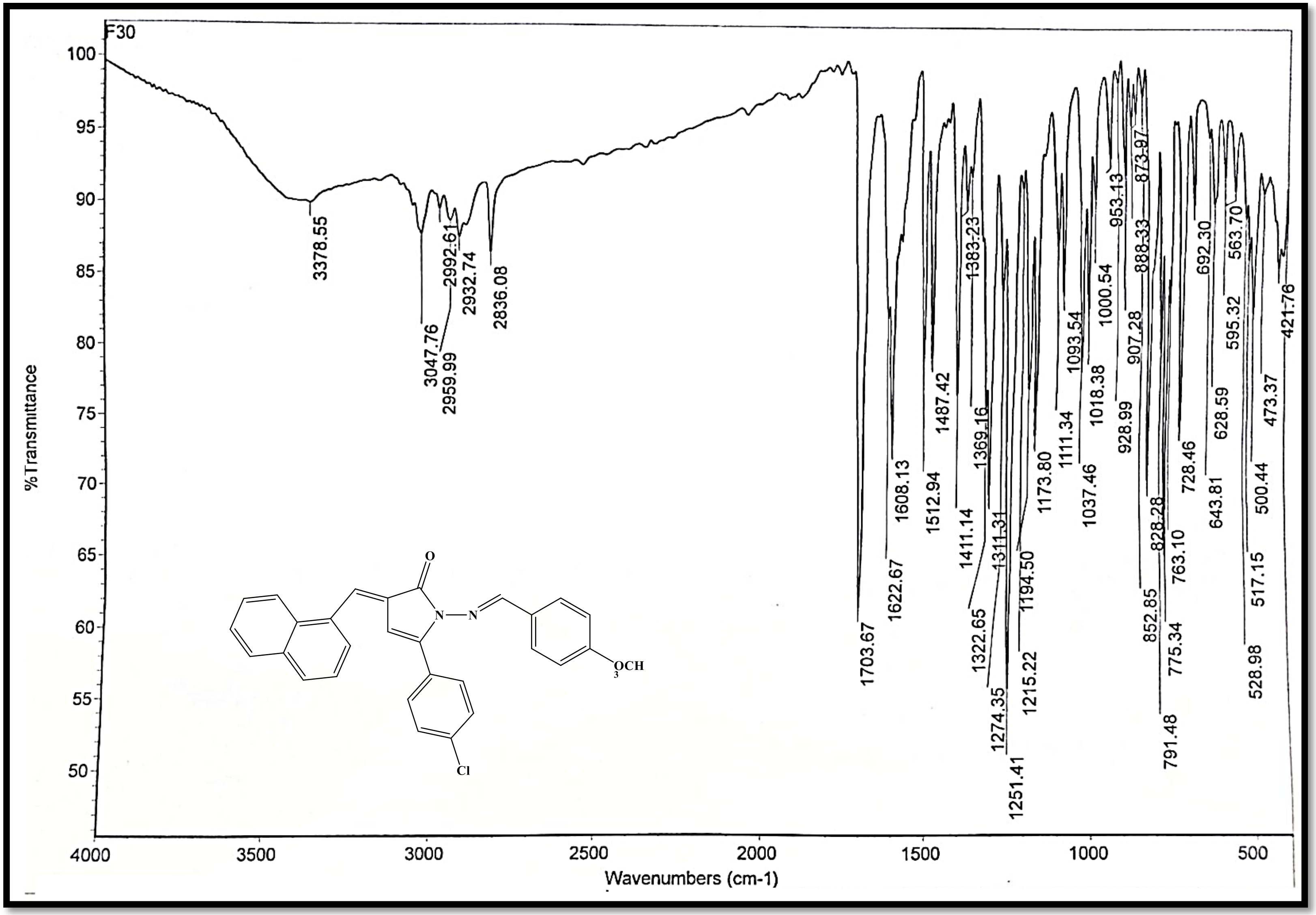

Figure 45S. IR spectrum of compound (14)

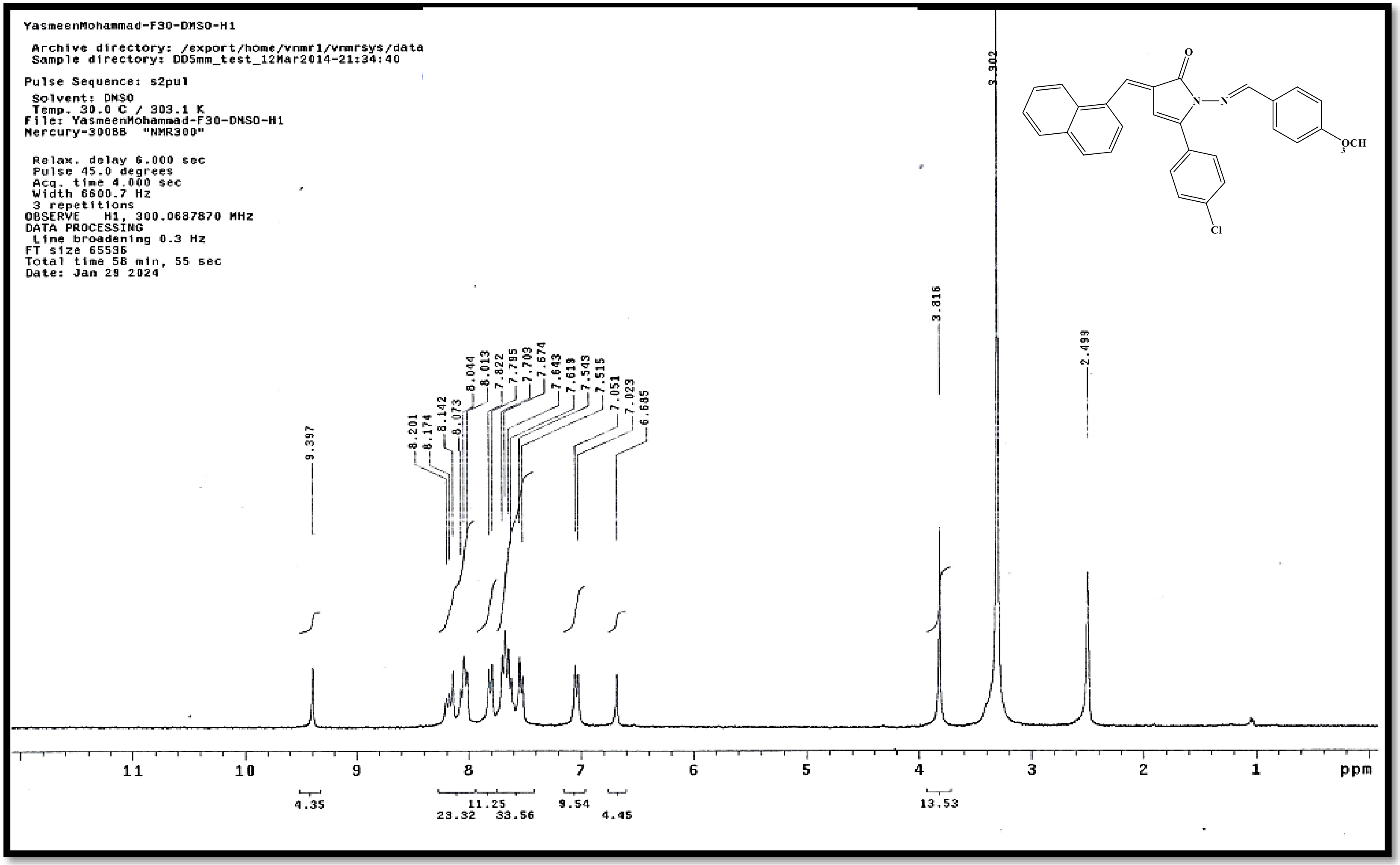

Figure 46S. ^{1}H-NMR (DMSO- d_6) of Compound (14)

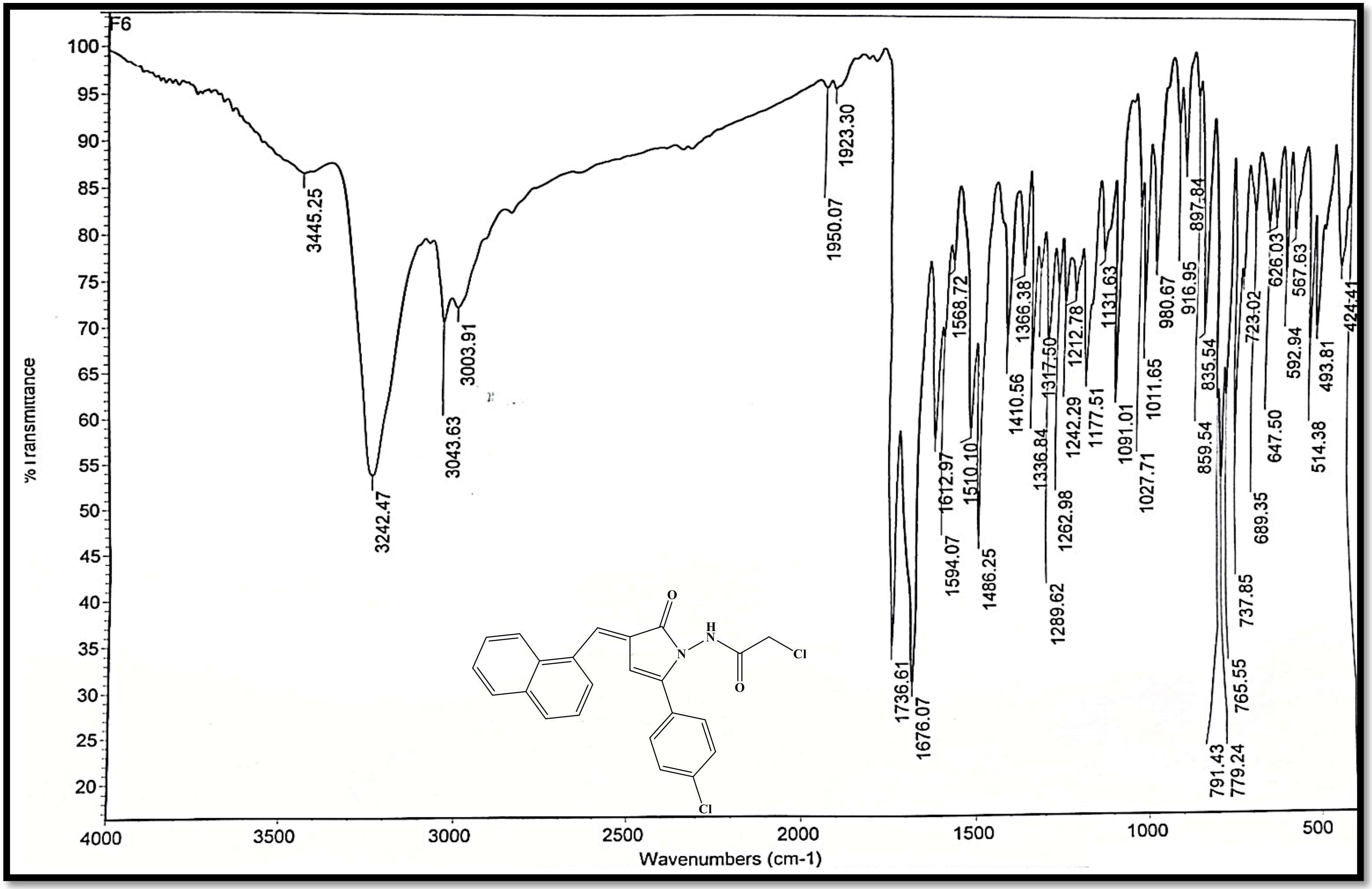

Figure 47S. IR spectrum of compound (15)

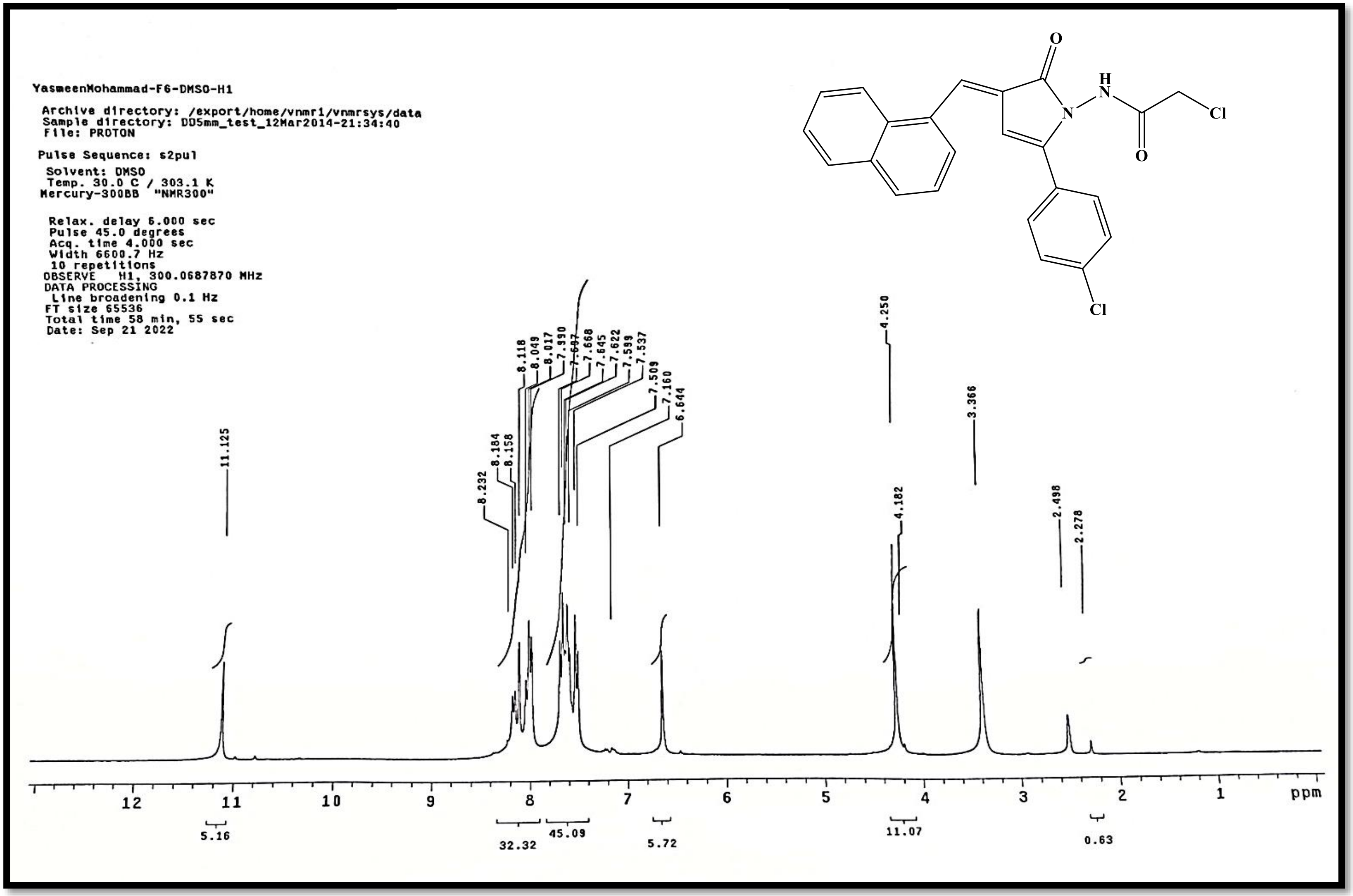

Figure 48S. ^{1}**H-NMR (DMSO- d_6) of Compound (15)**

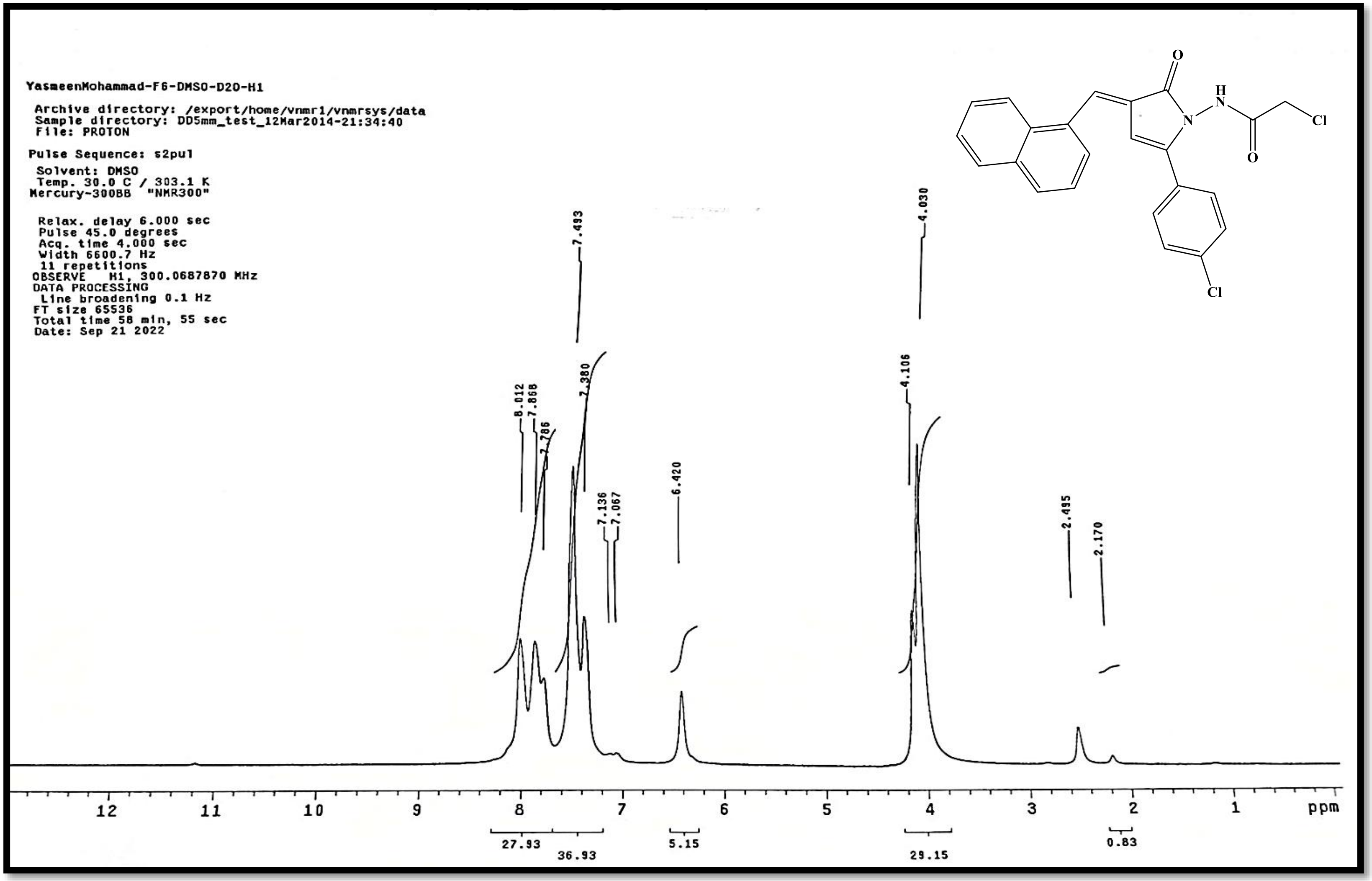

Figure 49S. ^{1}H-NMR spectrum (DMSO-d$_6$ + D$_2$O) of Compound (15)

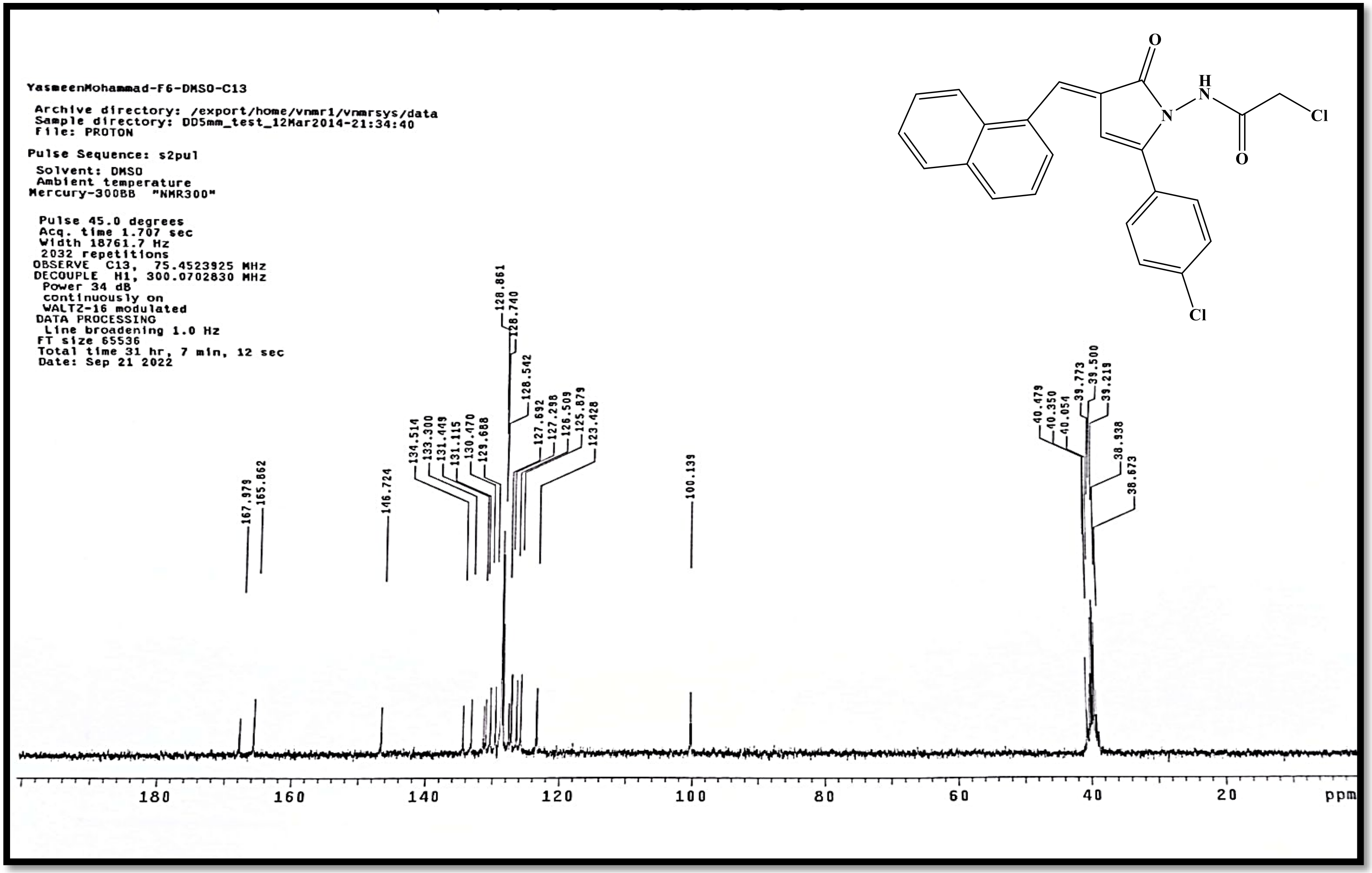

Figure 50S. ^{13}**C-NMR spectrum (DMSO-d**$_6$**) of Compound (15)**

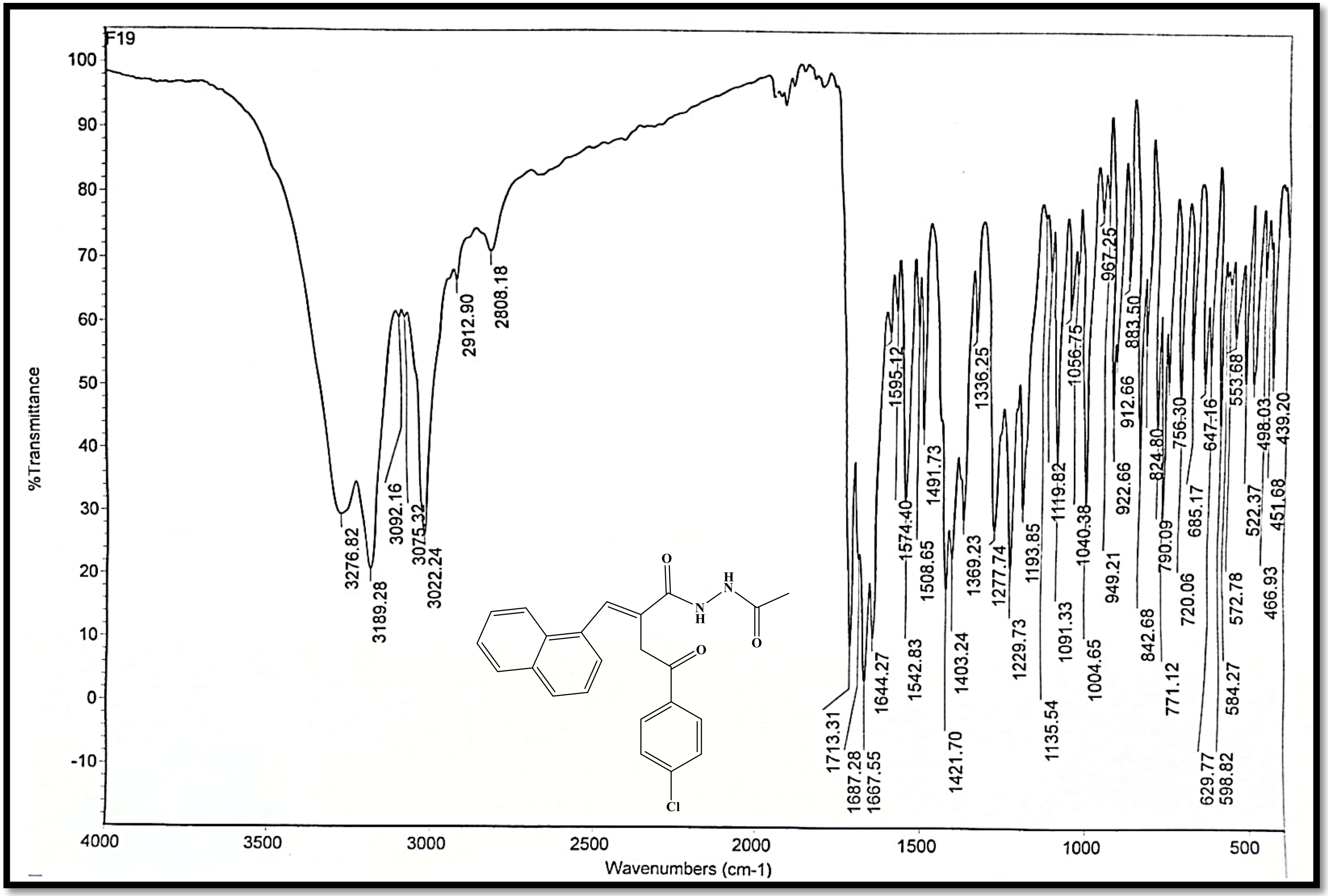

Figure 51S. IR spectrum of compound (16)

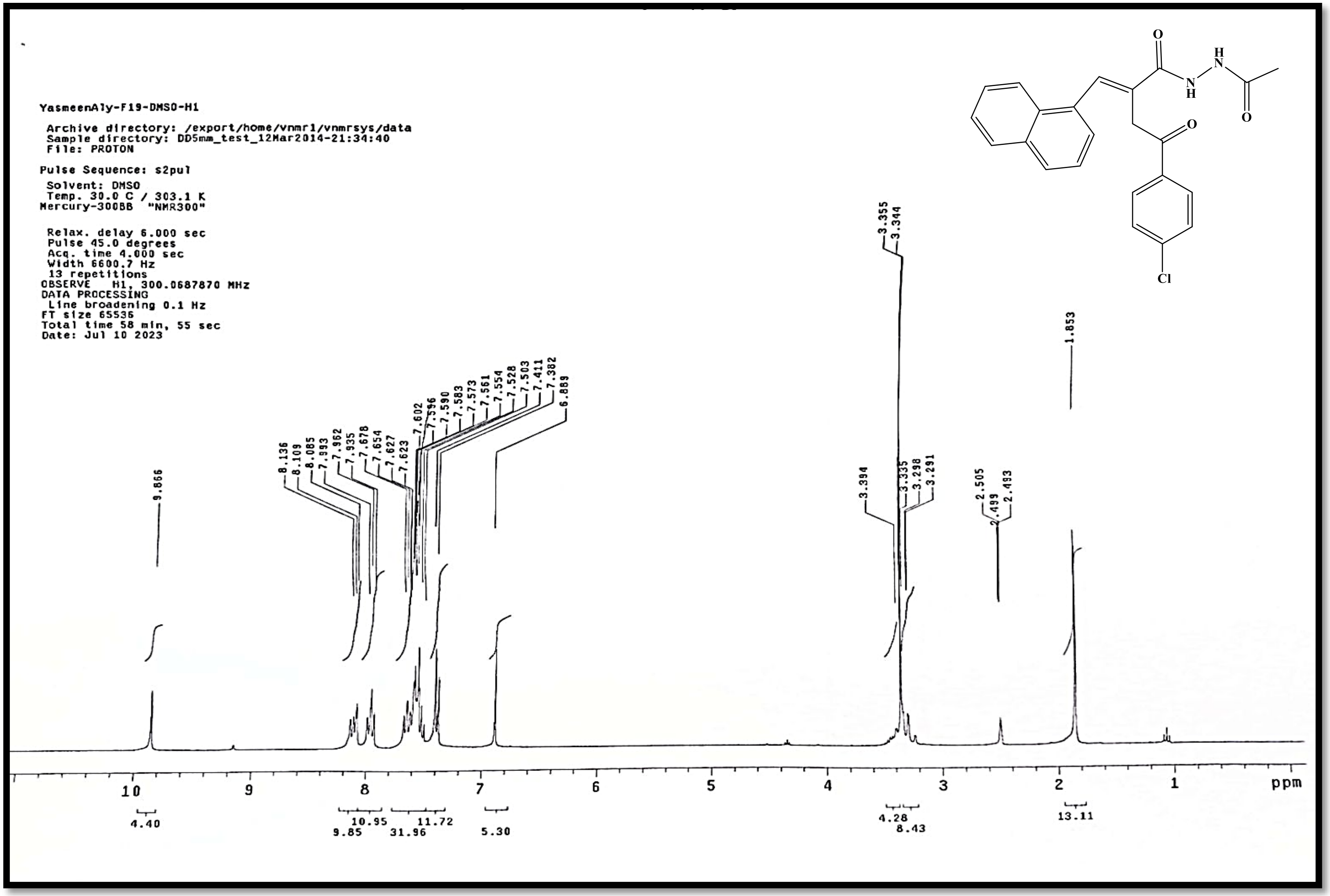

Figure 52S. ^{1}H-NMR (DMSO- d_6) of Compound (16)

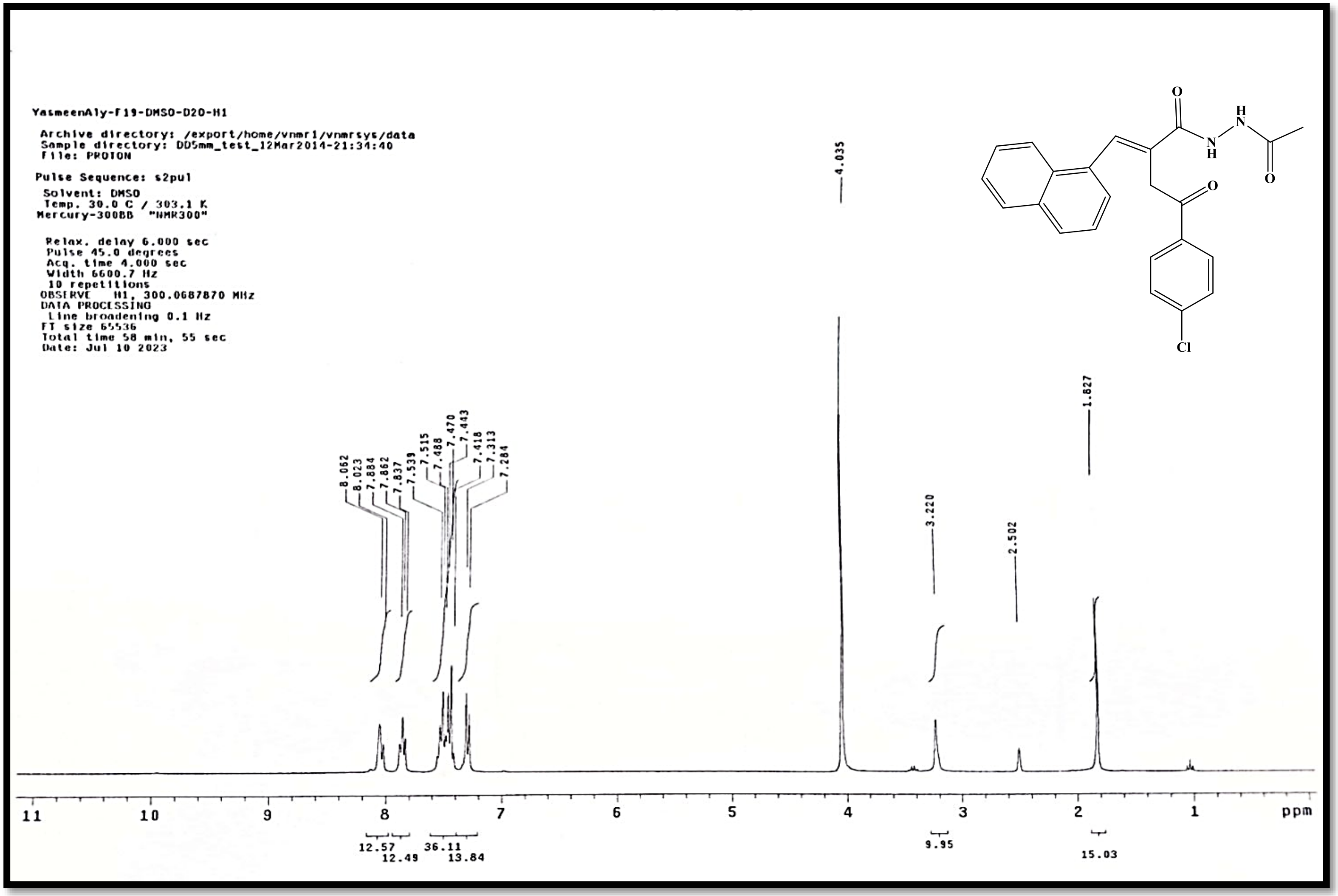

Figure 53S. ^{1}H-NMR spectrum (DMSO-d$_6$ + D$_2$O) of Compound (16)

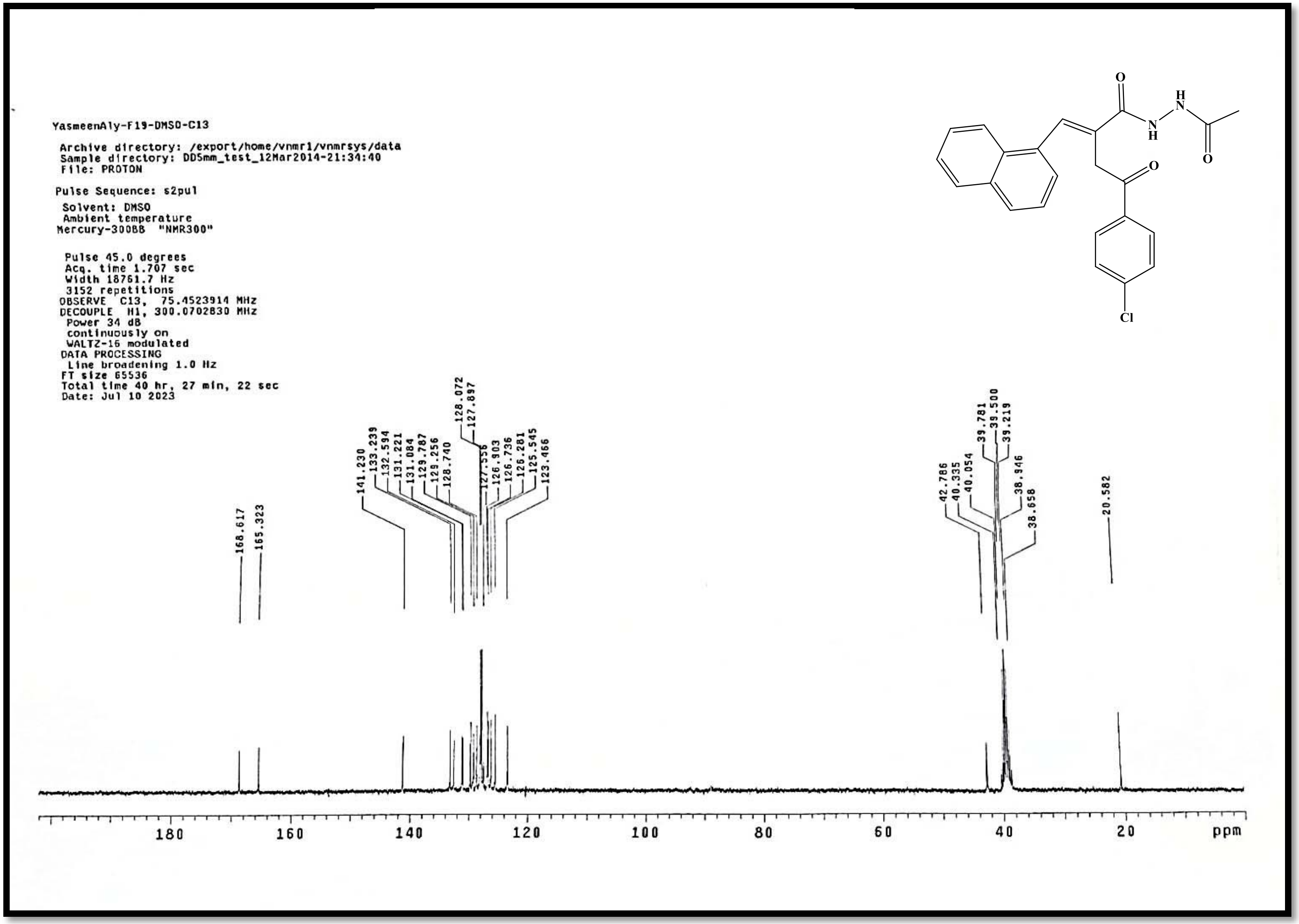

Figure 54S. ^{13}**C-NMR spectrum (DMSO-d**$_6$**) of Compound (16)**

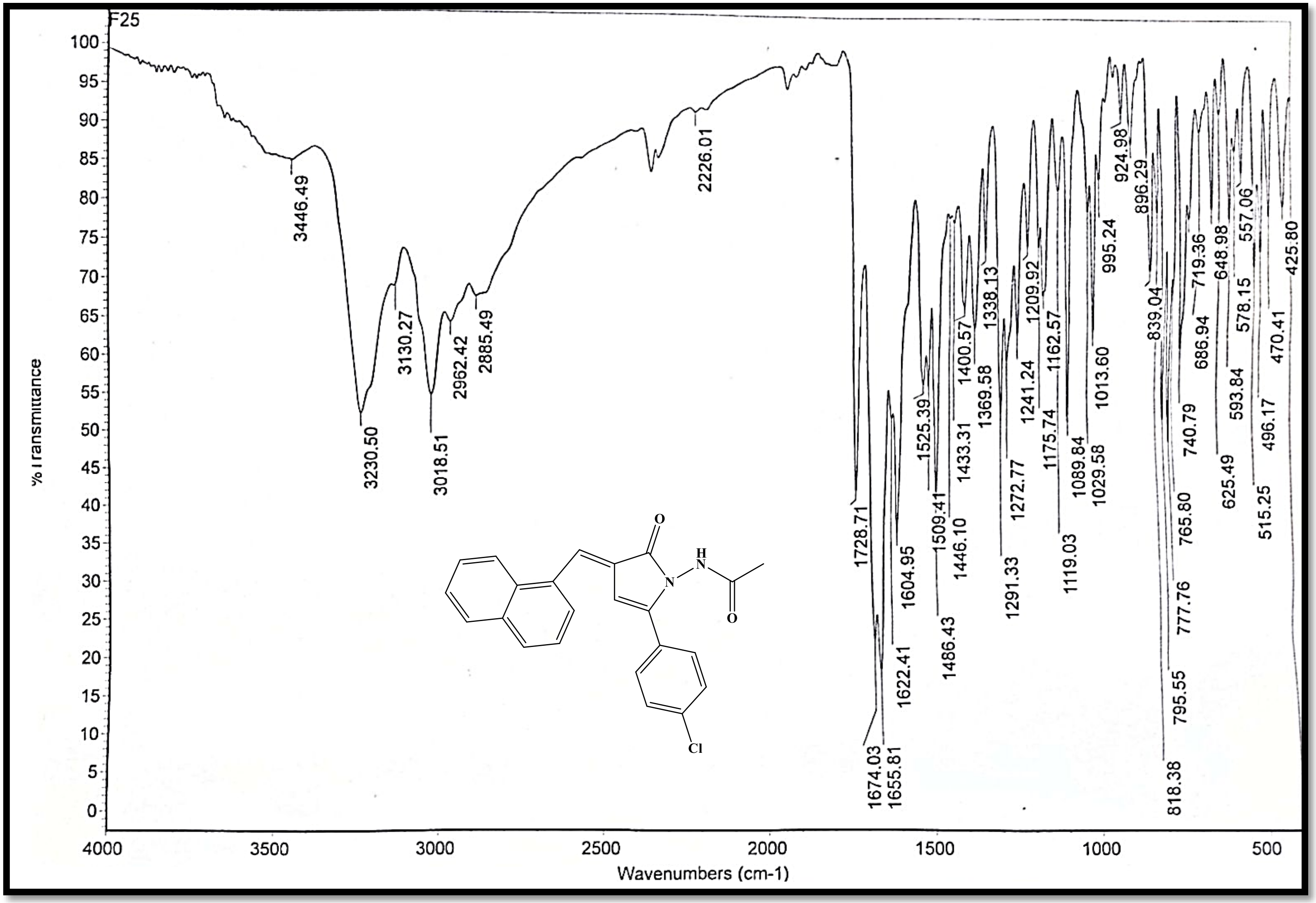

Figure55. IR spectrum of compound (17)

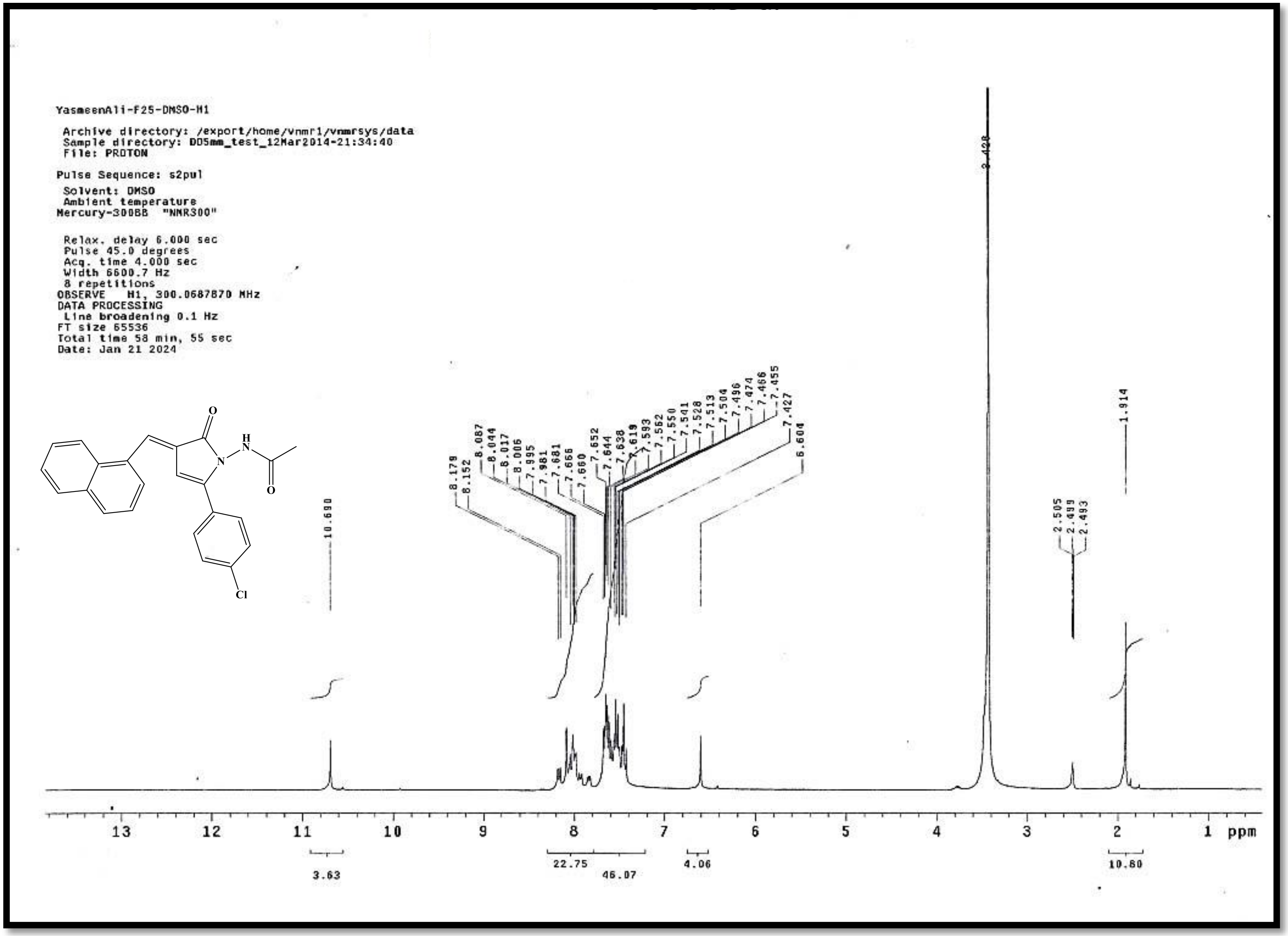

Figure 56S. ¹H-NMR (DMSO- d₆) of Compound (17)

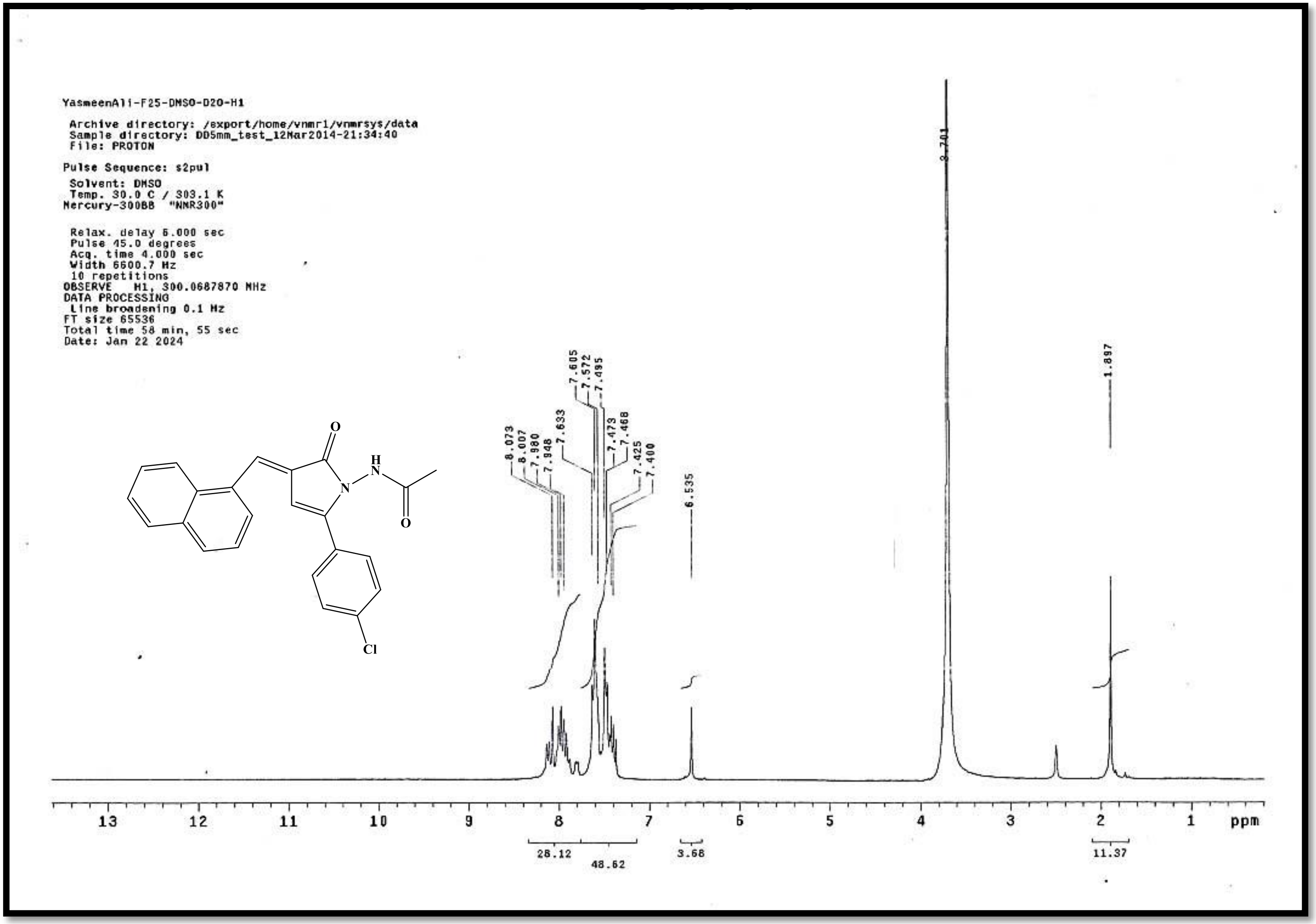

Figure 57S. ^{1}**H-NMR spectrum (DMSO-d$_6$ + D$_2$O) of Compound (17)**

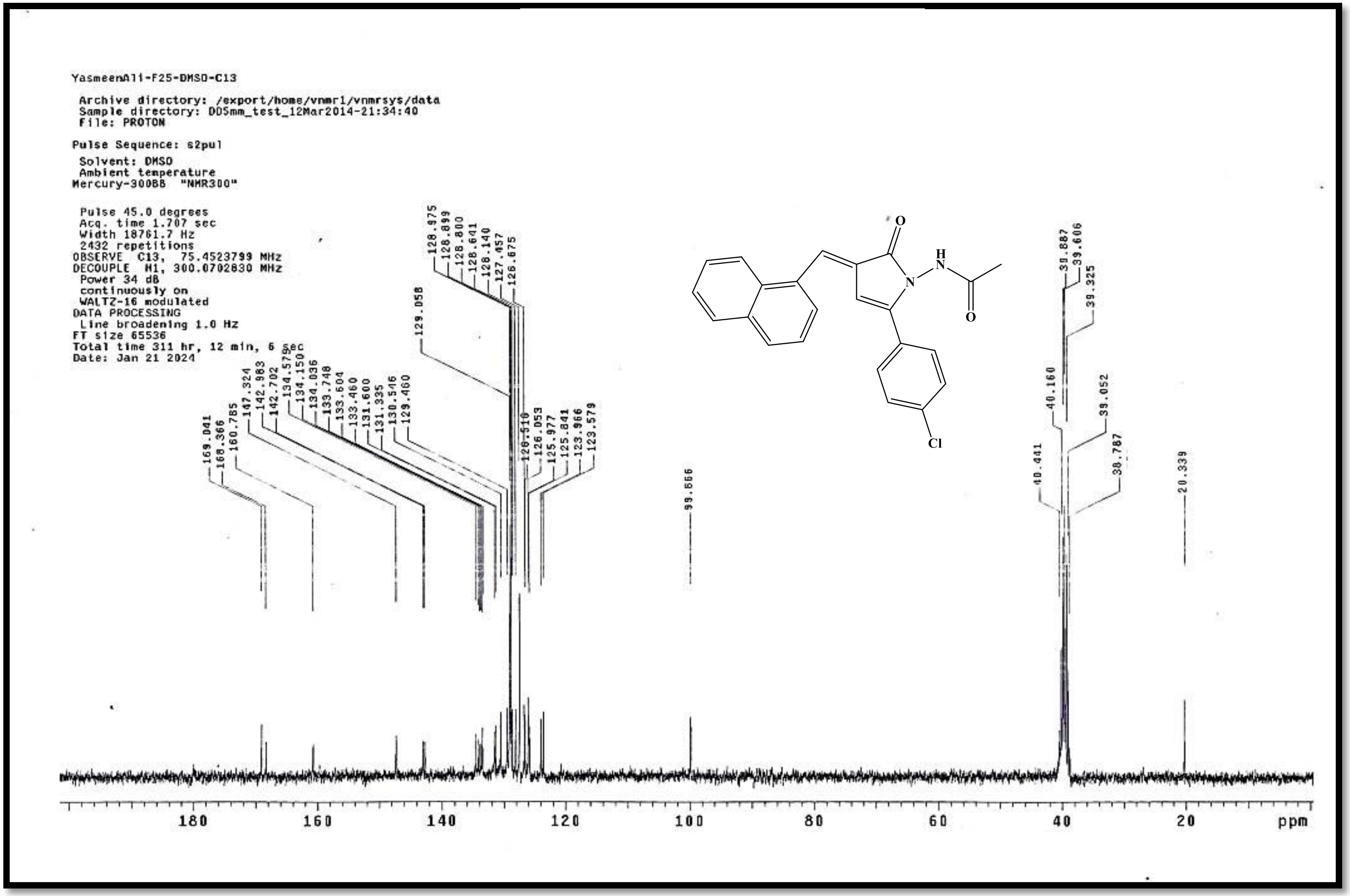

Figure 58S. ^{13}**C-NMR spectrum (DMSO-d$_6$) of Compound (17)**
